# Supplementary material for: Clonality, inbreeding, and hybridization in two extremotolerant black yeasts
Source: Gigascience. 2022 Oct 6;11:giac095. doi: 10.1093/gigascience/giac095 (PMC9535773; doi:10.1093/gigascience/giac095)
Supplement: giac095_GIGA-D-22-00073_Original_Submission [file giac095_giga-d-22-00073_original_submission.pdf]

GigaScience

Clonality, inbreeding, and hybridization in two extremotolerant black yeasts

--Manuscript Draft--

**Manuscript Number:**	GIGA-D-22-00073
**Full Title:**	Clonality, inbreeding, and hybridization in two extremotolerant black yeasts
**Article Type:**	Research
**Funding Information:**	Javna Agencija za Raziskovalno Dejavnost RS (MRIC UL I0-0022) — Not applicable<br>Javna Agencija za Raziskovalno Dejavnost RS (P4-0432) — Assoc Prof Cene Gostinčar<br>Javna Agencija za Raziskovalno Dejavnost RS (P1-0198) — Not applicable<br>Javna Agencija za Raziskovalno Dejavnost RS (J4-2549) — Assoc Prof Cene Gostinčar
**Abstract:**	The great diversity of lifestyles and survival strategies observed in fungi is reflected in the many ways in which they reproduce and recombine. Although truly asexual fungi are rare, population genomic data support the clonality of two extremotolerant black yeasts from Dothideomycetes: Hortaea werneckii and Aureobasidium melanogenum. Thus, the discovery of a number of diploid strains of these species could not be explained as the product of conventional sexual reproduction.<br>Genome sequencing revealed that the ratio of diploid to haploid strains in both H. werneckii and A. melanogenum is approximately 2:1. Linkage disequilibrium between pairs of polymorphic loci and a high degree of concordance between the phylogenies of different genomic regions confirmed that both species are clonal. Heterozygosity of diploid strains is high, with several hybridizing genome pairs reaching the intergenomic distances typically seen between different fungal species. The origin of diploid strains collected worldwide can be traced to a handful of hybridization events that produced diploids, which were stable over long periods of time and distributed over large geographic areas.<br>Our results, based on the genomes of over 100 strains of two black yeasts, show that although they are asexual, they occasionally form stable and highly heterozygous diploid hybrids. The mechanism of these apparently rare hybridization events, which are not followed by meiosis or haploidisation, remains unknown. Both extremotolerant yeasts, H. werneckii and even more so A. melanogenum, a close relative of the intensely recombining and biotechnologically relevant Aureobasidium pullulans, provide an attractive model for studying the role of clonality and ploidy in extremotolerant fungi.
**Corresponding Author:**	Cene Gostinčar, Ph.D.<br>University of Ljubljana Biotechnical faculty: Univerza v Ljubljani Biotehniska fakulteta<br>Ljubljana, SLOVENIA
**Corresponding Author Secondary Information:**	
**Corresponding Author's Institution:**	University of Ljubljana Biotechnical faculty: Univerza v Ljubljani Biotehniska fakulteta
**Corresponding Author's Secondary Institution:**	
**First Author:**	Cene Gostinčar, Ph.D.
**First Author Secondary Information:**	
**Order of Authors:**	Cene Gostinčar, Ph.D.<br>Xiaohuan Sun<br>Anja Černoša

Powered by Editorial Manager® and ProduXion Manager® from Aries Systems Corporation

	Chao Fang
	Nina Gunde-Cimerman
	Zewei Song
**Order of Authors Secondary Information:**	
**Additional Information:**	
**Question**	**Response**
Are you submitting this manuscript to a special series or article collection?	No
**Experimental design and statistics**<br><br>Full details of the experimental design and statistical methods used should be given in the Methods section, as detailed in our Minimum Standards Reporting Checklist. Information essential to interpreting the data presented should be made available in the figure legends.<br><br>Have you included all the information requested in your manuscript?	Yes
**Resources**<br><br>A description of all resources used, including antibodies, cell lines, animals and software tools, with enough information to allow them to be uniquely identified, should be included in the Methods section. Authors are strongly encouraged to cite Research Resource Identifiers (RRIDs) for antibodies, model organisms and tools, where possible.<br><br>Have you included the information requested as detailed in our Minimum Standards Reporting Checklist?	Yes
**Availability of data and materials**<br><br>All datasets and code on which the conclusions of the paper rely must be either included in your submission or deposited in publicly available repositories (where available and ethically	Yes

appropriate), referencing such data using a unique identifier in the references and in the “Availability of Data and Materials” section of your manuscript.<br><br>Have you have met the above requirement as detailed in our Minimum Standards Reporting Checklist?	

Powered by Editorial Manager® and ProduXion Manager® from Aries Systems Corporation

Main manuscript Click here to access/download;Manuscript;220408 Manuscript.docx

Clonality, inbreeding, and hybridization in two extremotolerant black yeasts

Cene Gostinčar [a,b,¶,*], Xiaohuan Sun [c,¶], Anja Černoša [a], Chao Fang [c], Nina Gunde-Cimerman [a,+], Zewei Song [c,+]

[a] Department of Biology, Biotechnical Faculty, University of Ljubljana, 1000 Ljubljana, Slovenia

[b] Lars Bolund Institute of Regenerative Medicine, BGI-Qingdao, Qingdao 266555, China

[c] BGI-Shenzhen, Beishan Industrial Zone, Shenzhen 518083, China

¶ *These authors contributed equally to this work as first authors.*

+ *These authors contributed equally to this work as leading authors.*

*** Corresponding author: Cene Gostinčar**
Department of Biology, Biotechnical Faculty
University of Ljubljana
Jamnikarjeva 101
SI-1000 Ljubljana
Slovenia
Tel.: +386-1-320 3392
Fax: +386-1-2573390
E-mail: cene.gostincar@bf.uni-lj.si, cgostincar@gmail.com

Xiaohuan Sun: sunxiaohuan@genomics.cn
Anja Černoša: anja.cernosa@bf.uni-lj.si
Chao Fang: fangchao@genomics.cn
Nina Gunde-Cimerman: nina.Gunde-Cimerman@bf.uni-lj.si
Zewei Song: songzewei@genomics.cn

Running Title

Hybridization in extremotolerant yeasts

Abstract

The great diversity of lifestyles and survival strategies observed in fungi is reflected in the many ways in which they reproduce and recombine. Although truly asexual fungi are rare, population genomic data support the clonality of two extremotolerant black yeasts from Dothideomycetes: *Hortaea werneckii* and *Aureobasidium melanogenum*. Thus, the discovery of a number of diploid strains of these species could not be explained as the product of conventional sexual reproduction.

Genome sequencing revealed that the ratio of diploid to haploid strains in both *H. werneckii* and *A. melanogenum* is approximately 2:1. Linkage disequilibrium between pairs of polymorphic loci and a high degree of concordance between the phylogenies of different genomic regions confirmed that both species are clonal. Heterozygosity of diploid strains is high, with several hybridizing genome pairs reaching the intergenomic distances typically seen between different fungal species. The origin of diploid strains collected worldwide can be traced to a handful of hybridization events that produced diploids, which were stable over long periods of time and distributed over large geographic areas.

Our results, based on the genomes of over 100 strains of two black yeasts, show that although they are asexual, they occasionally form stable and highly heterozygous diploid hybrids. The mechanism of these apparently rare hybridization events, which are not followed by meiosis or haploidisation, remains unknown. Both extremotolerant yeasts, *H. werneckii* and even more so *A. melanogenum*, a close relative of the intensely recombining and biotechnologically relevant *Aureobasidium pullulans*, provide an attractive model for studying the role of clonality and ploidy in extremotolerant fungi.

Keywords**:** population genomics, halotolerance, extremotolerance, halophilic fungus, *Hortaea werneckii*, *Aureobasidium melanogenum*, hybridization

Introduction

No single reproductive strategy is optimal for all species and all conditions in which they live. This results in the coexistence of a wide variety of ways in which organisms reproduce and recombine their genetic material. Among the most diverse are fungi, which exhibit a wide range of strategies, from strictly clonal species [1] to species with thousands of mating types [2]. Sexual, parasexual, and clonal reproduction are broad categories that encompass a variety of different phenomena, processes, and mechanisms, some of which are typical of larger groups (e.g., the dikaryon of basidiomycetes), while others differ even among closely related species [3].

Traditionally, up to one-fifth of fungi were thought to be asexual [4]. Subsequent genetic and genomic analyses found at least a rudimentary mating-type locus in nearly every species studied. Population genetics showed that most species previously thought to be asexual are actually recombining [5–7]. However, in most species, asexual reproduction dominates over occasional recombination. To account for the observation that some apparently clonal species can nevertheless recombine at levels low enough not to break the pattern of population structure typical of clonal species, Tibayrenc and Ayala [5] introduced the concept of "restricted recombination". Nevertheless, some species appear to be strictly clonal even by highly sensitive measures of recombination used by population genomics, such as linkage disequilibrium [1].

Several reasons for the pervasive clonality in fungi have been proposed, such as hybrid incompatibility or limited opportunities to meet strains of the opposite mating type [6]. Severe bottlenecks (e.g., following the introduction of a small number of strains to a new site) can lead to a skewed balance of mating types. Strains of pathogens with host-to-host transmission may encounter other strains of the species very rarely [6,8]. Similar may be true for fungi with poor dispersal abilities that are restricted to rare and isolated ecological islands, such as certain extreme environments [1,9]. However, even without these constraints, sexually meiotic reproductive events are often majorly outweighed by asexual mitotic events, as shown in *Saccharomyces paradoxus* [10].

The absence of recombination carries the risk of accumulation of deleterious mutations, a process known as Muller's ratchet. Sexual reproduction remedies this and efficiently generates diversity, which is a substrate for selection and adaptation to novel conditions. But asexual reproduction has its own advantages. For example, it can

conserve energy by eliminating the need to maintain the mating system and form sexual structures [11,12]. It also allows the organism to faithfully reproduce successful genomic configurations and thus avoid the recombination load, a loss of fitness due to a break up of advantageous combinations of interacting alleles [12–14]. This may be particularly beneficial in specialists, such as those inhabiting extreme environments [14,15].

In addition to sexual reproduction, fungi can employ another tool to recombine their genomes: parasexuality [16]. Two cells can fuse to combine their genetic material, providing the opportunity for mitotic recombination. Haploid parents thus produce diploid offspring, but this ploidy change is generally considered unstable. However, it does not revert to the original ploidy of the parental strains through the tightly controlled and high-fidelity process of meiosis. Instead, chromosomes are lost randomly through a series of aneuploid generations. The importance of parasexuality outside of laboratory settings has been questioned [17], but in at least some cases the process appears to drive adaptation and facilitate survival, e.g., in *Aspergillus fumigatus* in the lungs of patients with cystic fibrosis [18].

Changes in ploidy itself may be a form of adaptation, either through parasexuality or other processes such as abnormal cell division [19]. Both polyploidy and aneuploidy can be a response to adverse or novel environmental conditions [20–22]. They influence fitness through changes in cell size and shape, changes in the transcriptome (by altering gene dosage) and in the rate of adaptation, but also by providing new options for repairing DNA damage and temporarily masking deleterious mutations [19–21,23]. A growing body of evidence shows that variation in ploidy is a widespread transient adaptation of fungi to novel conditions (reviewed by Naranjo–Ortiz and Gabaldón [21]). Aneuploidies are more common and more easily reversed than tandem gene duplications, which are an alternative way to increase gene dosage. During cultivation under optimal conditions, such altered ploidies tend to revert to a baseline ploidy of the species, often without clear increases in fitness [20,24].

Sexual and parasexual recombination can lead to recombinant lineages and interspecies hybrids [25], another process increasingly recognised as an important generator of fungal diversity, including in industrial and clinical settings (reviewed by Naranjo–Ortiz and Gabaldón [21]). With the increasing accessibility of genome sequencing, research on this topic is rapidly expanding to non-model species. Some

hybrids exhibit higher fitness than their parental strains [26], making hybridization an important driver of adaptation to novel environments [21,27]. Divergent hybrid genomes can be stabilised by chromosomal aberrations [28,29] and the outcome of hybridization is often similar to parasexuality. Hybrids of *Cryptococcus neoformans* and *Cryptococcus gattii*, for example, rapidly lose chromosomes and rearrange them [28,29].

Indications of hybridization were also reported by Gostinčar et al. [1] in the extremely halotolerant black yeast *Hortaea werneckii* (Capnodiales, Dothideomycetidae, Dothideomycetes, Pezizomycotina, Ascomycota), a globally distributed species specialised for survival in saline environments and able to grow in nearly salt-saturated solutions [30,31]. Whole genome sequencing of twelve strains indicated that the species is clonal, but also that a majority of the strains are highly heterozygous diploids. These diploids appeared to be stable enough to spread over considerable distances, with little evidence of haploidisation [1,32]. This explained the ploidy of the reference genome, which was originally interpreted as the result of endoreduplication [33,34]. Subsequent genome sequencing of two additional *H. werneckii* strains provided additional support for the hybridization hypothesis [35]. However, the total number of sequenced *H. werneckii* genomes remained low, limiting the power of the analyses and the interpretation of the results.

A similar pattern of haploid and diploid strains coexisting within an apparently clonal species has since been discovered in *Aureobasidium melanogenum* (Dothideales, Dothideomycetidae, Dothideomycetes, Pezizomycotina, Ascomycota), another black yeast only distantly related to *H. werneckii* [36]. While *A. melanogenum* tolerates less extreme conditions than *H. werneckii*, it is tolerant of a wider range of types of stress and occurs in a variety of environments, from hypersaline waters to various indoor habitats (reviewed by Černoša et al. [36]).

The role of hybridization and ploidy changes are among the overlooked dimensions of fungal genetics [21]. Here we analyse 66 genomes of *H. werneckii* and 49 genomes of *A. melanogenum* to provide insight into the reproductive strategy of these two extremotolerant fungi, characterised by coexistence of haploid and highly heterozygous diploid strains that are stable over large geographic and temporal distances.

Results

Whole genomes of 54 strains of the extremely halotolerant black yeast *H. werneckii* were sequenced. Combined with previously sequenced strains [1,34], this resulted in a data set of 66 whole-genome sequences (Table 1, Supplemental Table S1). A majority of strains (26) were isolated from brine, evaporation-concentrated seawater during salt extraction; followed by strains isolated from bittern (7), a saturated, magnesium-rich solution that remains after precipitation of halite during salt extraction. Nine strains were isolated from a seacoast cave in Atacama, where some of the strains grew on spider webs along with the alga *Dunaliella atacamensis* [37]. Twelve strains were isolated from marine habitats and four were clinical isolates. All genomes of *A. melanogenum* (Table 2) were sequenced and described in a previous study [36]. The largest number of strains (19) were isolated from bathroom and kitchen surfaces (including from kitchen appliances), followed by 16 strains from tap water or springs of tap water. In the case of both *H. werneckii* and *A. melanogenum*, the majority of strains were isolated in Slovenia.

Based on previous studies [1,33,34], the haploid genomes of both *H. werneckii* and *A. melanogenum* are approximately 25 Mbp in size. Comparing the sizes of genome assembly and the number of predicted genes in each genome, 20 (30%) *H. werneckii* genomes were recognised as haploid, 45 (68%) as diploid, and one genome as tetraploid. This was similar to the *A. melanogenum* genomes where 16 (33%) genomes were recognised as haploid, 30 (61%) as diploid, and the ploidy of three genomes (2, 18, 38) was unclear [36].

The assembly size, number of predicted genes and other genomic characteristics of *H. werneckii* were fairly consistent within groups of specific ploidy (Table 3). The average genome assembly size was 26.52 Mbp (±1.47 SD) for haploid and 49.30 Mbp (±1.74 SD) for diploid genomes. The average number of predicted genes was 9519 (±665 SD) for haploids and 20417 (±1709 SD) for diploids. As expected, the quality of assembly was much lower in diploid strains, as evidenced by a higher number of contigs and a smaller N50 value, presumably due to regions of high similarity between the two haploid subgenomes, a long-standing challenge in sequencing *H. werneckii* genomes [34]. Nevertheless, the assembly and annotation of all strains were of

reasonable quality, with only about 3.19% (±0.30 SD) Benchmarking Universal Single-Copy Orthologs missing completely in the haploid genomes and 6.09% (±4.24 SD) in the diploid genomes.

Single-nucleotide polymorphism (SNP) analysis was performed on all strains except *H. werneckii* strain 36 due to its tetraploid genome. The average density of single nucleotide polymorphisms (SNPs) in haploid strains of *H. werneckii* was high: 4.04% (±1.11 SD) (Table 3). For diploids, the SNP density was 3.44% (±1.12 SD), of which 71% of the loci were heterozygous. In *A. melanogenum* the average SNP density was 4.41% (±1.87 SD) in haploids and 3.79% (±0.21 SD) in diploids, with 44% of the latter heterozygous. Based on the SNP data, the genomes of both *H. werneckii* and *A. melanogenum* were clustered into 5 clusters in principal component analysis, with the first two principal components explaining 57.2% of the SNP diversity of *H. werneckii* and 59.3% of *A. melanogenum* (Fig. 1). SNP-based phylogenetic analyses of both species revealed considerable reticulation (Fig. 1). The largest cluster of strains identified by both network analysis and PCA contained 18 strains in *H. werneckii* and 20 strains in *A. melanogenum*.

The squared correlation coefficient (r^2) was calculated for all pairs of biallelic SNP loci present in at least two genomes analysed and within 10 kbp of each other. Plotting r^2 as a function of the distance between pairs of loci showed very little decay of linkage disequilibrium in either species. Linkage disequilibrium remained high above half of the maximum value even for alleles that were 10 kbp apart (Fig. 2). Such strong linkage between loci can be explained by a lack of recombination that would break the linkage, confirming previous reports that *H. werneckii* and *A. melanogenum* are strictly clonal [1,36].

The phylogenies of the fifty longest alignable genomic regions were also consistent with the presumed lack of recombination within *H. werneckii* and *A. melanogenum*. The phylogenetic trees showed a high degree of concordance (Fig. 3), meeting the “strong phylogenetic signal” criterion for clonality [5]. Sequences representing different haploid subgenomes of diploid strains were positioned in different parts of the phylogeny, corresponding to the high heterozygosity of the strains. An extreme case of this was the tetraploid *H. werneckii* strain 36, which was positioned in four different parts of the phylogeny. When all 50 multi-labelled trees for each species were

collapsed into a consensus supernetwork (Fig. 3), the result was similar to the phylogenetic network estimated from SNP data (Fig. 1).

The topology of the consensus phylogenies and supernetworks was best explained by a number of hybridization events in each species: 9 or 10 events in the case of *H. werneckii* (with an additional event leading to tetraploid strain 36) and 7 events in the case of *A. melanogenum* (Fig. 4). Several phylogenetic lineages resulting from these events appeared to be relatively widespread – more than one representative strain was found for most lineages, often in different habitats and geographic locations. However, lineage composition was skewed in favour of specific localities or habitats. This was confirmed by Fisher's Exact Test, which found significant differences between groups in both the isolation habitat and the geographic location of origin for both species ("*H. werneckii* – habitats": $p < 0.01$; all other: $p < 0.001$). For example, for both species, the two largest groups (groups 1 in Fig. 4) contained isolates from Europe, with only one exception. In terms of habitats, *H. werneckii* group 1 was isolated mainly from hypersaline habitats and group 9 from seawater; groups 4 and 5 were found mainly in a cave on a desert coast. The largest group of haploid strains was also found mainly in hypersaline habitats. Clinical isolates of *H. werneckii* belonged to different phylogenetic lineages. The tetraploid *H. werneckii* strain 36 was isolated from the deep sea (Figs. 3, 4) and most likely arose by hybridization between diploid hybrids of groups 1 and 9. In the case of *A. melanogenum*, most strains originated in Europe. Some groups showed preference for particular habitats: *A. melanogenum* group 1 was mostly isolated from tap water and its sources, while the majority of isolates from household surfaces were classified into other groups.

Aneuploidy in the genomes of *H. werneckii* and *A. melanogenum* was investigated by searching for large genome segments with different sequencing coverage than the rest of the genome. Evidence of aneuploidy was found in 23 genomes of *H. werneckii* (35%) and 8 genomes of *A. melanogenum* (16%) (Fig. 5, Supplemental Figs. S1, S2). The majority of these genomes were diploid: 18 (78%) in the case of *H. werneckii* and 8 (100%) in the case of *A. melanogenum*. Some parts of the genome were aneuploid in several strains, with most aneuploid parts representing an increase in ploidy rather than a decrease. The aneuploid strains included three of four clinical *H. werneckii* isolates and the only clinical isolate of *A. melanogenum* in the study. In some diploid genomes loss of heterozygosity was observed over large regions (Supplemental Fig.

S3). Some of these could be explained by aneuploidy (loss of one copy of a chromosome or part of chromosome), while others appeared to be copy-neutral, possibly caused by mitotic recombination.

A putative mating-type locus was found in the majority of the genomes, although in many cases it was poorly assembled, especially in diploid genomes and even more so in the tetraploid genome of *H. werneckii*, precluding a conclusive analysis in all strains. However, where entire genes MAT1-1 and MAT1-2 were assembled, they showed a substantial diversity, clustering into distinct phylogenetic groups (Supplemental Figs. S4, S5). In case of *H. werneckii* 'blastx' searches against the non-redundant GenBank protein database showed that one large phylogenetic group of each locus was highly similar to homologues from other fungi ("true" MAT1-1 and MAT1-2), while the other groups only produced matches with these "true" *H. werneckii* homologues, but not with homologues from other fungi, especially in case of MAT1-2, a result of an intense diversification. In the case of *A. melanogenum*, all putative MAT1-1 and MAT1-2 could be matched to homologues from other fungi. Phylogenetic groups of MAT1-1 and MAT1-2 generally corresponded to hybrid and haploid groups of strains, but with numerous exceptions (Supplemental Figs. S4, S5). For example, in case of *H. werneckii* hybrid groups generally contained two similar copies of MAT1-1, but groups 1a, 3 and 4 contained very different copies and group 9 consistently lacked one copy of MAT1-1 altogether (possibly due to poor assembly of the locus). In contrast, two different copies of MAT1-2 were found in group 1, but two similar copies in group 9. Three distinct copies of the mating-type locus were found in strain 10. In *A. melanogenum* hybrid group 1 contained only a single well-assembled homologue of MAT1-1 and two homologues of MAT1-2, but otherwise the diversity of the mating-type locus in this species was generally lower and mostly corresponded to groups of hybrids and haploid strains.

The search for proteins of the Pfam families HET and Het-C typical of heterokaryon incompatibility proteins, identified a large number of such proteins in the predicted proteomes of *A. melanogenum* (on average 4.87 HET and 3.74 Het-C proteins per strain) and even more in *H. werneckii* (on average 27.82 HET and 3.5 Het-C proteins per strain) (Supplemental Table S2). Phylogenetic analysis of these proteins showed that they formed several clusters, some of which contained representatives only from specific groups of hybrid strains (as identified in Fig. 4). For example, in *H. werneckii*,

the hybrid genomes of groups 9 and 10 (and in one case genome 23) were the only ones to contain HET genes belonging to phylogenetic clusters 6 and 24 (Supplemental Table S2). HET proteins from cluster 12 were found only in hybrid groups 1-3 and their closely related haploid strains (and tetraploid strain 36). In *A. melanogenum*, hybrid groups 5 and 6 were the only ones to contain HET proteins belonging to a small phylogenetic cluster 5 (Supplemental Table S2) and similarly hybrid group 2 was the only one to contain HET proteins from phylogenetic group 6.

Discussion

Genome sequencing of 66 strains of the black yeast *Hortaea werneckii* and 49 strains of the black yeast *Aureobasidium melanogenum* revealed some unexpected similarities between these species, which belong to different orders of the subclass Dothideomycetidae. Approximately one-third of the sequenced strains of each species were haploid and approximately two-thirds were diploid. Principal component analysis of single-nucleotide polymorphisms identified several clusters of strains in each species. In both cases, the first two principal components explained nearly 60% of the observed diversity – much more than, for example, in the homogenous and recombining species *Aureobasidium pullulans*, where the first two principal components together explained less than 15% of the diversity [38]. The clustering of strains was consistent with the previous reports that both *H. werneckii* and *A. melanogenum* are strictly clonal [1,36]. Despite the presence of a mating-type locus in the reference genomes of both species [1,39], the clonality of the species was confirmed here by a high degree of concordance between the phylogenetic histories of different genomic regions and by a lack of decay in linkage disequilibrium, an established measure of recombination often expressed as the distance over which linkage disequilibrium falls to half its maximum value [6].

The existence of highly heterozygous hybrids, first observed in *H. werneckii* [1], is confirmed here on a much larger genomic data set of *H. werneckii* and also *A. melanogenum*. The mechanism of hybridization is unknown and could range from vegetative hyphal fusion between different haploid strains to plasmogamy and karyogamy of gametes. Regardless of the mechanism, at least some diploid hybrids appear to be stable over long periods of time, allowing them to disperse over long

distances and constitute a large proportion of the species in some habitats. Hybrid strains have previously been reported in many other fungal species, including *Saccharomyces cerevisiae* [40], *Candida tropicalis* [41], and *Cryptococcus neoformans* [42], but none of these species are strictly clonal. However, diploid hybrids that cannot reproduce sexually have also been reported in some species [43]. The majority of *Candida orthopsilosis* are hybrids with 5% divergence between their haploid genomes at the nucleotide level, arising from at least four independent hybridization events [44]. *Candida metapsilosis* is also a species originating from hybridization, with a similar divergence between haploid genomes [45]. It is possible that *H. werneckii* and *A. melanogenum* follow a similar reproductive strategy as the *Candida parapsilosis* / *C. orthopsilosis* / *C. metapsilosis* group, but in the case of both black yeasts the hybrids are not recognised as separate species (for reasons discussed below).

In the reticulate history of *H. werneckii* and *A. melanogenum,* the unit of genetic exchange are whole haploid genomes. This allowed us to trace phylogenies of haploid genomes, for example, in the case of *H. werneckii* (Fig. 4A) of the "green haploid" genome (strains 2 and 3) and the "red haploid" genome (strains 4 and C), as well as their "red and green diploid" hybrids (group 1). The absence of haploid strains with genomes combined from two or more genomic lineages implies that – as is often seen in hybrids – diploids do not revert to haploid state but are stuck at the F1 stage. They are either unable to undergo meiosis, or the progeny of such meiosis has sufficiently low viability or fitness to evade sampling. The presence of a putative homothallic mating-type locus in the majority of sequenced strains and the distribution of diversified lineages of MAT genes in groups of hybrids and haploids raise the possibility that these loci play a role in the formation of hybrid lineages. At the same time a substantial number of strains containing unexpected combinations of MAT lineages, the fast diversification of the MAT genes (particularly in *H. werneckii*), and the methodological limitations of the analysis (suboptimal assembly of the putative mating-type locus), demand further research before providing conclusive evidence on the presence, type and functionality of mating-type genes, and their possible role in the formation of hybrids in *H. werneckii* and *A. melanogenum*.

While hybridization to diploids and the absence of meiosis is reminiscent of parasexuality, *H. werneckii* and *A. melanogenum* do not conform to this mode of reproduction either. In parasexual reproduction diploids typically revert to haploids

through haploidisation – a random loss of chromosomes with the end result similar to meiosis [17]. In addition to the absence of recombinant haploid strains there is also little evidence for the existence of intermediate aneuploid states characteristic of haploidisation. Either such haploidisation does not occur, or its products are not viable due to incompatibility of parental chromosomes. The aneuploidy observed in both *H. werneckii* and *A. melanogenum* mostly involves increases in ploidy above the diploid state rather than decreases below it, as would be expected with haploidisation. Thus, this aneuploidy is almost certainly not part of a parasexual cycle, but might be an adaptive evolutionary response to adverse or novel conditions – a common adaptive response of fungi [20–22]. This explanation is also supported by the observations of large-scale duplications of specific genomic regions in a diploid strain of *H. werneckii* subjected to long-term experimental evolution at extreme salinity [46]. Interestingly, the high level of heterozygosity of diploid strains of *H. werneckii* and *A. melanogenum* resulting from hybridisation is mostly preserved not only by the rarity of large-scale deletions, but also by the relative scarcity of large-scale copy-neutral loss of heterozygosity. In other hybrids loss of heterozygosity has been recognised as a common tool for genome shaping and stabilisation after hybridization [47,48], but appears to be largely avoided by both *H. werneckii* and *A. melanogenum*.

An integral part of fungal parasexuality is the heterokaryon – a cell with two genetically distinct nuclei that sometimes undergo karyogamy [49]. In *H. werneckii* both haploid and diploid cells contain a single nucleus per cell [50]. The viability and stability of heterokaryons are controlled by heterokaryon incompatibility loci. At least three genes for proteins with domains characteristic of such loci were found in all *A. melanogenum* genomes and at least 17 in *H. werneckii* (with up to 43 in diploid genomes, although this number may be an overestimate due to fragmented genome assembly). While it has been shown in some species that even differences in heterokaryon incompatibility loci as small as a single amino acid can be sufficient to trigger incompatibility [51], the diversity of these loci in *H. werneckii* and *A. melanogenum* was much higher than that. The distribution of certain types of these loci is consistent with the hybrid groups described above. On the one hand, this could simply reflect the phylogenetic distance between the strains. On the other hand, it opens the possibility that heterokaryon incompatibility loci might be involved in the successful formation of diploid hybrids in *H. werneckii* and *A. melanogenum*.

Hybrid fungal genomes have so far been described mostly in pathogens of animals or plants. This is the first time we document the formation of stable and highly heterozygous diploids in wild populations of two extremotolerant clonal species. Five aspects of this phenomenon are discussed below.

1. Is clonality related to the extremotolerance of *H. werneckii* and *A. melanogenum*? It has long been speculated that asexuality may be advantageous in extreme environments, conserving the energy required for sexual reproduction and allowing the fixation of beneficial alleles and genomic configurations in small populations that have managed to adapt to extreme conditions at the ecological edge of the species [14,15]. One of the mechanisms that can promote adaptation at the margin of species distribution is hybridization [52].

Of course, sexual reproduction does not only have shortcomings, but also considerable advantages in adapting to stress [6,12]. The same is true for parasexuality, which not only alters ploidy but also increases diversity through cycles of regular and double ploidy. A high frequency of diploids in *Aspergillus fumigatus* has been reported in cystic fibrosis, presumably due to local stress (e.g., nitrogen deficiency or the presence of certain drugs) that promotes parasexual recombination [18]. In *Candida albicans*, which can undergo regular sexual recombination, stress additionally promotes the parasexual cycle, which generates a high degree of diversity, including aneuploidy [53].

There are also several examples of at least occasionally recombining extremotolerant and extremophilic species. For example, the polyextremotolerant yeast *Aureobasidium pullulans*, a close relative of *A. melanogenum*, has one of the highest rates of recombination demonstrated in fungi by population genomics [38]. Two salt-adapted basidiomycetes, the halotolerant *Wallemia mellicola* and the halophilic *Wallemia ichthyophaga*, also appear to recombine, albeit much less frequently than *A. pullulans*, even though *W. ichthyophaga* is exceptionally rare and limited to highly fragmented environments [9,54]. If clonality or hybridization are indeed beneficial for adaptation to extreme conditions – and more data are needed to test this hypothesis – recombination appears to be compatible with extremotolerant lifestyle as well.

2. Do clonality and hybridization allow for greater specialisation? While recombination generates potentially useful diversity and thus provides substrate for natural selection, it can also break successful genomic configurations – a shortcoming known as recombination load [13–15]. In well-adapted subpopulations clonality prevents beneficial adaptations from being diluted by gene flow from other environments, which promote different adaptations. Clonal lineages may thus be more successful in the short term, but may collapse due to reduced adaptability or Muller's ratchet and are replaced by the next successful clone, which can be generated by sexual or parasexual recombination – or hybridization [55].

Some species are able to thrive in a wide range of different environments without adapting to any of them at the genomic level – the ubiquitous and polyextremotolerant *A. pullulans* is one such example [38]. But while *A. pullulans* is an exceptionally generalistic species, the two species analysed here are less so: *A. melanogenum* is mostly restricted to aquatic and indoor environments, while *H. werneckii* is mostly found in marine and hypersaline environments and has a much higher upper salinity growth limit than *A. pullulans*. The preference of some *H. werneckii* and *A. melanogenum* strain groups for specific habitats (Fig. 4) possibly indicates an ongoing clonality-driven specialisation of these groups. This would be in line with the suggestion of Romeo et al. [35]. However, based on the here studied dataset the observed habitat preferences might be an artefact of skewed geographic distribution due to limited dispersal and unequal habitat sampling in different locations.

Interestingly, the clinical isolates of *H. werneckii* belong to different strain groups within the species (Fig. 4). Although the number of clinical isolates analysed here was too small to draw reliable conclusions, this could mean that no lineage within the species is better able to cause infections in humans than others. Aneuploidy was observed in four out of five analysed clinical strains of both species. As discussed above, aneuploidy can be a signature of adaptation to novel environments [20–22]. Both *H. werneckii* and *A. melanogenum* are opportunistic pathogens that rarely cause infections. The conditions they encounter in the human body are almost certainly suboptimal for their survival [56,57], resulting in high selection pressure and possibly in aneuploidies. However, due to their rarity, such infections most likely do not contribute meaningfully to the evolution and specialisation of either of the two species [56].

3. If the formation of diploids in *H. werneckii* and *A. melanogenum* is irreversible, what drives the co-existence of diploid and haploid strains? A study of 12 *H. werneckii* genomes reported 7 successful hybridization events, and expansion of the data set to 66 genomes uncovered only 2 or 3 additional hybridizations in the history of the species. While isolation of strains from novel environments or geographic locations might lead to the discovery of new hybrid lineages, their number is likely to remain limited. This might indicate that hybridization events are relatively uncommon or that only a small number of them result in offspring with sufficient fitness to persist in the environment. The co-existence of haploid and diploid strains may be supported by their divergent performance in different conditions. A preliminary comparison of halotolerance between diploid and haploid strains shows slightly higher halotolerance of diploid *A. melanogenum*, but no such difference in *H. werneckii* (our unpublished data), but this comparison was limited to only one parameter tested in a laboratory setting. The possibility of different adaptation value of haploids vs. diploids should be more carefully addressed in the future, for example by competition experiments.

Whatever the mechanism of hybridization, it appears to operate almost exclusively between haploid strains, and compared to many other fungal species [20] the range of observable ploidies in *H. werneckii* and *A. melanogenum* is modest. Although a randomly selected environmental strain is about twice as likely to be diploid as haploid, a single tetraploid strain of *H. werneckii* is the only evidence that these diploids can hybridize further. No triploid strains of either species have been found. Either the fusion of diploid cells is prevented by some as yet unknown mechanism, or the resulting strains do not persist in the environment long enough to be detected.

4. How should the hybrids of clonal species be treated in taxonomy? In such situation even the definition of otherwise well-established terminology is not trivial. For example, Naranjo–Ortiz and Gabaldón [21] defined hybrids as lineages emerging from ancestors, which differ from each other more than the most distant strains of well-recognised species. According to Boekhout et al. [47], lineages of interspecies hybrids can be recognised as separate species, while intraspecies hybrids better fit in the concept of varieties. This returns us to the problem of species delineation in clonal taxa, which may involve arbitrary decisions. On the one hand, diversity in *H. werneckii* is high, and distances between genomes of some strains are substantially greater than what is typical for fungal species [58]. On the other hand, Fig. 4 clearly illustrates why

a more fragmented taxonomy of *Hortaea* would result in unpractical taxonomic inflation. If hybrids and the remaining monophyletic groups of haploids were treated as species, *H. werneckii* could easily be split into 15 or more new species. In several of these new species two different sequences of standard taxonomic markers carried by a single diploid strain [32] would in many cases belong to different species – a decidedly untenable situation. Similarly, if all haploid strains were treated as one species and all diploids as another, such species would be polyphyletic. Since clonality precludes the application of the biological species concept to *H. werneckii*, we suggest the dense reticulation of its phylogeny can be pragmatically interpreted as an analogue of interbreeding. Thus, a single *H. werneckii* species is maintained despite its high diversity, as suggested also by a recent in-depth phylogenetic study of the taxon [32]. Although a comparably detailed taxonomic revision of *Aureobasidium melanogenum* is still pending, it is expected to lead to a similar outcome.

5. How common is hybridisation in clonal fungi? Clonality itself appears to be much rarer in fungi than once believed, but population genomic studies of *Neurospora* spp. showed that even closely related species can differ substantially in their reproductive strategies [3]. This is also the case here: while *A. melanogenum* is clonal, the closely related species *A. pullulans* has exceptionally high recombination rates [38]. Genome sequencing of another species of the genus, *Aureobasidium subglaciale*, revealed a small number of apparently clonal diploid strains that may belong to a new species [36], the reproductive strategy of which should be investigated if more such strains can be isolated and sequenced.

Of five fungal species from extreme environments that we have previously studied using population genomics, two were strictly clonal and both contained stable diploid hybrids. This situation is at least similar to the one described in the *Candida parapsilosis* / *C. orthopsilosis* / *C. metapsilosis* group [48]. Other examples may include the plant pathogen *Verticillium longisporum* [59], and some clinically relevant hybrids of *Cryptococcus* spp. [60] and *Aspergillus* spp. [61]. Such reproductive strategy may thus be more common than currently known, especially since it can be easily overlooked without performing careful population genomic studies. Polyploid strains often produce highly fragmented but otherwise inconspicuous assemblies, and even after genome sequencing, hybrids, polyploids, and aneuploids can easily go

undetected [21]. Any study that discovers genomes of different ploidy in clonal fungal species should investigate hybridization as a possible explanation for the data.

Conclusions

Genome sequencing of two black yeasts from extreme environments, *H. werneckii* and *A. melanogenum*, revealed that both species are strictly clonal. Their populations consist of both haploid and diploid strains, and diploid strains were formed by a handful of hybridization events between haploids. These hybridizations were not followed by meiosis as part of sexual reproduction, nor by haploidisation through random chromosome loss, as is typical of parasexuality. Hybrid lineages avoid the loss of heterozygosity even over timeframes allowing them to disperse over large geographic distances. Such “stable parasexuality”, a process of forming stable and highly heterozygous diploids in a clonal species without evidence of subsequent meiosis or haploidisation, is an unusual reproductive strategy, which merits further study. This is the first time it has been documented in wild populations of extremotolerant fungi. The increasing use of population genomics in fungi will show whether this reproductive strategy is more widespread than is currently known and careful comparative studies should investigate its potential role in adaptation to extreme (and other) environments.

Materials and methods

Cultivation and DNA isolation

Strains of the extremely halotolerant *Hortaea werneckii* (Table 1) were obtained from the Ex Culture Collection of the Department of Biology, Biotechnical Faculty, University of Ljubljana (IC Mycosmo, MRIC UL, Slovenia). The cultivation and DNA isolation were performed as described previously [1], using the standard chemically defined Yeast Nitrogen Base medium (Qbiogene). Biomass harvested from liquid cultures was frozen in liquid nitrogen and kept at -80 °C until DNA isolation, performed as described previously [1], using the UltraClean Microbial DNA isolation kit (MO BIO Laboratories, USA), preceded by homogenisation with a pestle and mortar in liquid nitrogen and 1 min in Retsch Mixer Mill 301 (ThermoFisher Scientific, USA) at 20 Hz. After the RNAse A treatment (ThermoFisher Scientific, USA), the isolated DNA was evaluated using agarose electrophoresis and by fluorometry (Qubit; ThermoFisher Scientific, USA).

Genome sequencing

The genome sequencing was performed using the platform BGISEQ-500, with 2×150-bp libraries, prepared as described previously [62], with multiplexed sequencing barcodes. The resulting output was demultiplexed, the quality was checked with FastQC, and the reads were trimmed for adaptors and quality (removal of bases with Q <20) using the 'bbduk' script (https://jgi.doe.gov/data-and-tools/bbtools/).

The raw sequencing reads have been deposited in China National GeneBank Sequence Archive (CNSA) of China National GeneBank DataBase (CNGBdb) with accession number CNP0001993. Sequencing reads together with assembly and annotation data have been deposited in Genbank under BioProject PRJNA428320. Genome sequences of previously sequenced *H. werneckii* strains [1] are deposited in Genbank under the same BioProject (PRJNA428320). Genome sequences of previously sequenced *A. melanogenum* strains [36] are deposited in Genbank under the BioProject PRJNA721240 and listed in Table 2. Genome 7 from the study by Černoša et al. [36] was excluded from this study due to the large phylogenetic distance from other *A. melanogenum* strains, while strains 2, 18, and 38 were excluded from phylogenetic analyses based on alignments produced by SibeliaZ (described below) due to their unclear ploidy.

Variant calling

Sequencing reads of *H. werneckii* genomes were mapped to the reference genome of *H. werneckii* EXF-2000 (GenBank MUNK00000000.1 [34]), which was first haploidised with HaploMerger2 [63]. Mapping was performed by 'bwa mem', using the default parameters. The reads were sorted with Samtools 1.6 [64], deduplicating with Picard 2.10.2 and then used for variant calling with Genome Analysis Toolkit 4.1 [65], following the 'Genome Analysis Toolkit (GATK) Best Practices' workflow in diploid mode, but using 'hard filtering' with the expression 'QD < 2.0 || FS > 20.0 || SOR > 3.0 || MQ < 50.0'. Strain 36 was excluded from the analysis due to its tetraploid genome. Variants of *A. melanogenum* genomes were determined by Černoša et al. [36].

Variant-based analysis

Variant-based analyses for both *H. werneckii* and *A. melanogenum* were performed in R [66], except the calculation of the linkage disequilibrium squared correlation coefficient (r^2; described below). Genomes were clustered based on the single-nucleotide polymorphism data using the principal component analysis with the ‘glPca’ function of the ‘adgenet’ package in R [67]. The phylogenetic networks estimated from SNP data were reconstructed with the Neighbor-Net algorithm of the package ‘phangorn’ [68] based on a dissimilarity distance matrix calculated with the package ‘poppr’ [69].

Linkage disequilibrium squared correlation coefficient (r^2) was calculated for all pairs of biallelic SNP loci within 10 000 nucleotides of each other with ‘vcftools’ [70]. Then r^2 was plotted as a function of distance between pairs of loci using ‘ggplot2’ [71]. A generalized additive model (“gam”) curve was fitted to the data.

Assembly and annotation

Reference-guided genome assembly was performed for all here sequenced *H. werneckii* genomes with IDBA-Hybrid 1.1.3 [72] using the same reference as for variant calling. The maximum k value selected was 120, the minimum support in each iteration was 2, the similarity threshold for alignment was 0.95, seed kmer was 20, maximum allowed gap in the reference was 100, and the minimum size of contigs included in the final assembly was 500. Genomes were annotated with Augustus 3.4 [73]. Augustus parameters were optimised with training using the scripts provided with the program with (i) the RNAseq data from Sinha et al. [34] deposited at GenBank Sequence Read Archive with the accession number SRP094740 and (ii) all predicted proteins of *H. werneckii* EXF-2000 (GenBank MUNK00000000.1). These hints were also used for the final annotation.

Predicted proteomes were benchmarked with the BUSCO 4.1.1 [74] using the default parameter values and the data set for Dothideomycetes [75].

The files for submission to GenBank were prepared with the Genome Annotation Generator (GAG) 2.0.1 [76]. Gene models with short coding regions (<150 bp) and/or introns (<10 bp) were removed before the submission.

Phylogenetic analyses

SibeliaZ 1.2.2 [77] was used to align parts of the genomes of both *H. werneckii* and *A. melanogenum* into multiple sequence alignments. The parameters used were k=21, a=150, b=15000. Alignments were then filtered to keep only those in which the number of sequences from each genome exactly matched the ploidy of the genome. Alignments were optimised with Gblocks 0.91 [78], using the options '-b3=10 -b4=3 -b5=n' and then inspected manually to trim the ends to the shortest sequence in the alignment and remove any alignments with long gaps in any of the sequences. Fifty longest alignments were selected for each species and each alignment was used for the estimation of the phylogenetic tree with IQ-TREE 2.0.3 using standard model selection and 1000 replicates for the SH approximate likelihood ratio test [79]. The resulting collection of 50 phylogenetic trees for each species was visualized as an overlay using 'densiTree()' from the 'phangorn' package in R [68] and as a consensus supernetwork using SplitsTree 4.16.2 [80]. These visualisations and a majority rule consensus tree calculated with the 'consensus.edges' from the package 'phytools' in R [81] were used to draw a schematic representation of phylogenetic histories of genomes in the open-source vector graphics software Inkscape 1.1 (http://inkscape.org). Enrichment of phylogenetic clusters of strains for certain geographic origin or habitat was analysed in R using Fisher's Exact Test with simulated p-value [66].

Genes with HET (PF06085) and Het-C (PF07217) domains were identified in predicted proteomes of all strains investigated in this study (Table 1,2) with 'hmmsearch' 3.3.1 and HMM profiles with default parameters from the Pfam-A.hmm database version 34.0 [82]. The identified proteins were aligned with Mafft 7.475 [83] and the alignments were used for reconstruction of phylogenies with IQ-TREE 2.0.3 using standard model selection and 1000 replicates for the SH approximate likelihood ratio test [79].

Putative mating-type loci were identified in the genomes with stand-alone BLAST 2.9.0+ [84], aligned with Mafft 7.475 [83], and annotated based on previously published annotations of mating-type loci in *H. werneckii* [1] and *A. melanogenum* [39]. Phylogeny of putative MAT1-1 and MAT1-2 homologues was estimated with IQ-TREE 2.0.3 using standard model selection and 1000 replicates for the SH approximate

likelihood ratio test [79] after first excluding all putative homologues truncated to less than 80% of expected length due to suboptimal genome assembly.

Detection of aneuploidies and loss of heterozygosity

Per-nucleotide sequencing depth of reads mapped to the reference genome as described above was calculated with Samtools 1.6 [64]. For each sequenced genome the median values of per-nucleotide depths in 30 kbp windows were plotted as proportion of the median depth of the whole genome. These values were calculated in R and visualised with ‘ggplot2’ [66,71] for 50 longest reference genome contigs in the case of *H. werneckii* and for 35 longest reference genome contigs in the case of *A. melanogenum*.

Evidence for loss of heterozygosity in diploid genomes was searched for by counting the number of heterozygous SNPs in 25 kbp windows along the longest reference genome contigs (50 in case of *H. werneckii*, 35 in case of *A. melanogenum*) and plotted as proportion of the median heterozygosity of each genome with ‘ggplot2’ [66,71]. Depth of sequencing was plotted as described above, but in 25 kbp windows, to distinguish between copy-neutral loss of heterozygosity and loss of heterozygosity caused by aneuploidy.

Acknowledgements

The authors would like to thank Yonglun Luo (Lars Bolund Institute of Regenerative Medicine, Qingdao-Europe Advanced Institute for Life Sciences, BGI-Qingdao; BGI-Shenzhen; Department of Biomedicine, Aarhus University) for supporting the work on genomics of fungi from extreme environments; Yuchong Tang (China National GeneBank, BGI-Shenzhen) for his invaluable help in project management, organization of scientific visits and facilitation of collaboration between the project partners; and Toni Gabaldón (Biomedical Research Institute (IRB), Barcelona Supercomputing Centre (BSC)) for inspiring and constructive discussions on hybridization in fungi. The authors thank Rafael R. Montalvo-Rodriguez (University of Puerto Rico) for the strains EXF-2515 and EXF-2516; Filomena de Leo (University of Messina, Italy) for the strains EXF-10508 to EXF-10512; Armando Azua Bustos (Pontificia Universidad Católica de Chile) for the strains EXF-11540, EXF-11650 and EXF-11651; Zhu-Hua Luo (Third Institute of Oceanography, China) for the strains EXF-12619 and EXF-12620; Amy Gladfelter (University of North Carolina) for the strains EXF-14591 and EXF-14592; and Christine Beemelmanns (Hans Knöll Institute, Germany) for the strain EXF-14590. We acknowledge the China National Gene Bank for the support of sequencing library preparation and shotgun sequencing. This study was supported by funding from the Slovenian Research Agency to Infrastructural Centre Mycosmo (MRIC UL I0-0022), programmes P4-0432, P1-0198, and project J4-2549.

Authors contributions

Conceptualization of the study: NGC, ZS and CG; experimental work: XS, AČ, and CF; bioinformatic analyses: CG; data curation: CG; resources: ZS, NGC; preparation of the manuscript and visualisations: CG; review and editing of the manuscript: CG, NGC, AČ, XS, CF and ZS; supervision: ZS and NGC; funding acquisition: NGC, ZS.

Declarations

Funding

The authors acknowledge the China National Gene Bank for the support of sequencing library preparation and shotgun sequencing. This study was supported by funding from the Slovenian Research Agency to Infrastructural Centre Mycosmo (MRIC UL), programmes P1-0170, P1-0198, project J4-2549, and young researcher grant to Anja Černoša.

Conflicts of interest/Competing interests

Not applicable.

Ethics approval

Not applicable.

Consent to participate

Not applicable.

Consent for publication

Not applicable.

Availability of data and material

All data used in the study are available in GenBank.

Code availability

Not applicable.

References

1. Gostinčar C, Stajich JE, Zupančič J, Zalar P, Gunde-Cimerman N. Genomic evidence for intraspecific hybridization in a clonal and extremely halotolerant yeast. *BMC Genomics*. 2018; doi: 10.1186/s12864-018-4751-5.

2. Brown A. Mating in mushrooms: increasing the chances but prolonging the affair. *Trends Genet*. 2001; doi: 10.1016/S0168-9525(01)02343-5.

3. Gladieux P, De Bellis F, Hann-Soden C, Svedberg J, Johannesson H, Taylor JW. Neurospora from natural populations: Population genomics insights into the life history of a model microbial eukaryote. In: Y. DJ, editor. *Stat Popul Genomics*. New york: Humana;

4. Hawksworth DL, Kirk PM, Sutton BC, Pegler DN. Ainsworth & Bisby's Dictionary of the Fungi. 8th ed. Oxford University Press;

5. Tibayrenc M, Ayala FJ. Reproductive clonality of pathogens: A perspective on pathogenic viruses, bacteria, fungi, and parasitic protozoa. *Proc Natl Acad Sci*. 2012; doi: 10.1073/pnas.1212452109.

6. Taylor JW, Hann-Soden C, Branco S, Sylvain I, Ellison CE. Clonal reproduction in fungi. *Proc Natl Acad Sci*. 2015; doi: 10.1073/pnas.1503159112.

7. Carreté L, Ksiezopolska E, Pegueroles C, Gómez-Molero E, Saus E, Iraola-Guzmán S, et al.. Patterns of genomic variation in the opportunistic pathogen *Candida glabrata* suggest the existence of mating and a secondary association with humans. *Curr Biol*. 2018; doi: 10.1016/j.cub.2017.11.027.

8. Kasuga T, White TJ, Koenig G, Mcewen J, Restrepo A, Castañeda E, et al.. Phylogeography of the fungal pathogen *Histoplasma capsulatum*. *Mol Ecol*. 2003; doi: 10.1046/j.1365-294X.2003.01995.x.

9. Gostinčar C, Sun X, Zajc J, Fang C, Hou Y, Luo Y, et al.. Population genomics of an obligately halophilic basidiomycete *Wallemia ichthyophaga*. *Front Microbiol*. 2019; doi: 10.3389/fmicb.2019.02019.

10. Tsai IJ, Bensasson D, Burt A, Koufopanou V. Population genomics of the wild yeast *Saccharomyces paradoxus*: Quantifying the life cycle. *Proc Natl Acad Sci*. 2008; doi: 10.1073/pnas.0707314105.

11. Xu J. Cost of interacting with sexual partners in a facultative sexual microbe. *Genetics*. 2005; doi: 10.1534/genetics.105.045302.

12. Sun S, Heitman J. Is sex necessary? *BMC Biol*. 2011; doi: Artn 56 Doi

10.1186/1741-7007-9-56.

13. Otto SP. The evolutionary enigma of sex. *Am Nat*. 2009; doi: 10.1086/599084.

14. Gostinčar C, Grube M, De Hoog S, Zalar P, Gunde-Cimerman N. Extremotolerance in fungi: evolution on the edge. *FEMS Microbiol Ecol*. 2010; doi: 10.1111/j.1574-6941.2009.00794.x.

15. Gostinčar C, Gunde-Cimerman N, Grube M. Polyextremotolerance as the fungal answer to changing environments. In: Bakermans C, editor. *Microb Evol under Extrem Cond*. Berlin, München, Boston: DE GRUYTER;

16. Pontecorvo G. The parasexual cycle in fungi. *Annu Rev Microbiol*. 1956; doi: 10.1146/annurev.mi.10.100156.002141.

17. Clutterbuck AJ. Parasexual recombination in fungi. *J Genet*. 1996; doi: 10.1007/BF02966308.

18. Engel T, Verweij PE, van den Heuvel J, Wangmo D, Zhang J, Debets AJM, et al.. Parasexual recombination enables *Aspergillus fumigatus* to persist in cystic fibrosis. *ERJ Open Res*. 2020; doi: 10.1183/23120541.00020-2020.

19. Todd RT, Forche A, Selmecki A. Ploidy variation in fungi: Polyploidy, aneuploidy, and genome evolution. *Microbiol Spectr*. 2017; doi: 10.1128/microbiolspec.FUNK-0051-2016.

20. Gerstein AC, Sharp NP. The population genetics of ploidy change in unicellular fungi. *FEMS Microbiol Rev*. 2021; doi: 10.1093/femsre/fuab006.

21. Naranjo–Ortiz MA, Gabaldón T. Fungal evolution: cellular, genomic and metabolic complexity. *Biol Rev*. 2020; doi: 10.1111/brv.12605.

22. Hill R, Leitch IJ, Gaya E. Targeting Ascomycota genomes: what and how big? *Fungal Biol Rev*. 2021; doi: 10.1016/j.fbr.2021.03.003.

23. Chen G, Rubinstein B, Li R. Whole chromosome aneuploidy: Big mutations drive adaptation by phenotypic leap. *BioEssays*. 2012; doi: 10.1002/bies.201200069.

24. Gerstein AC, Lim H, Berman J, Hickman MA. Ploidy tug-of-war: Evolutionary and genetic environments influence the rate of ploidy drive in a human fungal pathogen. *Evolution (N Y)*. 2017; doi: 10.1111/evo.13205.

25. Peter J, De Chiara M, Friedrich A, Yue J-X, Pflieger D, Bergström A, et al.. Genome evolution across 1,011 *Saccharomyces cerevisiae* isolates. *Nature*. 2018; doi: 10.1038/s41586-018-0030-5.

26. Depotter JR, Seidl MF, Wood TA, Thomma BP. Interspecific hybridization impacts host range and pathogenicity of filamentous microbes. *Curr Opin Microbiol*. 2016; doi:

10.1016/j.mib.2016.04.005.

27. Samarasinghe H, You M, Jenkinson TS, Xu J, James TY. Hybridization facilitates adaptive evolution in two major fungal pathogens. *Genes (Basel)*. 2020; doi: 10.3390/genes11010101.

28. Morrow CA, Fraser JA. Ploidy variation as an adaptive mechanism in human pathogenic fungi. *Semin Cell Dev Biol*. 2013; doi: 10.1016/j.semcdb.2013.01.008.

29. Forche A. Large-scale chromosomal changes and associated fitness consequences in pathogenic fungi. *Curr Fungal Infect Rep*. 2014; doi: 10.1007/s12281-014-0181-2.

30. Gunde-Cimerman N, Zalar P, Hoog S, Plemenitaš A. Hypersaline waters in salterns - natural ecological niches for halophilic black yeasts. *FEMS Microbiol Ecol*. 2000; doi: 10.1111/j.1574-6941.2000.tb00716.x.

31. Plemenitaš A, Lenassi M, Konte T, Kejžar A, Zajc J, Gostinčar C, et al.. Adaptation to high salt concentrations in halotolerant/halophilic fungi: a molecular perspective. *Front Microbiol*. 2014; doi: 10.3389/fmicb.2014.00199.

32. Zalar P, Zupančič J, Gostinčar C, Zajc J, de Hoog GSS, De Leo F, et al.. The extremely halotolerant black yeast *Hortaea werneckii* - a model for intraspecific hybridization in clonal fungi. *IMA Fungus*. 2019; doi: 10.1186/s43008-019-0007-5.

33. Lenassi M, Gostinčar C, Jackman S, Turk M, Sadowski I, Nislow C, et al.. Whole genome duplication and enrichment of metal cation transporters revealed by *de novo* genome sequencing of extremely halotolerant black yeast *Hortaea werneckii*. *PLoS One*. 2013; doi: 10.1371/journal.pone.0071328.

34. Sinha S, Flibotte S, Neira M, Formby S, Plemenitaš A, Gunde-Cimerman N, et al.. Insight into the recent genome duplication of the halophilic yeast *Hortaea werneckii*: Combining an improved genome with gene expression and chromatin structure. *G3-Genes Genomes Genet*. 2017; doi: 10.1534/g3.117.040691.

35. Romeo O, Marchetta A, Giosa D, Giuffrè L, Urzì C, De Leo F. Whole genome sequencing and comparative genome analysis of the halotolerant deep sea black yeast *Hortaea werneckii*. *Life*. 2020; doi: 10.3390/life10100229.

36. Černoša A, Sun X, Gostinčar C, Fang C, Gunde-Cimerman N, Song Z. Virulence traits and population genomics of the black yeast *Aureobasidium melanogenum*. *J Fungi*. 2021; doi: 10.3390/jof7080665.

37. Azua-Bustos A, Gonzalez-Silva C, Salas L, Palma RE, Vicuna R. A novel subaerial *Dunaliella* species growing on cave spiderwebs in the Atacama Desert. *Extremophiles*.

2010; doi: 10.1007/s00792-010-0322-7.
38. Gostinčar C, Turk M, Zajc J, Gunde-Cimerman N. Fifty *Aureobasidium pullulans* genomes reveal a recombining polyextremotolerant generalist. *Environ Microbiol*. 2019; doi: 10.1111/1462-2920.14693.
39. Gostinčar C, Ohm RA, Kogej T, Sonjak S, Turk M, Zajc J, et al.. Genome sequencing of four *Aureobasidium pullulans* varieties: biotechnological potential, stress tolerance, and description of new species. *BMC Genomics*. 2014; doi: 10.1186/1471-2164-15-549.
40. Zhu YO, Sherlock G, Petrov DA. Whole genome analysis of 132 clinical *Saccharomyces cerevisiae* strains reveals extensive ploidy variation. *G3 Genes|Genomes|Genetics*. 2016; doi: 10.1534/g3.116.029397.
41. O'Brien CE, Oliveira-Pacheco J, Ó Cinnéide E, Haase MAB, Hittinger CT, Rogers TR, et al.. Population genomics of the pathogenic yeast *Candida tropicalis* identifies hybrid isolates in environmental samples. Vylkova S, editor. *PLOS Pathog*. 2021; doi: 10.1371/journal.ppat.1009138.
42. Rhodes J, Desjardins CA, Sykes SM, Beale MA, Vanhove M, Sakthikumar S, et al.. Tracing genetic exchange and biogeography of *Cryptococcus neoformans* var. *grubii* at the global population level. *Genetics*. 2017; doi: 10.1534/genetics.117.203836.
43. Gabaldón T. Hybridization and the origin of new yeast lineages. *FEMS Yeast Res*. 2020; doi: 10.1093/femsyr/foaa040.
44. Pryszcz LP, Németh T, Gácser A, Gabaldón T. Genome comparison of *Candida orthopsilosis* clinical strains reveals the existence of hybrids between two distinct subspecies. *Genome Biol Evol*. 2014; doi: 10.1093/gbe/evu082.
45. Pryszcz LP, Németh T, Saus E, Ksiezopolska E, Hegedűsová E, Nosek J, et al.. The genomic aftermath of hybridization in the opportunistic pathogen *Candida metapsilosis*. Zhang J, editor. *PLOS Genet*. 2015; doi: 10.1371/journal.pgen.1005626.
46. Gostinčar C, Stajich JE, Kejžar A, Sinha S, Nislow C, Lenassi M, et al.. Seven years at high salinity — experimental evolution of the extremely halotolerant black yeast *Hortaea werneckii*. *J Fungi*. 2021; doi: 10.3390/jof7090723.
47. Boekhout T, Aime MC, Begerow D, Gabaldón T, Heitman J, Kemler M, et al.. The evolving species concepts used for yeasts: from phenotypes and genomes to speciation networks. *Fungal Divers*. 2021; doi: 10.1007/s13225-021-00475-9.
48. Mixão V, Gabaldón T. Hybridization and emergence of virulence in opportunistic

human yeast pathogens. *Yeast*. 2018; doi: 10.1002/yea.3242.

49. Strom NB, Bushley KE. Two genomes are better than one: history, genetics, and biotechnological applications of fungal heterokaryons. *Fungal Biol Biotechnol*. 2016; doi: 10.1186/s40694-016-0022-x.

50. Mitchison-Field LMY, Vargas-Muñiz JM, Stormo BM, Vogt EJD, Van Dierdonck S, Pelletier JF, et al.. Unconventional cell division cycles from marine-derived yeasts. *Curr Biol*. 2019; doi: 10.1016/j.cub.2019.08.050.

51. Saupe SJ. Molecular genetics of heterokaryon incompatibility in filamentous Ascomycetes. *Microbiol Mol Biol Rev*. 2000; doi: 10.1128/MMBR.64.3.489-502.2000.

52. Gladieux P, Ropars J, Badouin H, Branca A, Aguileta G, Vienne DM, et al.. Fungal evolutionary genomics provides insight into the mechanisms of adaptive divergence in eukaryotes. *Mol Ecol*. 2014; doi: 10.1111/mec.12631.

53. Berman J, Hadany L. Does stress induce (para)sex? Implications for *Candida albicans* evolution. *Trends Genet*. 2012; doi: 10.1016/j.tig.2012.01.004.

54. Sun X, Gostinčar C, Fang C, Zajc J, Hou Y, Song Z, et al.. Genomic Evidence of Recombination in the Basidiomycete *Wallemia mellicola*. *Genes (Basel)*. 2019; doi: 10.3390/genes10060427.

55. Drenth A, McTaggart AR, Wingfield BD. Fungal clones win the battle, but recombination wins the war. *IMA Fungus*. 2019; doi: 10.1186/s43008-019-0020-8.

56. Gostinčar C, Zajc J, Lenassi M, Plemenitaš A, de Hoog S, Al-Hatmi AMS, et al.. Fungi between extremotolerance and opportunistic pathogenicity on humans. *Fungal Divers*. 2018; doi: 10.1007/s13225-018-0414-8.

57. Zajc J, Gostinčar C, Černoša A, Gunde-Cimerman N. Stress-tolerant yeasts: Opportunistic pathogenicity versus biocontrol potential. *Genes (Basel)*. 2019; doi: 10.3390/genes10010042.

58. Gostinčar C. Towards genomic criteria for delineating fungal species. *J Fungi*. 2020; doi: 10.3390/jof6040246.

59. Fogelqvist J, Tzelepis G, Bejai S, Ilbäck J, Schwelm A, Dixelius C. Analysis of the hybrid genomes of two field isolates of the soil-borne fungal species *Verticillium longisporum*. *BMC Genomics*. 2018; doi: 10.1186/s12864-017-4407-x.

60. Michelotti LA, Sun S, Heitman J, James TY. Clonal evolution in serially passaged *Cryptococcus neoformans* × *deneoformans* hybrids reveals a heterogenous landscape of genomic change. Stajich J, editor. *Genetics*. 2022; doi: 10.1093/genetics/iyab142.

61. Steenwyk JL, Lind AL, Ries LNA, dos Reis TF, Silva LP, Almeida F, et al..

Pathogenic allodiploid hybrids of *Aspergillus* fungi. *Curr Biol*. 2020; doi: 10.1016/j.cub.2020.04.071.

62. Fang C, Zhong H, Lin Y, Chen B, Han M, Ren H, et al.. Assessment of the cPAS-based BGISEQ-500 platform for metagenomic sequencing. *Gigascience*. 2018; doi: 10.1093/gigascience/gix133.

63. Huang S, Kang M, Xu A. HaploMerger2: rebuilding both haploid sub-assemblies from high-heterozygosity diploid genome assembly. Berger B, editor. *Bioinformatics*. 2017; doi: 10.1093/bioinformatics/btx220.

64. Li H, Handsaker B, Wysoker A, Fennell T, Ruan J, Homer N, et al.. The Sequence Alignment/Map format and SAMtools. *Bioinformatics*. 2009; doi: 10.1093/bioinformatics/btp352.

65. Alkan C, Coe BP, Eichler EE. GATK toolkit. *Nat Rev Genet*. 2011; doi: 10.1038/nrg2958.

66. R Development Core Team. R: A language and environment for statistical computing. Vienna, Austria: R Foundation for Statistical Computing;

67. Jombart T, Ahmed I. adegenet 1.3-1: new tools for the analysis of genome-wide SNP data. *Bioinformatics*. 2011; doi: 10.1093/bioinformatics/btr521.

68. Schliep K, Potts AJ, Morrison DA, Grimm GW. Intertwining phylogenetic trees and networks. *Methods Ecol Evol*. 2017; doi: 10.1111/2041-210X.12760.

69. Kamvar ZN, Brooks JC, Grünwald NJ. Novel R tools for analysis of genome-wide population genetic data with emphasis on clonality. *Front Genet*. 2015; doi: 10.3389/fgene.2015.00208.

70. Danecek P, Auton A, Abecasis G, Albers CA, Banks E, DePristo MA, et al.. The variant call format and VCFtools. *Bioinformatics*. 2011; doi: 10.1093/bioinformatics/btr330.

71. Wickham H. ggplot2: Elegant graphics for data analysis. Springer-Verlag New York;

72. Peng Y, Leung HCM, Yiu SM, Chin FYL. IDBA-UD: A *de novo* assembler for single-cell and metagenomic sequencing data with highly uneven depth. *Bioinformatics*. 2012; doi: 10.1093/bioinformatics/bts174.

73. Stanke M, Morgenstern B. AUGUSTUS: a web server for gene prediction in eukaryotes that allows user-defined constraints. *Nucleic Acids Res*. 2005; doi: 10.1093/nar/gki458.

74. Simão FA, Waterhouse RM, Ioannidis P, Kriventseva E V., Zdobnov EM. BUSCO:

Assessing genome assembly and annotation completeness with single-copy orthologs. *Bioinformatics*. 2015; doi: 10.1093/bioinformatics/btv351.

75. Kriventseva E V., Tegenfeldt F, Petty TJ, Waterhouse RM, Simão FA, Pozdnyakov IA, et al.. OrthoDB v8: update of the hierarchical catalog of orthologs and the underlying free software. *Nucleic Acids Res*. 2015; doi: 10.1093/nar/gku1220.

76. Geib SM, Hall B, Derego T, Bremer FT, Cannoles K, Sim SB. Genome Annotation Generator: a simple tool for generating and correcting WGS annotation tables for NCBI submission. *Gigascience*. 2018; doi: 10.1093/gigascience/giy018.

77. Minkin I, Medvedev P. Scalable multiple whole-genome alignment and locally collinear block construction with SibeliaZ. *Nat Commun*. 2020; doi: 10.1038/s41467-020-19777-8.

78. Talavera G, Castresana J. Improvement of phylogenies after removing divergent and ambiguously aligned blocks from protein sequence alignments. *Syst Biol*. 2007; doi: 10.1080/10635150701472164.

79. Minh BQ, Schmidt HA, Chernomor O, Schrempf D, Woodhams MD, von Haeseler A, et al.. IQ-TREE 2: New models and efficient methods for phylogenetic inference in the genomic era. Teeling E, editor. *Mol Biol Evol*. 2020; doi: 10.1093/molbev/msaa015.

80. Huson DH, Bryant D. Application of phylogenetic networks in evolutionary studies. *Mol Biol Evol*. 2006; doi: 10.1093/molbev/msj030.

81. Revell LJ. phytools: an R package for phylogenetic comparative biology (and other things). *Methods Ecol Evol*. 2012; doi: 10.1111/j.2041-210X.2011.00169.x.

82. Mistry J, Chuguransky S, Williams L, Qureshi M, Salazar GA, Sonnhammer ELL, et al.. Pfam: The protein families database in 2021. *Nucleic Acids Res*. 2021; doi: 10.1093/nar/gkaa913.

83. Katoh K, Standley DM. MAFFT multiple sequence alignment software version 7: improvements in performance and usability. *Mol Biol Evol*. 2013; doi: 10.1093/molbev/mst010.

84. Altschul S. Gapped BLAST and PSI-BLAST: a new generation of protein database search programs. *Nucleic Acids Res*. 1997; doi: 10.1093/nar/25.17.3389.

Table 1. *Hortaea werneckii* strains analysed in this study.

Culture collection strain number	Present study number*	Isolation habitat	Sampling site location
EXF-9	1	brine	Ebre Delta salterns, Spain
EXF-12	2	brine	Santa Pola salterns, Spain
EXF-15	3	brine	Santa Pola salterns, Spain
EXF-20	4	brine	Santa Pola salterns, Spain
EXF-152	5	brine	Sečovlje salterns, Slovenia
EXF-153, EXF-2781	6	brine	Sečovlje salterns, Slovenia
EXF-154	7	brine	Sečovlje salterns, Slovenia
EXF-156, CBS 116.90	8	eye infection of aquarium *Spondyliosoma cantharus*	unknown
EXF-157, CBS 115.90	9	kidney of *Bufo granulosus*	Brazil
EXF-161, EXF-2689, CBS 706.76	10	leaf of *Rhizophora mangle*	Senegal
EXF-166, CBS 100496	11	sea water-sprayed marble	Delos, Greece
EXF-177, CBS 705.76	12	*tinea nigra*	France
EXF-241	13	brine	Sečovlje salterns, Slovenia
EXF-269, EXF-108	14	brine	Santa Pola salterns, Spain
EXF-291	15	brine	Sečovlje salterns, Slovenia
EXF-561	16	brine	Namibia, salterns at the Atlantic coast
EXF-2515	17	brine	salterns, Puerto Rico
EXF-2516	18	brine	salterns, Puerto Rico
EXF-2683, CBS 117.90	19	salted fish, *Osteoglossum bicirrhosum*	Brazil
EXF-2685	20	brine	Sečovlje salterns, Slovenia
EXF-2783	21	brine	Sečovlje salterns, Slovenia
EXF-2785	22	brine	Sečovlje salterns, Slovenia
EXF-3845	23	brine	Candelaria salterns, Puerto Rico
EXF-3846	24	brine	Candelaria salterns, Puerto Rico
EXF-4716	25	brine bait	Sečovlje salterns, Slovenia
EXF-6274	26	brine	Sečovlje salterns, Slovenia
EXF-6652	27	spider web in a cave close to the ocean	Atacama, Chile
EXF-6663	28	spider web in a cave close to the ocean	Atacama, Chile
EXF-8170	29	brine	Sečovlje salterns, Slovenia
EXF-8422	30	biofilm from cheese factory brine	Celje, Slovenia
EXF-10304	31	brine	Sečovlje salterns, Slovenia
EXF-10508	32	sea water, depth 25 m	Italy
EXF-10509	33	sea water, depth 200 m	Italy
EXF-10510	34	sea water, depth 94 m	Italy
EXF-10511	35	sea water, depth 25 m	Italy
EXF-10512	36	sea water, depth 25 m	Italy
EXF-10816	37	bittern after halite precipitation	Sečovlje salterns, Slovenia
EXF-10819	38	bittern after halite precipitation	Sečovlje salterns, Slovenia
EXF-10820	39	bittern after halite precipitation	Sečovlje salterns, Slovenia
EXF-10843	40	brine	Sečovlje salterns, Slovenia
EXF-10904	41	bittern after halite precipitation	Sečovlje salterns, Slovenia
EXF-10907	42	bittern after halite precipitation	Sečovlje salterns, Slovenia
EXF-10919	43	bittern after halite precipitation	Sečovlje salterns, Slovenia
EXF-10958	44	bittern after halite precipitation	Sečovlje salterns, Slovenia
EXF-10974	45	brine	Sečovlje salterns, Slovenia
EXF-11540	46	sand in a cave close to the ocean	Atacama, Chile
EXF-11650	47	sand in a cave close to the ocean	Atacama, Chile
EXF-11651	48	sand in a cave close to the ocean	Atacama, Chile
EXF-12619	49	coral or deep sea	China
EXF-12620	50	coral or deep sea	China
EXF-14591, CMF-020	51	plankton tow	Vineyard Sound, USA
EXF-14592, AMF 061	52	plankton tow	Vineyard Sound, USA
EXF-225**	53	malt extract medium, 25% NaCl (w/v)	long-term experimental evolution
EXF-14590, MSW 12-1B	54	marine	List on Sylt, Germany

EXF-2000	A***	brine	Sečovlje salterns, Slovenia
EXF-120	B	brine	Santa Pola saltpans, Spain
EXF-562	C	Soil on the sea coast	Namibia
EXF-2788	D	brine	Sečovlje salterns, Slovenia
EXF-171	E	keratomycosis	Brazil
EXF-2682	F	*trichomycosis nigra*	Italy
EXF-10513	G	deep sea water	Italy
EXF-151	H	*tinea nigra*	Portugal
EXF-6651	I	spider web in a cave close to the ocean	Atacama, Chile
EXF-6669	J	spider web in a cave close to the ocean	Atacama, Chile
EXF-6654	K	spider web in a cave close to the ocean	Atacama, Chile
EXF-6656	L	rock wall in a cave close to the ocean	Atacama, Chile

* strains 1-54 were sequenced in this study; strains A-L were sequenced and named by Gostinčar et al. [1]

** strain EXF-225 after 15 years of repeated subcultivation at 25% NaCl (w/v) 5

*** reference *H. werneckii* genome [34]; naming of strains A-L corresponds to names in Gostinčar et al. [1]

Table 2. *Aureobasidium melanogenum* strains analysed in this study.

Culture collection strain number	Present study number*	Isolation habitat	Sampling site location
EXF-924	1	ponds on sea ice	Svalbard, Norway
EXF-926	2	surface glacial ice	Svalbard, Norway
EXF-3233	3	deep sea (4500 m b.s.l.)	Japan
EXF-3371	4	soil	Thailand
EXF-3378	5	public fountain	Thailand
EXF-3397	6	endoperitoneal fluid	Greece
EXF-4450	8	Iskra factory	Slovenia
EXF-5590	9	dishwasher rubber seal	Slovenia
EXF-6171	10	glacial ice	Argentina
EXF-7932	11	metal drain on the kitchen sink	Sweden
EXF-7946	12	kitchen metal holder for washed dishes	Sweden
EXF-8016	13	bathroom faucet and sink contact	Sweden
EXF-8022	14	refrigerator inner surface	Sweden
EXF-8044	15	kitchen metal holder for washed dishes	Sweden
EXF-8258	16	well water	Slovenia
EXF-9877	17	tap water	Slovenia
EXF-11403	18	refrigerator inner surface	Sweden
EXF-8492	19	well water	Slovenia
EXF-8678	20	well water	Slovenia
EXF-8689	21	well water	Slovenia
EXF-8695	22	well water	Slovenia
EXF-8702	23	well water	Slovenia
EXF-8986	24	fango mud from Sečovlje salterns	Slovenia
EXF-9262	25	rubber on kitchen drain	Slovenia
EXF-9470	26	kitchen counter above dishwasher	Slovenia
EXF-9272	27	kitchen strainer basket	Slovenia
EXF-9298	28	plastic mesh on kitchen drain	Slovenia
EXF-9304	29	kitchen strainer basket	Slovenia
EXF-9313	30	kitchen sink	Slovenia
EXF-9454	31	tap water	Slovenia
EXF-9484	32	kitchen counter above dishwasher	Slovenia
EXF-9887	33	tap water	Slovenia
EXF-9516	34	kitchen sink drain	Slovenia
EXF-9539	35	kitchen strainer basket	Slovenia
EXF-9540	36	dishwasher door	Slovenia
EXF-10064	37	tap water	Slovenia
EXF-11060	38	ceiling surface	Slovenia
EXF-9875	39	tap water	Slovenia
EXF-9906	40	*Arthrocnemum* sp. plant surface from Sečovlje saltern	Slovenia
EXF-9911	41	kitchen sink drain	Slovenia
EXF-9937	42	kitchen sink drain	Slovenia
EXF-10061	43	tap water	Slovenia
EXF-10062	44	tap water	Slovenia
EXF-10066	45	tap water	Slovenia
EXF-10333	46	tap water	Slovenia
EXF-10372	47	air in the National Gallery restoration centre	Slovenia
EXF-10726	48	integument of a male alate ant of *Atta sexdens rubropilosa*	Brazil
EXF-11028	49	water from the aquarium with *Proteus anguinus*	Slovenia

* same numbering as in Černoša et al. [36]

Table 3. Statistics for the *H. werneckii* genomes sequenced in this study (strains 1-54).

	haploid strains			diploid strains			tetraploid strain
	average	median	SD	average	median	SD	/
Coverage	730	619	464	469	485	177	276
Genome assembly size (Mbp)	26.52	26.19	1.47	49.30	49.22	1.74	94.67
Number of contigs	796	638	421	6885	3806	4457	30312
Contig N50 (kbp)	136	138	28	22	26	14	5
GC content	53.22%	53.33%	0.33%	53.40%	53.40%	0.19%	53.40%
CDS total length (Mbp)	14.56	14.39	0.80	27.02	27.87	1.45	49.02
CDS total length (% of genome)	54.94%	55.27%	1.67%	54.80%	55.59%	2.15%	51.78%
Gene models (n)	9519	9344	665	20417	19240	1709	46596
Exons per gene (average)	2.34	2.34	0.06	2.10	2.20	0.14	1.87
Intron average length (bp)	93.17	93.00	2.53	94.11	94.00	4.73	92.00
Complete BUSCOs *	95.99%	96.00%	0.34%	86.86%	93.40%	10.09%	89.60%
Complete and single-copy BUSCOs	95.83%	95.90%	0.35%	21.33%	16.30%	10.50%	33.20%
Complete and duplicated BUSCOs	0.16%	0.20%	0.06%	65.53%	77.30%	19.20%	56.40%
Fragmented BUSCOs	0.82%	0.75%	0.14%	7.05%	3.20%	5.87%	5.10%
Missing BUSCOs	3.19%	3.10%	0.30%	6.09%	3.30%	4.24%	5.30%
Total SNP density (SNPs per total genome size)	4.04%	4.54%	1.11%	3.44%	3.56%	1.12%	/
Heterozygous SNP density (SNPs per total genome size)	0.01%	0.01%	0.01%	2.46%	2.58%	0.74%	/

* BUSCOs, Benchmarking Universal Single-Copy Orthologues.

Figure legends

Figure 1. Single-nucleotide polymorphism (SNP) diversity of *Hortaea werneckii* (**A, B**) and *Aureobasidium melanogenum* (**C, D**). (**A, C**) Phylogenetic networks reconstructed with a Neighbor-Net algorithm from a dissimilarity distance matrix calculated from SNP data. (**B, D**) Principal component analysis of SNPs. The genomes are represented by circles.

Figure 2. Linkage disequilibrium (LD) decay in *Hortaea werneckii* (**A**) and *Aureobasidium melanogenum* (**B**). Squared correlation coefficient (r^2) was calculated for all pairs of non-singleton biallelic loci within the distance of 10 kbp or less and plotted as a function of the distance between the loci (blue line). The maximum observed value and its half value are marked with red horizontal dashed lines. A generalized additive model curve was fitted to the data (black line).

Figure 3. Phylogenies of 50 longest alignable genomic regions of *Hortaea werneckii* (**A, C**) and *Aureobasidium melanogenum* (**B, D**). The alignable regions were extracted from the genomes and aligned with SibeliaZ, optimized with Gblocks, manually inspected and used for phylogeny reconstruction with IQ-TREE and standard model selection. (**A, B**) Overlay of 50 phylogenies for each species. Numbers on leaf nodes represent genomes, different sequences from the same genomes are distinguished with letters added to the genome numbers. (**C, D**) Consensus supernetworks calculated from 50 phylogenies for each species in SplitsTree.

Figure 4. Hypothesis of the genome evolution and hybridization in *Hortaea werneckii* (**A**) and *Aureobasidium melanogenum* (**B**). The hypothesis is based on the majority consensus phylogeny of 50 longest alignable regions per species. Each coloured line in the central tree represents a haploid genome. The distances between the nodes of the tree correspond to the distances in an ultrametric majority consensus phylogeny. Haploid genomes are represented by a single coloured line in the outermost edge of the tree, diploid genomes are represented by a double coloured line, the only tetraploid genome is represented by four coloured lines. Around the tree, coloured symbols mark the continent (inner circle) and habitat (outer circle) from which the strains have been

originally isolated. Black lines and numbers in the outermost circle mark the genome/ strain groups presumably originating from the same hybridization event.

Figure 5. Aneuploid regions in *Hortaea werneckii* (**A**) and *Aureobasidium melanogenum* (**B**) genomes. Per-nucleotide sequencing depth of regions corresponding to 50 and 35 longest contigs of *H. werneckii* and *A. melanogenum* were converted into proportion of the median sequencing depth of each individual genome. Circles represent an average of this depth in 30 kbp windows. The central horizontal line marks the median sequencing depth of the genome. Upper and lower horizontal lines mark the expected depth for haploid and triploid regions in an otherwise diploid genome. Genomes with at least one putatively aneuploid region are plotted in colour. Other genomes are plotted in light grey.

Supplementary Information

Supplemental Table S1. Statistics of *H. werneckii* genomes sequenced in this study.

Supplemental Table S2. Putative HET and HET-C proteins in different strains of *H. werneckii* and *A. melanogenum*.

Supplemental Figure S1. Aneuploid regions in *Hortaea werneckii* genomes. Per-nucleotide sequencing depth of regions corresponding to 50 longest contigs of *H. werneckii* and *A. melanogenum* were converted into proportion of the median sequencing depth of each individual genome and plotted in 50 kbp rolling median windows (black line). Upper and lower horizontal lines mark the expected depth for haploid and triploid regions in an otherwise diploid genome. Putatively aneuploid region of increased ploidy are marked with red rectangles.

Supplemental Figure S2. Aneuploid regions in *Aureobasidium melanogenum* genomes. Per-nucleotide sequencing depth of regions corresponding to 35 longest contigs of *A. melanogenum* were converted into proportion of the median sequencing depth of each individual genome and plotted in 50 kbp rolling median windows (black line). Upper and lower horizontal lines mark the expected depth for haploid and triploid regions in an otherwise diploid genome. Putatively aneuploid region of increased ploidy are marked with red rectangles.

Supplemental Figure S3. Heterozygosity in diploid *Hortaea werneckii* and *Aureobasidium melanogenum* genomes. Levels of heterozygosity (black lines) and sequencing depth (purple lines) were expressed as proportions of median heterozygosity and sequencing depth of each individual genome. The values were plotted in 25 kbp windows across regions corresponding to 50 and 35 longest contigs of *H. werneckii* and *A. melanogenum*, respectively. Diploid regions (i.e. with sequencing depth similar to the rest of the diploid genome) with extensive loss of heterozygosity are marked with red rectangles.

Supplemental Figure S4. Putative mating type loci of *Hortaea werneckii*. Phylogenies and presence/absence visualisation of MAT1-1 and MAT1-2 homologues, as well as an annotated alignment of the whole putative mating locus and its flanking regions.

Supplemental Figure S5. Putative mating type loci of *Aureobasidium melanogenum*. Phylogenies and presence/absence visualisation of MAT1-1 and MAT1-2 homologues, as well as an annotated alignment of the whole putative mating locus and its flanking regions.

Figure 1

Click here to access/download;Figure;Fig1.png

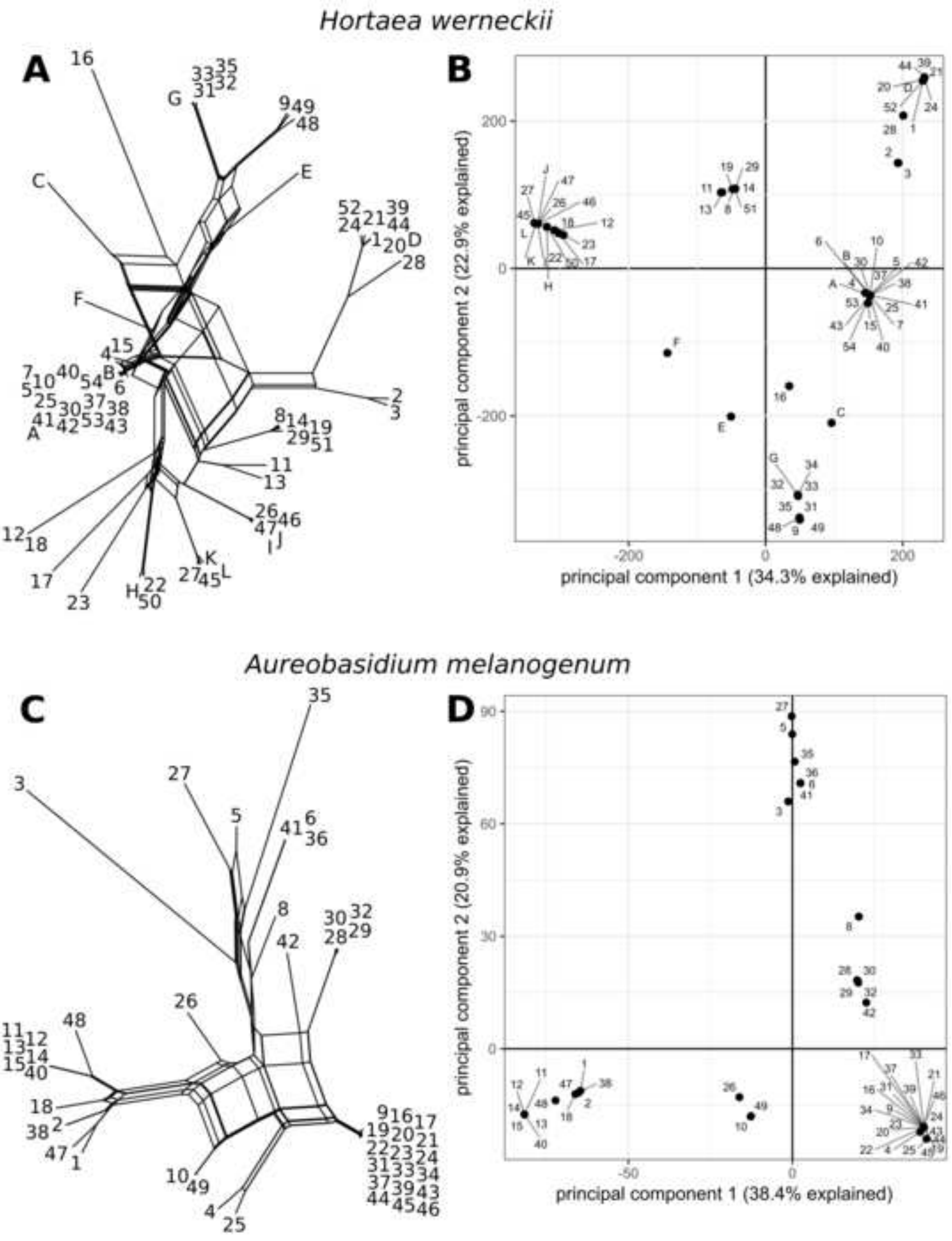

Figure 2

Click here to access/download;Figure;Fig2.png

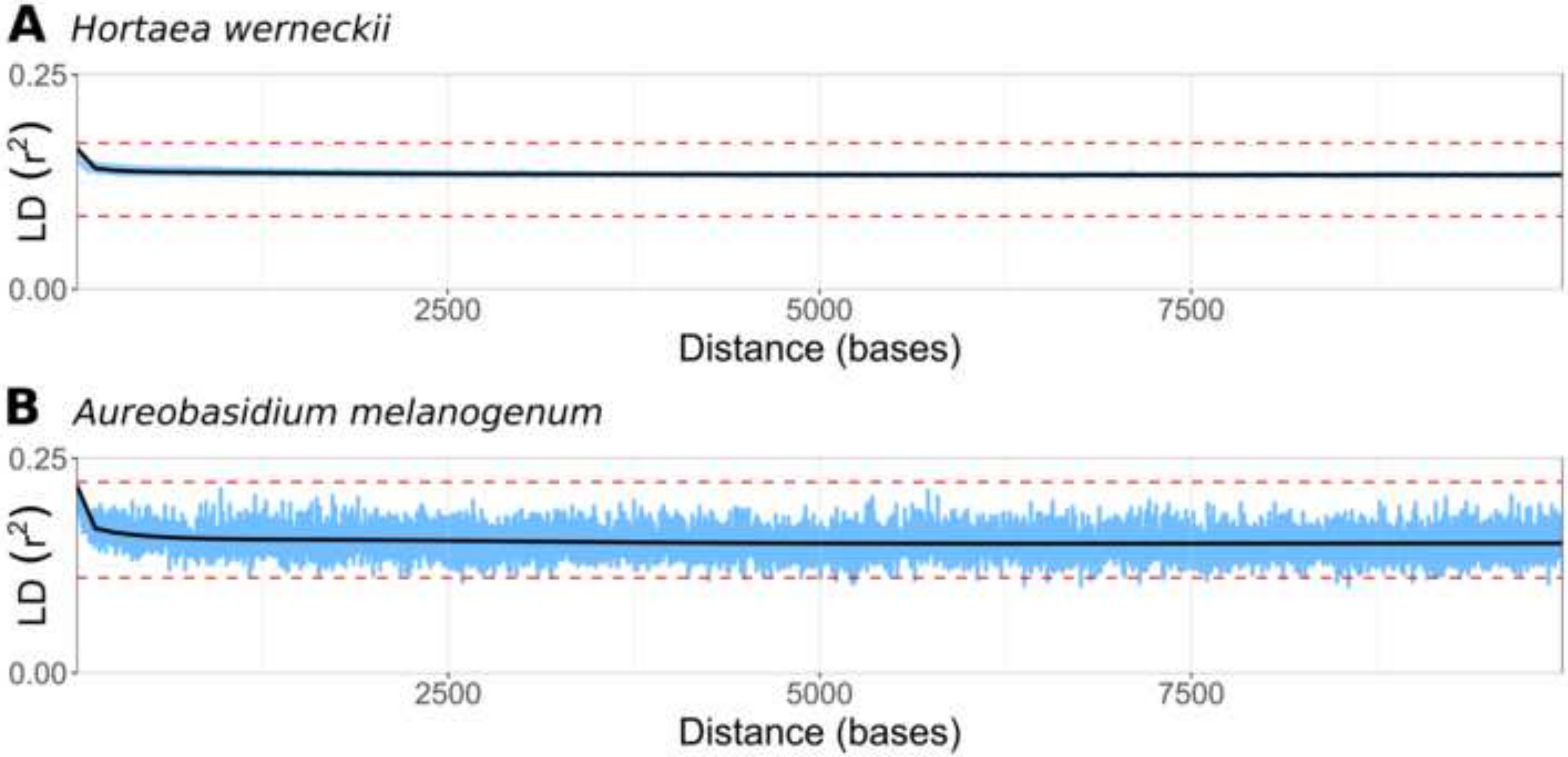

Figure 3 Click here to access/download;Figure;Fig3.png

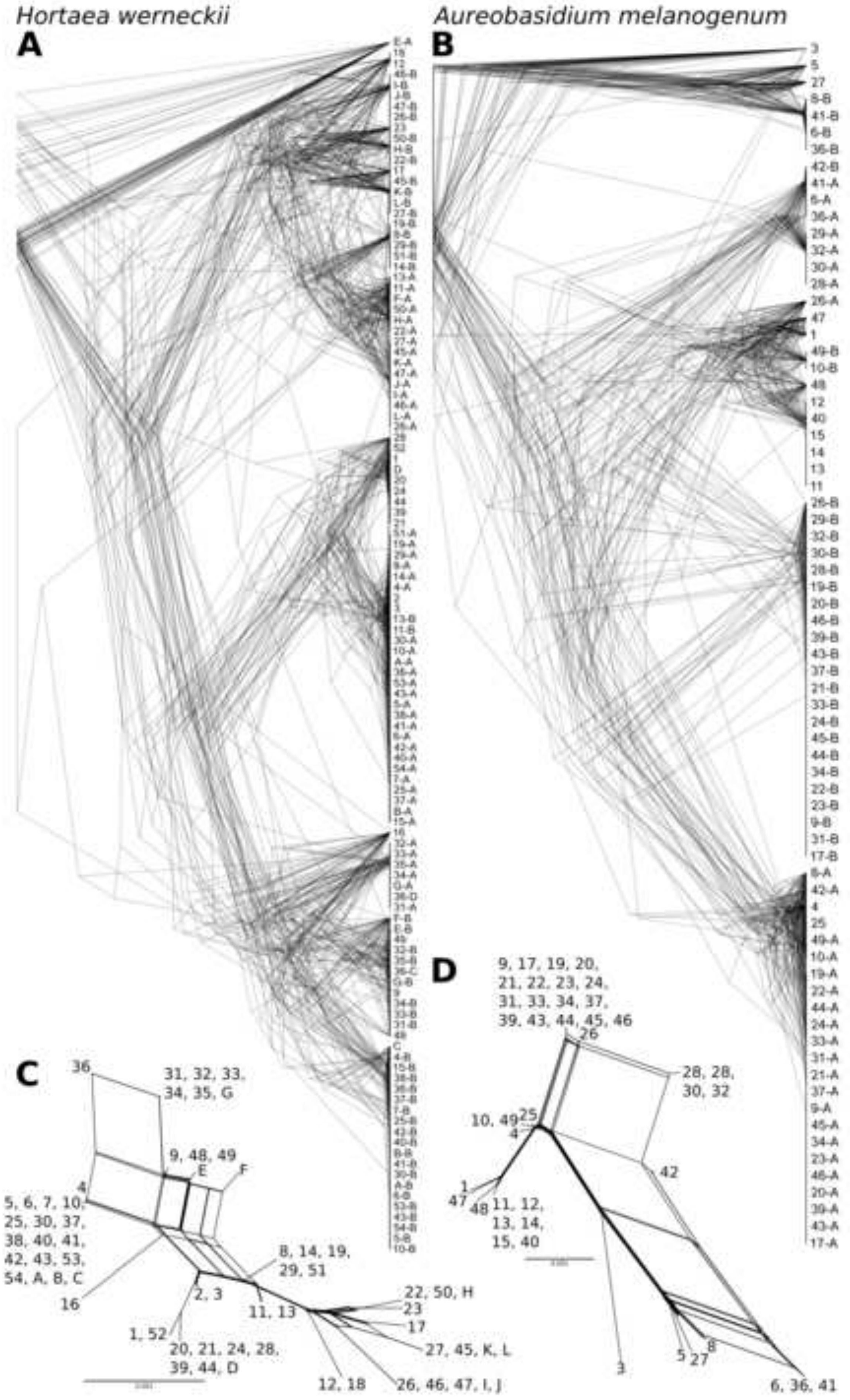

Figure 4 Click here to access/download;Figure;Fig4.png

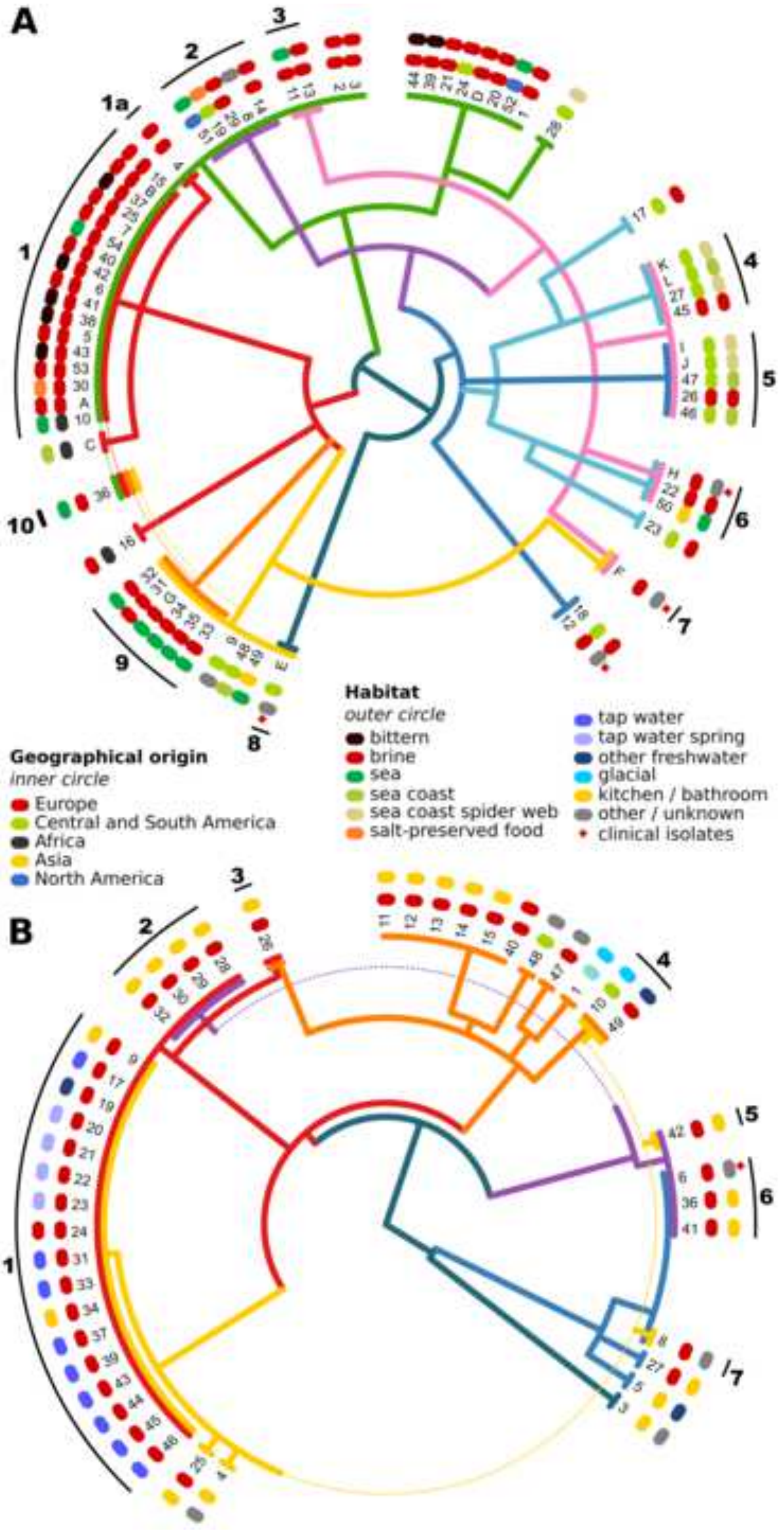

Figure 5

Click here to access/download;Figure;Fig5.png

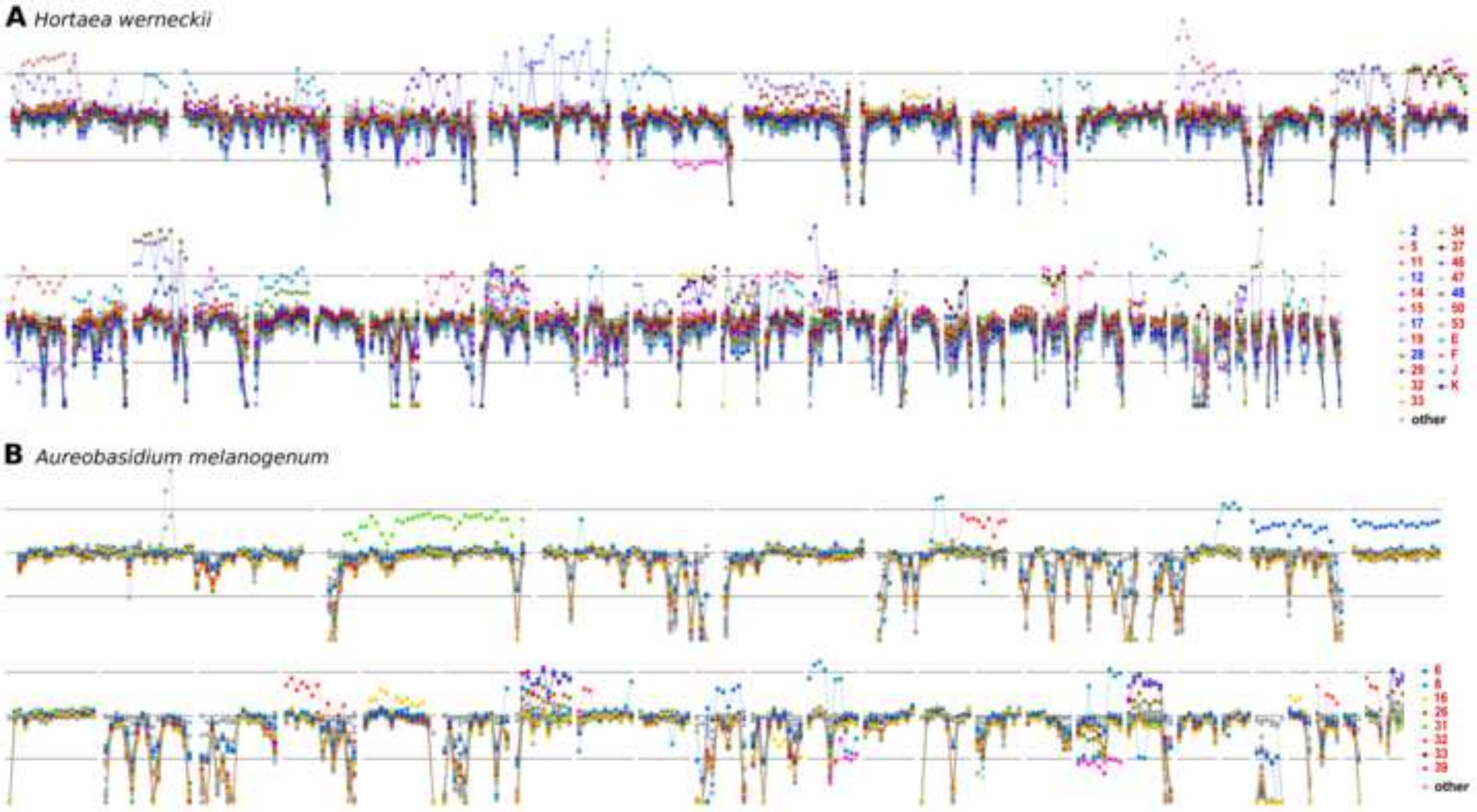

Supplemental Table S1

Click here to access/download
Supplementary Material
SupplementalTableS1.xlsx

Supplemental Table S2

Click here to access/download
Supplementary Material
SupplementalTableS2.xlsx

Supplemental Figure S1

Click here to access/download
Supplementary Material
FigS1.png

Supplemental Figure S2

Click here to access/download
Supplementary Material
FigS2.png

Supplemental Figure S3

Click here to access/download
Supplementary Material
FigS3.png

Supplemental Figure S4

Click here to access/download
Supplementary Material
FigS4.pdf

Supplemental Figure S5

Click here to access/download
Supplementary Material
FigS5.pdf
