# Supplementary material for: Clonality, inbreeding, and hybridization in two extremotolerant black yeasts
Source: Gigascience. 2022 Oct 6;11:giac095. doi: 10.1093/gigascience/giac095 (PMC9535773; doi:10.1093/gigascience/giac095)
Supplement: giac095_Supplemental_Files [file giac095_supplemental_files.zip › FigS4.rev1.pdf]

MAT1-1

Phylogeny of MAT1-1 regions in genomes of *H. werneckii* (left) and the presence/absence of putative MAT1-1 genes in the sequenced strains (right; hybridisation tree as in the main Fig. 4). MAT genes can be missing due to (i) absence in the genome <u>or</u> (ii) incomplete assembly of the MAT locus during genome assembly. Alignment of the entire putative mating loci is below.

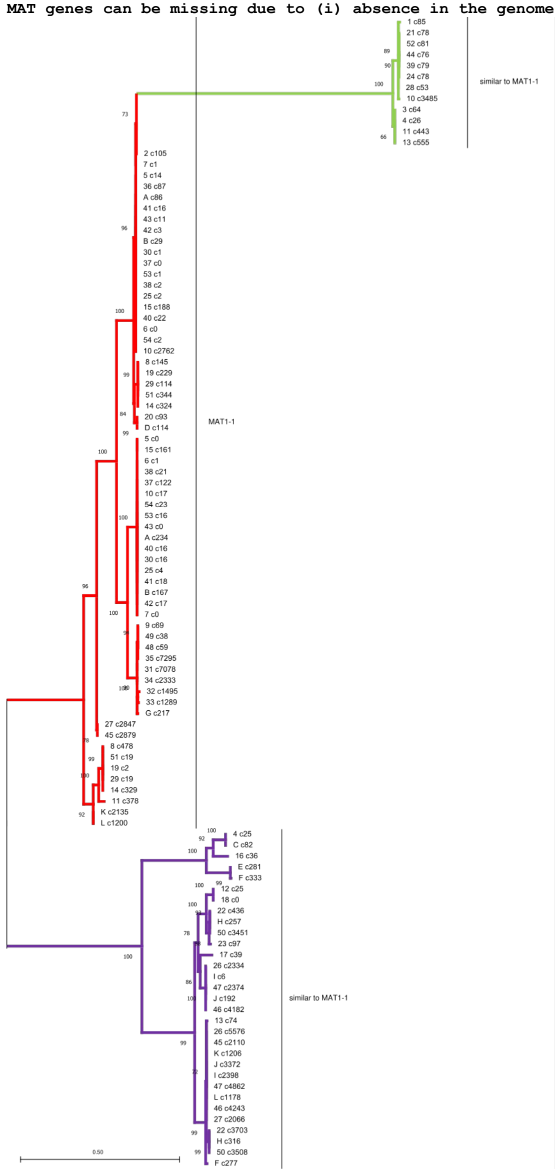

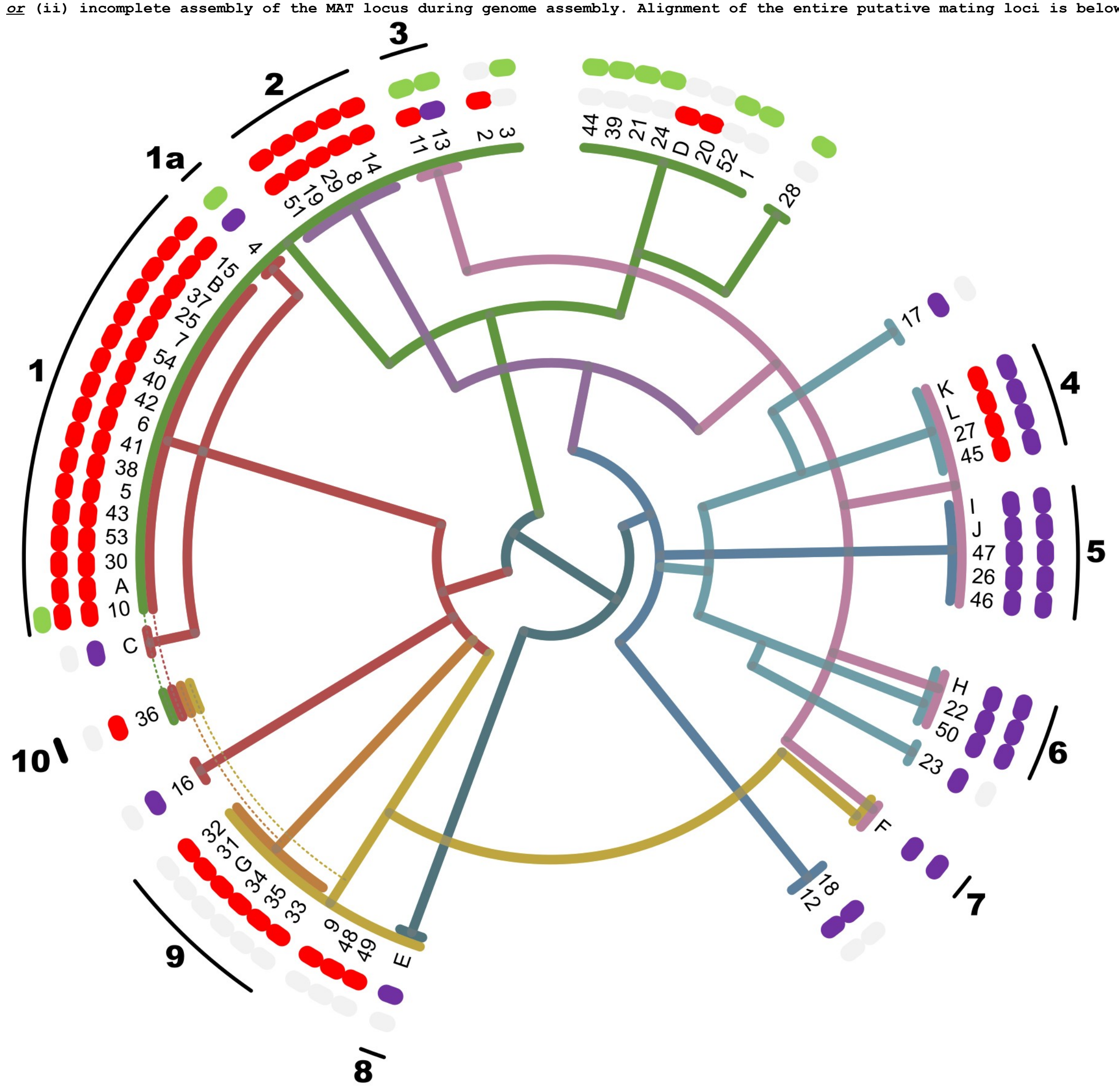

Depth of seuencing of MAT regions. The location of MAT genes are marked with yellow (MAT1-1) and red (MAT1-2). Note that gaps in coverage can be caused by the absence of the region or the high divergence of the region from the reference MAT region (such as in the case of green and purple MAT1-1 clusters shown in the phylogeny on the left).

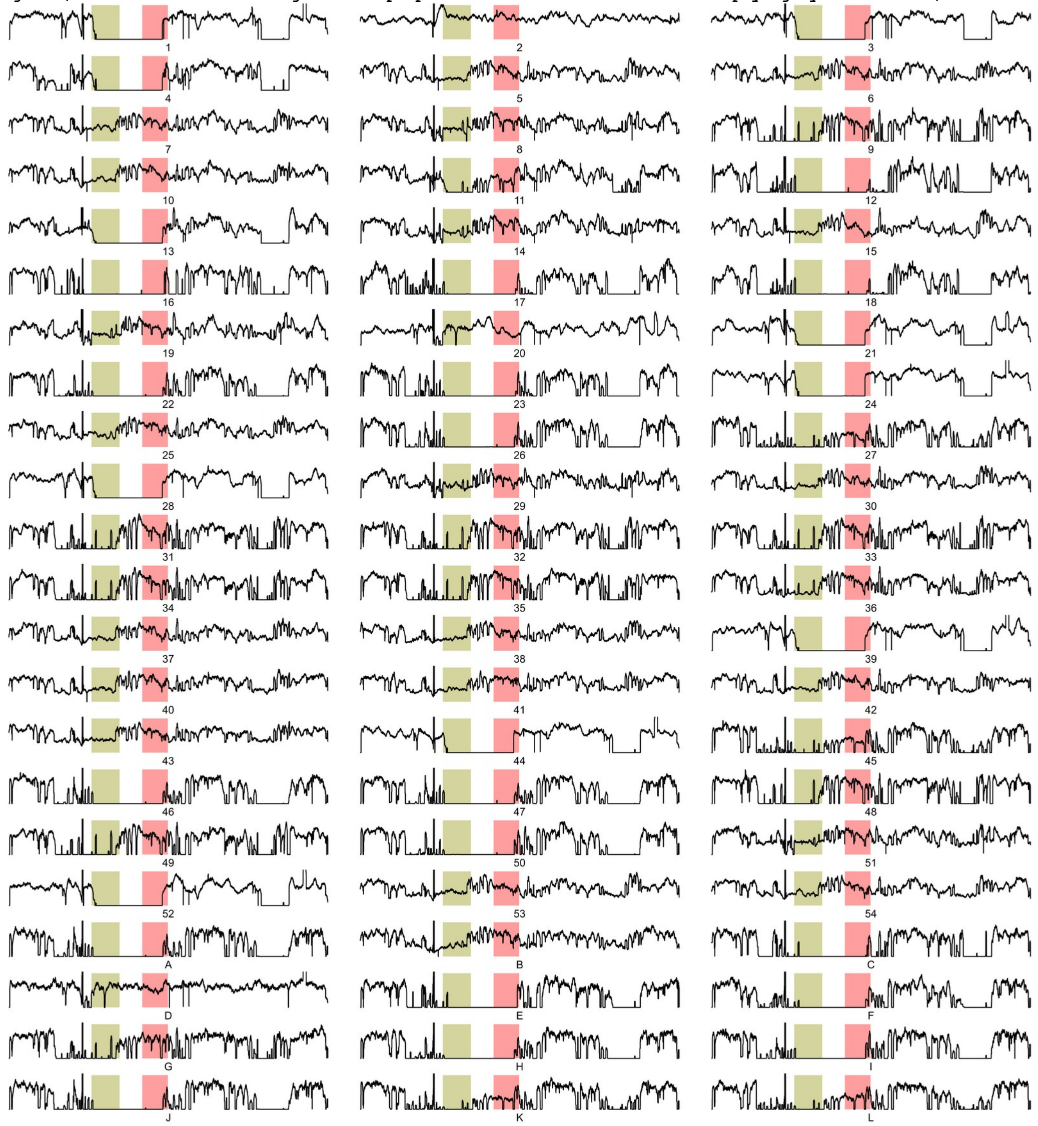

MAT1-2

Phylogeny of MAT1-2 regions in genomes of *H. werneckii* (left) and the presence/absence of putative MAT1-2 genes in the sequenced strains (right; hybridisation tree as in the main Fig. 4). MAT genes can be missing due to (i) absence in the genome <u>or</u> (ii) incomplete assembly of the MAT locus during genome assembly. Alignment of the entire putative mating loci is below.

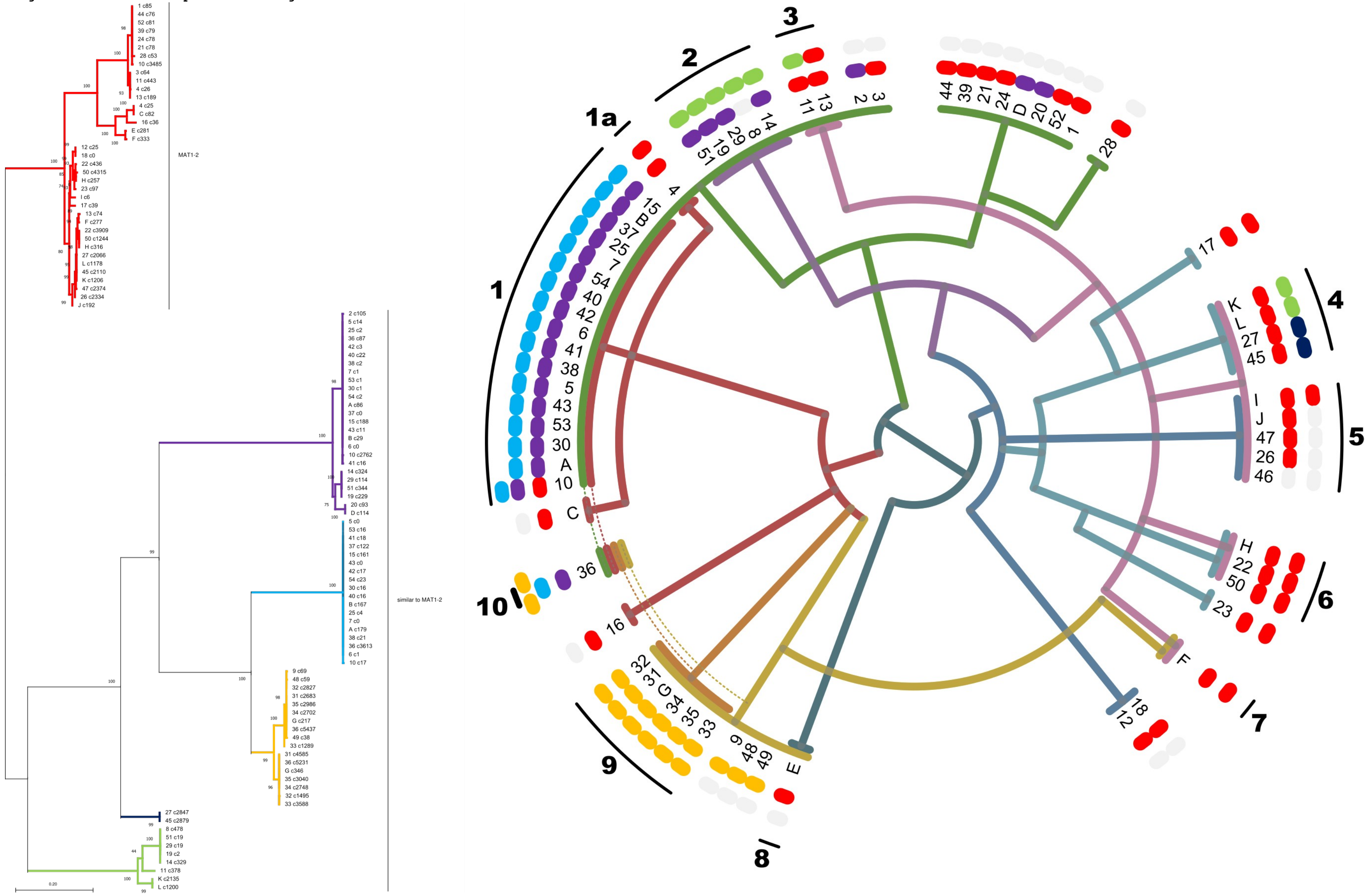

```
1_c85     ACGAGGGCAGTTCACGCA-GCGGCTACTTCAGGCGCCCCACGAAATGAGCAGGAGTCAGCGGGTTCATCTGTTGGTGGACGAAGCAGGACTCCATCCAAGATGAGGCTCACTACTTGGTACGTGAAGCCGATGCTGATCCGCAAAGCCACTC [illegible]
2_c105    ACGTGGGCAGTACTCGTACGCAGCTGCGCCAAGCACTCCGCGAAATGAGAAGGAGGCAGCGGGTTCATCTGTTGGTGGACGAAGCAGGACTCCATCCAAGATGAGGCTCACTACTTGGTACGTGAAGCCAGTGCTGATCCATAAAGCCACTC [illegible]
3_c64     ACGTGGGCAGTACTCGTACGCAGCTGCGCCAAGCACTCCGCGAAATGAGAAGGAGGCAGCGGGTTCATCTGTTGGTGGACGAAGCAGGACTCCATCCAAGATGAGGCTCACTACTTGGTACGTGAAGCCAATGCTGATCCATAAAGCCACTC [illegible]
4_c26     ACGTGGGCAGTACTCGTACGCAGCTGCGCCAAGCACTCCGCGAAATGAGAAGGAGGCAGCGGGTTCATCTGTTGGTGGACGAAGCAGGACTCCATCCAAGATGAGGCTCACTACTTGGTACGTGAAGCCAATGCTGATCCATAAAGCCACTC [illegible]
4_c25     CGTTGGGCAGTACTCGTCAGCGGCTGCTTCAGGCACCCCGCGAAATGAGAAGGAGGCAGCGGGTTCATCTGTTGGTGGACGAAGCAGGACTCCATCCAAGATGAGGCTCACTACTTGGTACGTGAAGCCGATCATGACCCATAAA---AGAC [illegible]
5_c14     ACGTGGGCAGTACTCGTACGCAGCTGCGCCAAGCACTCCGCGAAATGAGAAGGAGGCAGCGGGTTCATCTGTTGGTGGACGAAGCAGGACTCCATCCAAGATGAGGCTCACTACTTGGTACGTGAAGCCAATGCTGATCCATAAAGCCACTC [illegible]
5_c0      ACGTGGGCAGTACTCGTTAGCGGCTGCTTCAGGCACCCCGCGAAATGAGAAGGAGGCAGCGGGTTCATCTGTTGGTGGACGAAGCAGGACTCCATCCAAGATGAGGCTCACTACTTGGTACGTGAAGCCGATCATGACCTATGAA---AGAC [illegible]
6_c0      ACGTGGGCAGTACTCGTACGCAGCTGCGCCAAGCACTCCGCGAAATGAGAAGGAGGCAGCGGGTTCATCTGTTGGTGGACGAAGCAGGACTCCATCCAAGATGAGGCTCACTACTTGGTACGTGAAGCCAATGCTGATCCATAAAGCCACTC [illegible]
6_c1      ACGTGGGCAGTACTCGTTAGCGGCTGCTTCAGGCACCCCGCGAAATGAGAAGGAGGCAGCGGGTTCATCTGTTGGTGGACGAAGCAGGACTCCATCCAAGATGAGGCTCACTACTTGGTACGTGAAGCCGATCATGACCTATGAA---AGAC [illegible]
7_c1      ACGTGGGCAGTACTCGTACGCAGCTGCGCCAAGCACTCCGCGAAATGAGAAGGAGGCAGCGGGTTCATCTGTTGGTGGACGAAGCAGGACTCCATCCAAGATGAGGCTCACTACTTGGTACGTGAAGCCAATGCTGATCCATAAAGCCACTC [illegible]
7_c0      ACGTGGGCAGTACTCGTTAGCGGCTGCTTCAGGCACCCCGCGAAATGAGAAGGAGGCAGCGGGTTCATCTGTTGGTGGACGAAGCAGGACTCCATCCAAGATGAGGCTCACTACTTGGTACGTGAAGCCGATCATGACCTATGAA---AGAC [illegible]
8_c145    ------------------------------------------------------------ [illegible]
8_c478    --------AGTACTTGTCTGCGGCTGCTTCAGGCACCCCGCGAAATGAGAAGGAAGCAGCGGTTTCATCTGTTGGTGGACGAAGCAGGACTCCATCGAAGATGAGGCTCACTACTTGGTATGTGAAGCAGATCATAGTCCATGAA---AGCC [illegible]
9_c69     ACGTGGACAGCACTCGTCAGCGGCTGCTTTAGGCGTCCCGCGAAATGAGAAGGAGGCAGCGGCTTCATCTGCTGGTGGACGAAGCAGGACTCCATCCAAGATGAGGCTCACTACTTGGTACGTGAAGCCGATCATGACCCATAAA---AGAC [illegible]
10_c2514  ------------------------------------------------------------ [illegible]
10_c2762  ------------------------------------------------------------ [illegible]
10_c17    ACGTGGGCAGTACTCGTTAGCGGCTGCTTCAGGCACCCCGCGAAATGAGAAGGAGGCAGCGGGTTCATCTGTTGGTGGACGAAGCAGGACTCCATCCAAGATGAGGCTCACTACTTGGTACGTGAAGCCGATCATGACCTATGAA---AGAC [illegible]
10_c1502  ACGTGGGCAGTACTCGTACGCAGCTGCGCCAAGCACTCCGCGAAATGAGAAGGAGGCAGCGGGTTCATCTGTTGGTGGACGAAGCAGGACTCCATCCAAGATGAGGCTCACTACTTGGTACGTGAAGCCAATGCTGATCCATAAAGCCACTC [illegible]
10_c2103  ------------------------------------------------------------ [illegible]
10_c3521  ------------------------------------------------------------ [illegible]
10_c6715  ------------------------------------------------------------ [illegible]
10_c3485  ------------------------------------------------------------ [illegible]
10_c4919  ------------------------------------------------------------ [illegible]
11_c443   ACGTGGGCAGTACTCGTACGCAGCTGCGCCAAGCACTCCGCGAAATGAGAAGGAGGCAGCGGGTTCATCTGTTGGTGGACGAAGCAGGACTCCATCCAAGATGAGGCTCACTACTTGGTACGTGAAGCCAATGCTGATCCATAAAGCCACTC [illegible]
11_c378   ACGTGAGCAGTACTTGTCTGCGGCTGCTTCAGGCACCCCGCGAAATGAGAAGGAAGCAGCGGTTTCATCTGTTGGTGGACGAAGCAGGACTCCATCGAAGATGAGGCTCACTACTTGGTATGTGAAGCAGATCATAGTCCATGAA---AGCC [illegible]
12_c25    ACGTGAGCAGTACTTGACTGCAGCTGCTTCAGGCACCCCGCGAAATGAGAAGGAGGCAGCGGGTTCATCTGTTGGTGGACGAAACAGGACTCCATCCAAGATGAGGCTCACTACTTGGTATGTGAAGCAGATCATAGTCC-TCAA---AGCC [illegible]
13_c555   ------------------------------------------------------------ [illegible]
13_c189   ACGTGGGCAGTACTCGTACGCAGCTGCGCCAAGCACTCCGCGAAATGAGAAGGAGGCAGCGGGTTCATCTGTTGGTGGACGAAGCAGGACTCCATCCAAGATGAGGCTCACTACTTGGTACGTGAAGCCAATGCTGATCCATAAAGCCACTC [illegible]
13_c74    ACGTGAGCAGTACTTGTCTGCGGCTGCTTCAGGCACCCCGCGAAATGAGAAGGAAGCAGCGGTTTCATCTGTTGGTGGACGAAGCAGGACTCCATCCAAGATGAGGCTGACTACTTGGTATGTGAAGCAGATCATAGTCCATGAA---AGCC [illegible]
14_c324   ACGTGGGCAGTTCACGCA-GCGGCTGCTTCGGGTGCACCACGAAATGAGCAGGAGGCAGCGGGTTCATCTGTTGGTGGACGAAGCAGGACTCCATCCAAGATGAGGCTCACTACTTGGTACGTGAAGCCGATGCTGATCCGGAAAGCCACTC [illegible]
14_c329   ACGTGAGCAGTACTTGTCTGCGGCTGCTTCAGGCACCCCGCGAAATGAGAAGGAAGCAGCGGTTTCATCTGTTGGTGGACGAAGCAGGACTCCATCGAAGATGAGGCTCACTACTTGGTATGTGAAGCAGATCATAGTCCATGAA---AGCC [illegible]
15_c188   ACGTGGGCAGTACTCGTACGCAGCTGCGCCAAGCACTCCGCGAAATGAGAAGGAGGCAGCGGGTTCATCTGTTGGTGGACGAAGCAGGACTCCATCCAAGATGAGGCTCACTACTTGGTACGTGAAGCCAATGCTGATCCATAAAGCCACTC [illegible]
15_c161   ACGTGGGCAGTACTCGTTAGCGGCTGCTTCAGGCACCCCGCGAAATGAGAAGGAGGCAGCGGGTTCATCTGTTGGTGGACGAAGCAGGACTCCATCCAAGATGAGGCTCACTACTTGGTACGTGAAGCCGATCATGACCTATGAA---AGAC [illegible]
16_c36    GCGTGAGCAATACTCGTCAGTGGTTGCTTCAGGCACCCCACGAA [illegible]
17_c39    ACGTAGGCAATACTTGTCTGCGGCTGCTTCAGGCACCCCGCGAA [illegible]
18_c0     ACGTGAGCAGTACTTGACTGCAGCTGCTTCAGGCACCCCGCGAA [illegible]
19_c229   ACGTGGGCAGTTCACGCA-GCGGCTGCTTCGGGTGCACCACGAA [illegible]
19_c2     ACGTGAGCAGTACTTGTCTGCGGCTGCTTCAGGCACCCCGCGAA [illegible]
20_c93    ACGAGGGCAGTTCACGCA-GCGGCTACTTCAGGCGCCCCACGAA [illegible]
21_c78    ACGAGGGCAGTTCACGCA-GCGGCTACTTCAGGCGCCCCACGAA [illegible]
22_c436   GCGTGGCCAATACTTGTCTGCGGCTGCTTCAGGCACCCCGCGAA [illegible]
22_c3703  ------------------------------------------------------------ [illegible]
22_c3909  ------------------------------------------------------------ [illegible]
22_c3246  ------------------------------------------------------------ [illegible]
22_c3239  ------------------------------------------------------------ [illegible]
22_c9147  ------------------------------------------------------------ [illegible]
22_c9143  ------------------------------------------------------------ [illegible]
22_c3243  ACGTGAGCAGTACTTGACTGCGGCTGCTTCAGGCACCCCGCGAA [illegible]
22_c7804  ------------------------------------------------------------ [illegible]
22_c8090  ------------------------------------------------------------ [illegible]
23_c97    GCGTGGACAATACTTGTCTGCGGCTGCTTCAGGCACCCCGCGAA [illegible]
24_c78    ACGAGGGCAGTTCACGCA-GCGGCTACTTCAGGCGCCCCACGAA [illegible]
25_c2     ACGTGGGCAGTACTCGTACGCAGCTGCGCCAAGCACTCCGCGAA [illegible]
25_c4     ACGTGGGCAGTACTCGTTAGCGGCTGCTTCAGGCACCCCGCGAA [illegible]
26_c640   ACGTGAGCAGTACTTGACTGCGGCTGCTTCAGGCACCCCGCGAA [illegible]
26_c3091  ------------------------------------------------------------ [illegible]
26_c3507  ------------------------------------------------------------ [illegible]
26_c8864  ------------------------------------------------------------ [illegible]
26_c9308  ------------------------------------------------------------ [illegible]
26_c5920  ------------------------------------------------------------ [illegible]
26_c5576  ------------------------------------------------------------ [illegible]
26_c8886  ------------------------------------------------------------ [illegible]
26_c2334  ------------------------------------------------------------ [illegible]
26_c8891  ------------------------------------------------------------ [illegible]
27_c2847  ------------------------------------------------------------ [illegible]
27_c2264  ------------------------------------------------------------ [illegible]
27_c1238  ------------------------------------------------------------ [illegible]
27_c8054  ------------------------------------------------------------ [illegible]
27_c8053  ------------------------------------------------------------ [illegible]
27_c2660  ACGTGGGCAATACTTGTCTGCGGCTGCTTCGGGCACTCCGCGAA [illegible]
27_c2066  ------------------------------------------------------------ [illegible]
27_c2887  ACGTGAGCAGTAATTGTCTGCGGCTGCTTCAGGCACCCCGCGAA [illegible]
27_c9392  ------------------------------------------------------------ [illegible]
27_c9396  ------------------------------------------------------------ [illegible]
27_c7764  ------------------------------------------------------------ [illegible]
28_c53    ACGGGGGCAATTCACGCA-GCGGCTACTTCAGGCGCCCCACGAA [illegible]
29_c114   ACGTGGGCAGTTCACGCA-GCGGCTGCTTCGGGTGCACCACGAA [illegible]
29_c19    ACGTGAGCAGTACTTGTCTGCGGCTGCTTCAGGCACCCCGCGAA [illegible]
30_c1     ACGTGGGCAGTACTCGTACGCAGCTGCGCCAAGCACTCCGCGAA [illegible]
30_c16    ACGTGGGCAGTACTCGTTAGCGGCTGCTTCAGGCACCCCGCGAA [illegible]
31_c6068  ------------------------------------------------------------ [illegible]
31_c1849  ------------------------------------------------------------ [illegible]
31_c2683  ------------------------------------------------------------ [illegible]
31_c7078  ------------------------------------------------------------ [illegible]
31_c3712  ------------------------------------------------------------ [illegible]
31_c8783  ------------------------------------------------------------ [illegible]
31_c4585  ------------------------------------------------------------ [illegible]
31_c11607 ------------------------------------------------------------ [illegible]
31_c11132 ------------------------------------------------------------ [illegible]
31_c11215 ------------------------------------------------------------ [illegible]
32_c1495  ------------------------------------------------------------ [illegible]
32_c2827  ------------------------------------------------------------ [illegible]
32_c1902  ------------------------------------------------------------ [illegible]
32_c6288  ------------------------------------------------------------ [illegible]
32_c4518  ------------------------------------------------------------ [illegible]
32_c10289 ------------------------------------------------------------ [illegible]
32_c10286 ------------------------------------------------------------ [illegible]
32_c12407 ------------------------------------------------------------ [illegible]
33_c3225  ------------------------------------------------------------ [illegible]
33_c1289  ------------------------------------------------------------ [illegible]
33_c3588  ------------------------------------------------------------ [illegible]
33_c3633  ------------------------------------------------------------ [illegible]
33_c3542  ------------------------------------------------------------ [illegible]
33_c8070  ACGTGGACAGCACTCGTCAGCGGCTGCTTTAGGCGTCCCGCGAA [illegible]
34_c2333  ------------------------------------------------------------ [illegible]
34_c2748  ------------------------------------------------------------ [illegible]
34_c2702  ------------------------------------------------------------ [illegible]
34_c2106  ------------------------------------------------------------ [illegible]
34_c3824  ------------------------------------------------------------ [illegible]
34_c12455 ------------------------------------------------------------ [illegible]
35_c3229  ------------------------------------------------------------ [illegible]
35_c3040  ------------------------------------------------------------ [illegible]
35_c2986  ------------------------------------------------------------ [illegible]
35_c3517  ------------------------------------------------------------ [illegible]
35_c7295  ------------------------------------------------------------ [illegible]
35_c3583  ------------------------------------------------------------ [illegible]
35_c11501 ------------------------------------------------------------ [illegible]
35_c11581 ------------------------------------------------------------ [illegible]
36_c87    ACGTGGGCAGTACTCGTACGCAGCTGCGCCAAGCACTCCGCGAA [illegible]
36_c2900  ------------------------------------------------------------ [illegible]
36_c10327 ------------------------------------------------------------ [illegible]
36_c5231  ------------------------------------------------------------ [illegible]
36_c3613  ------------------------------------------------------------ [illegible]
36_c6503  ------------------------------------------------------------ [illegible]
36_c5437  ------------------------------------------------------------ [illegible]
36_c17033 ------------------------------------------------------------ [illegible]
36_c7222  ------------------------------------------------------------ [illegible]
36_c25053 ------------------------------------------------------------ [illegible]
36_c9137  ------------------------------------------------------------ [illegible]
36_c25163 ------------------------------------------------------------ [illegible]
36_c27098 ------------------------------------------------------------ [illegible]
36_c10144 ------------------------------------------------------------ [illegible]
36_c29808 ------------------------------------------------------------ [illegible]
36_c29806 ------------------------------------------------------------ [illegible]
36_c16773 ACGTGGGCAGTACTCGTTAGCGGCTGCTTCAGGCACCCCGCGAA [illegible]
37_c0     ACGTGGGCAGTACTCGTACGCAGCTGCGCCAAGCACTCCGCGAA [illegible]
37_c122   ACGTGGGCAGTACTCGTTAGCGGCTGCTTCAGGCACCCCGCGAA [illegible]
38_c2     ACGTGGGCAGTACTCGTACGCAGCTGCGCCAAGCACTCCGCGAA [illegible]
38_c21    ACGTGGGCAGTACTCGTTAGCGGCTGCTTCAGGCACCCCGCGAA [illegible]
39_c79    ACGAGGGCAGTTCACGCA-GCGGCTACTTCAGGCGCCCCACGAA [illegible]
40_c22    ACGTGGGCAGTACTCGTACGCAGCTGCGCCAAGCACTCCGCGAA [illegible]
40_c16    ACGTGGGCAGTACTCGTTAGCGGCTGCTTCAGGCACCCCGCGAA [illegible]
41_c16    ACGTGGGCAGTACTCGTACGCAGCTGCGCCAAGCACTCCGCGAA [illegible]
41_c18    ACGTGGGCAGTACTCGTTAGCGGCTGCTTCAGGCACCCCGCGAA [illegible]
42_c3     ACGTGGGCAGTACTCGTACGCAGCTGCGCCAAGCACTCCGCGAA [illegible]
42_c17    ACGTGGGCAGTACTCGTTAGCGGCTGCTTCAGGCACCCCGCGAA [illegible]
43_c11    ACGTGGGCAGTACTCGTACGCAGCTGCGCCAAGCACTCCGCGAA [illegible]
43_c0     ACGTGGGCAGTACTCGTTAGCGGCTGCTTCAGGCACCCCGCGAA [illegible]
44_c76    ACGAGGGCAGTTCACGCA-GCGGCTACTTCAGGCGCCCCACGAA [illegible]
45_c2879  ------------------------------------------------------------ [illegible]
45_c2088  ------------------------------------------------------------ [illegible]
45_c2272  ACGTGGGCAATACTTGTCTGCGGCTGCTTCGGGCACTCCGCGAA [illegible]
45_c1584  ------------------------------------------------------------ [illegible]
45_c8010  ------------------------------------------------------------ [illegible]
45_c8487  ------------------------------------------------------------ [illegible]
45_c2110  ------------------------------------------------------------ [illegible]
45_c7764  ------------------------------------------------------------ [illegible]
46_c568   ACGTGAGCAGTACTTGACTGCGGCTGCTTCAGGCACCCCGCGAA [illegible]
46_c2890  ------------------------------------------------------------ [illegible]
46_c3787  ------------------------------------------------------------ [illegible]
46_c8833  ------------------------------------------------------------ [illegible]
46_c9063  ----------------------------------------------A [illegible]
46_c5867  ------------------------------------------------------------ [illegible]
46_c5875  ------------------------------------------------------------ [illegible]
46_c4243  ------------------------------------------------------------ [illegible]
46_c5253  ------------------------------------------------------------ [illegible]
46_c4182  ------------------------------------------------------------ [illegible]
47_c654   ACGTGAGCAGTACTTGACTGCGGCTGCTTCAGGCACCCCGCGAA [illegible]
47_c866   ACGTGAGCAGTAATTGTCTGCGGCTGCTTCAGGCACCCCGCGAA [illegible]
47_c1813  ------------------------------------------------------------ [illegible]
47_c2201  ------------------------------------------------------------ [illegible]
47_c8816  ------------------------------------------------------------ [illegible]
47_c4862  ------------------------------------------------------------ [illegible]
47_c8829  ------------------------------------------------------------ [illegible]
47_c2374  ------------------------------------------------------------ [illegible]
48_c59    ACGTGGACAGCACTCGTCAGCGGCTGCTTTAGGCGTCCCGCGAA [illegible]
49_c38    ACGTGGACAGCACTCGTCAGCGGCTGCTTTAGGCGTCCCGCGAA [illegible]
50_c1244  ACGTGAGCAGTACTTGACTGCGGCTGCTTCAGGCACCCCGCGAA [illegible]
50_c3508  ------------------------------------------------------------ [illegible]
50_c3451  ------------------------------------------------------------ [illegible]
50_c3379  ------------------------------------------------------------ [illegible]
50_c3455  ------------------------------------------------------------ [illegible]
50_c4315  ------------------------------------------------------------ [illegible]
50_c3055  GCGTGGCCAATACTTGTCTGCGGCTGCTTCAGGCACCCCGCGAA [illegible]
50_c9016  ------------------------------------------------------------ [illegible]
50_c10606 ------------------------------------------------------------ [illegible]
50_c7788  ------------------------------------------------------------ [illegible]
50_c7790  ------------------------------------------------------------ [illegible]
51_c344   ACGTGGGCAGTTCACGCA-GCGGCTGCTTCGGGTGCACCACGAA [illegible]
51_c19    ACGTGAGCAGTACTTGTCTGCGGCTGCTTCAGGCACCCCGCGAA [illegible]
52_c81    ACGAGGGCAGTTCACGCA-GCGGCTACTTCAGGCGCCCCACGAA [illegible]
53_c1     ACGTGGGCAGTACTCGTACGCAGCTGCGCCAAGCACTCCGCGAA [illegible]
53_c16    ACGTGGGCAGTACTCGTTAGCGGCTGCTTCAGGCACCCCGCGAA [illegible]
54_c2     ACGTGGGCAGTACTCGTACGCAGCTGCGCCAAGCACTCCGCGAA [illegible]
54_c23    ACGTGGGCAGTACTCGTTAGCGGCTGCTTCAGGCACCCCGCGAA [illegible]
A_c86     ACGTGGGCAGTACTCGTACGCAGCTGCGCCAAGCACTCCGCGAA [illegible]
A_c234    ------------------------------------------------------------ [illegible]
A_c179    ACGTGGGCAGTACTCGTTAGCGGCTGCTTCAGGCACCCCGCGAA [illegible]
B_c29     ------------------------------------------------------------ [illegible]
B_c167    ACGTGGGCAGTACTCGTTAGCGGCTGCTTCAGGCACCCCGCGAA [illegible]
B_c233    ACGTGGGCAGTACTCGTACGCAGCTGCGCCAAGCACTCCGCGAA [illegible]
C_c82     ACGTGGGCAGTACTCGTCAGCGGCTGCTTCAGGCACCCCGCGAA [illegible]
D_c114    ------------------------------------------------------------ [illegible]
D_c220    ACGAGGGCAGTTCACGCA-GCGGCTACTTCAGGCGCCCCACGAA [illegible]
E_c281    ACGTGGACAGCACTCGTCAGCGGCTGCTTTAGGCGTCCCGCGAA [illegible]
F_c333    ACGTGGACAGTACTCGTCAGCGGCTGCTTCAGGCGTCCCGCGAA [illegible]
F_c277    ACGTGAGCAGTAATTGTCTGCGGCTGCTTCAGGCACCCCGCGAA [illegible]
G_c217    ACGTGGACAGCACTCGTCAGCGGCTGCTTTAGGCGTCCCGCGAA [illegible]
G_c346    ACGTGAGCAATACTCGTCAGCGGCTGCTTCAGGCACCCCGCGAG [illegible]
G_c3223   ------------------------------------------------------------ [illegible]
G_c2752   ------------------------------------------------------------ [illegible]
H_c257    GCGTGGCCAATACTTGTCTGCGGCTGCTTCAGGCACCCCGCGAA [illegible]
H_c316    ACGTGAGCAGTACTTGACTGCGGCTGCTTCAGGCACCCCGCGAA [illegible]
H_c803    ------------------------------------------------------------ [illegible]
H_c809    ------------------------------------------------------------ [illegible]
I_c2398   ------------------------------------------------------------ [illegible]
I_c6      ACGTGAGCAGTACTTGACTGCGGCTGCTTCAGGCACCCCGCGAA [illegible]
I_c211    ACGTGAGCAGTAATTGTCTGCGGCTGCTTCAGGCACCCCGCGAA [illegible]
I_c2862   ------------------------------------------------------------ [illegible]
I_c2904   ------------------------------------------------------------ [illegible]
I_c4553   ------------------------------------------------------------ [illegible]
I_c3789   ------------------------------------------------------------ [illegible]
J_c192    ACGTGAGCAGTACTTGACTGCGGCTGCTTCAGGCACCCCGCGAA [illegible]
J_c435    ACGTGAGCAGTAATTGTCTGCGGCTGCTTCAGGCACCCCGCGAA [illegible]
J_c3365   ------------------------------------------------------------ [illegible]
J_c3372   ------------------------------------------------------------ [illegible]
J_c3012   ------------------------------------------------------------ [illegible]
J_c531    ------------------------------------------------------------ [illegible]
J_c4022   ------------------------------------------------------------ [illegible]
K_c2135   ------------------------------------------------------------ [illegible]
K_c1206   ------------------------------------------------------------ [illegible]
K_c93     ACGTGGGCAATACTTGTCTGCGGCTGCTTCGGGCACTCCGCGAA [illegible]
K_c1438   ------------------------------------------------------------ [illegible]
K_c3555   ------------------------------------------------------------ [illegible]
K_c461    ACGTGAGCAGTAATTGTCTGCGGCTGCTTCAGGCACCCCGCGAA [illegible]
L_c1200   ------------------------------------------------------------ [illegible]
L_c1178   ------------------------------------------------------------ [illegible]
L_c383    ACGTGAGCAGTAATTGTCTGCGGCTGCTTCAGGCACCCCGCGAA [illegible]
L_c3401   ------------------------------------------------------------ [illegible]
L_c444    ACGTGGG
```

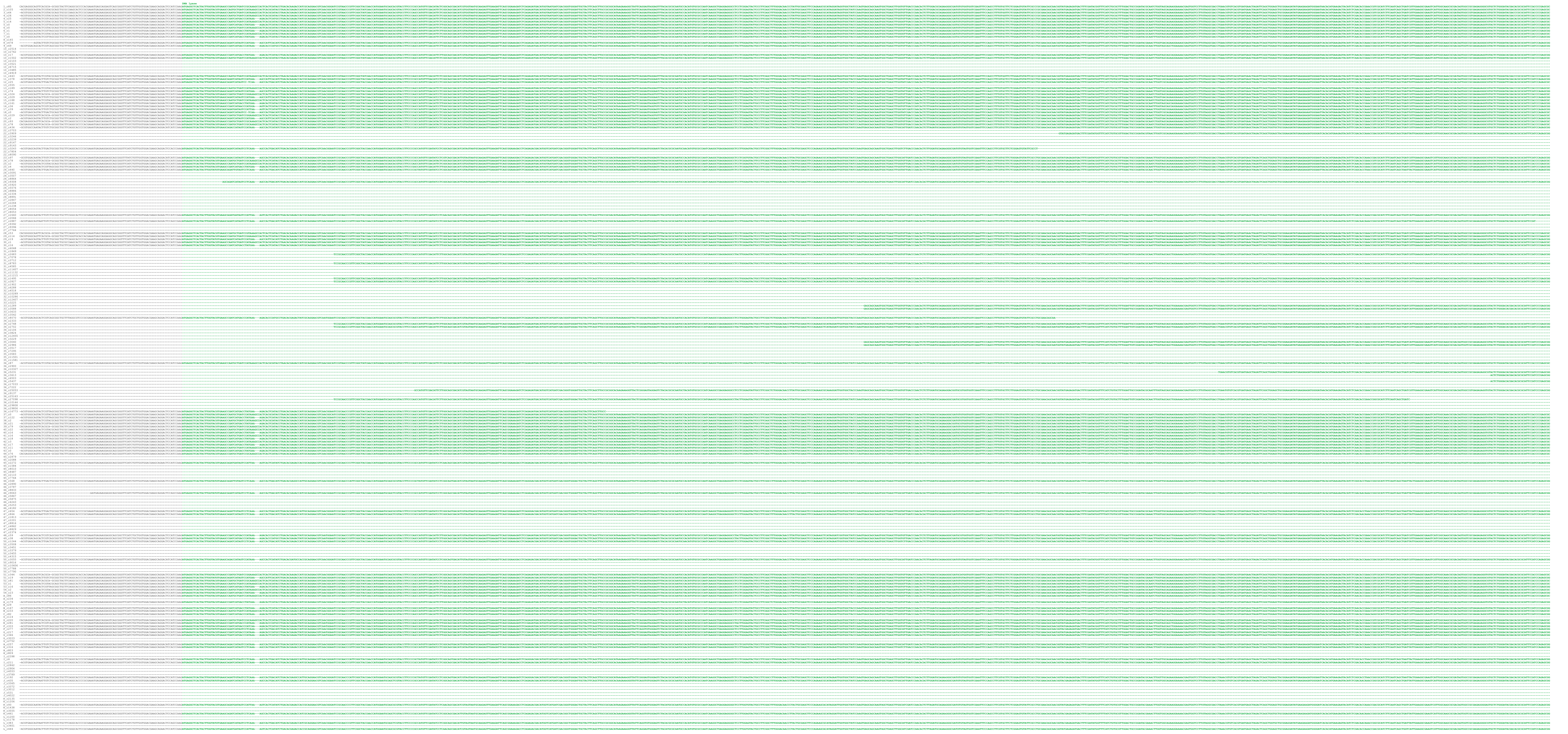
DNA lyase

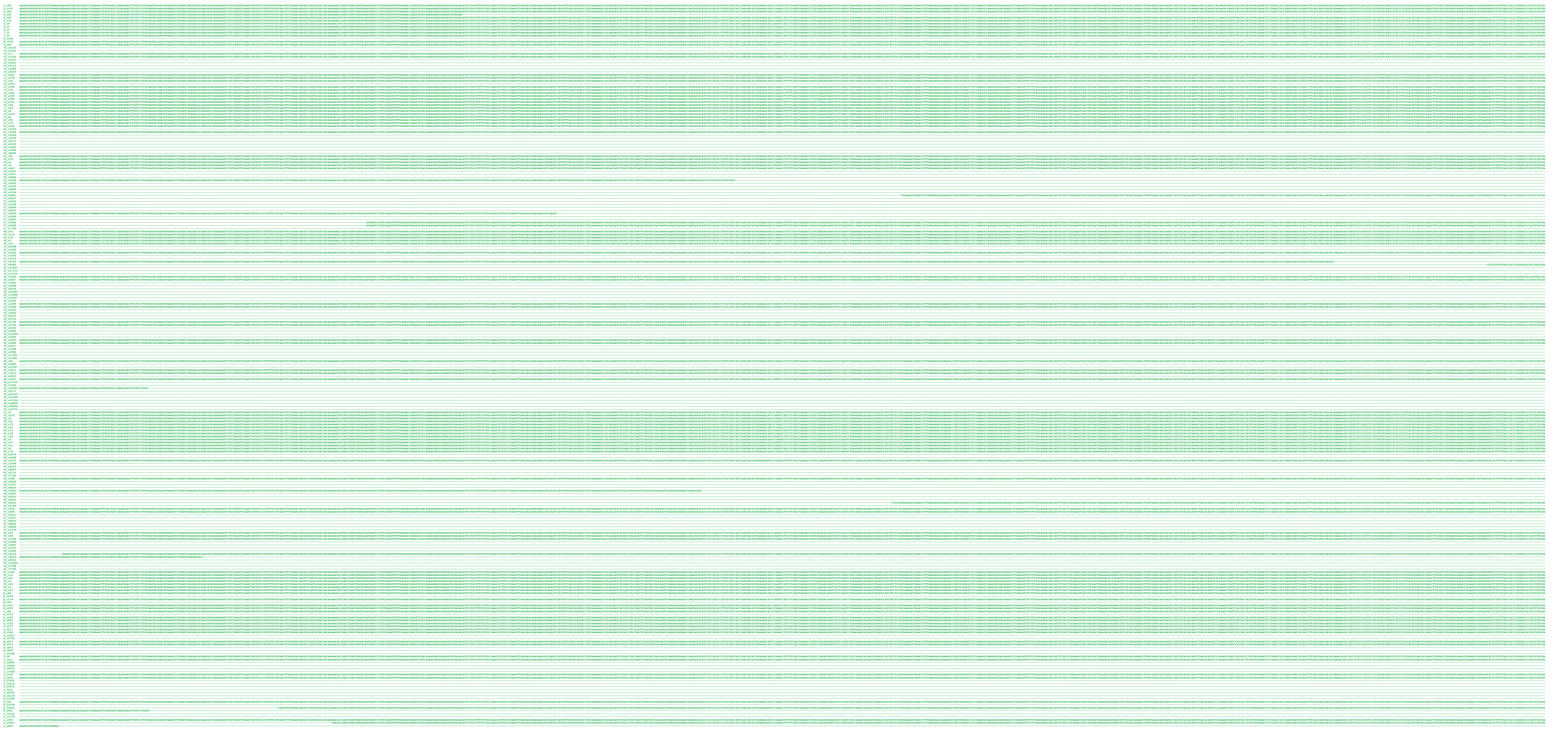

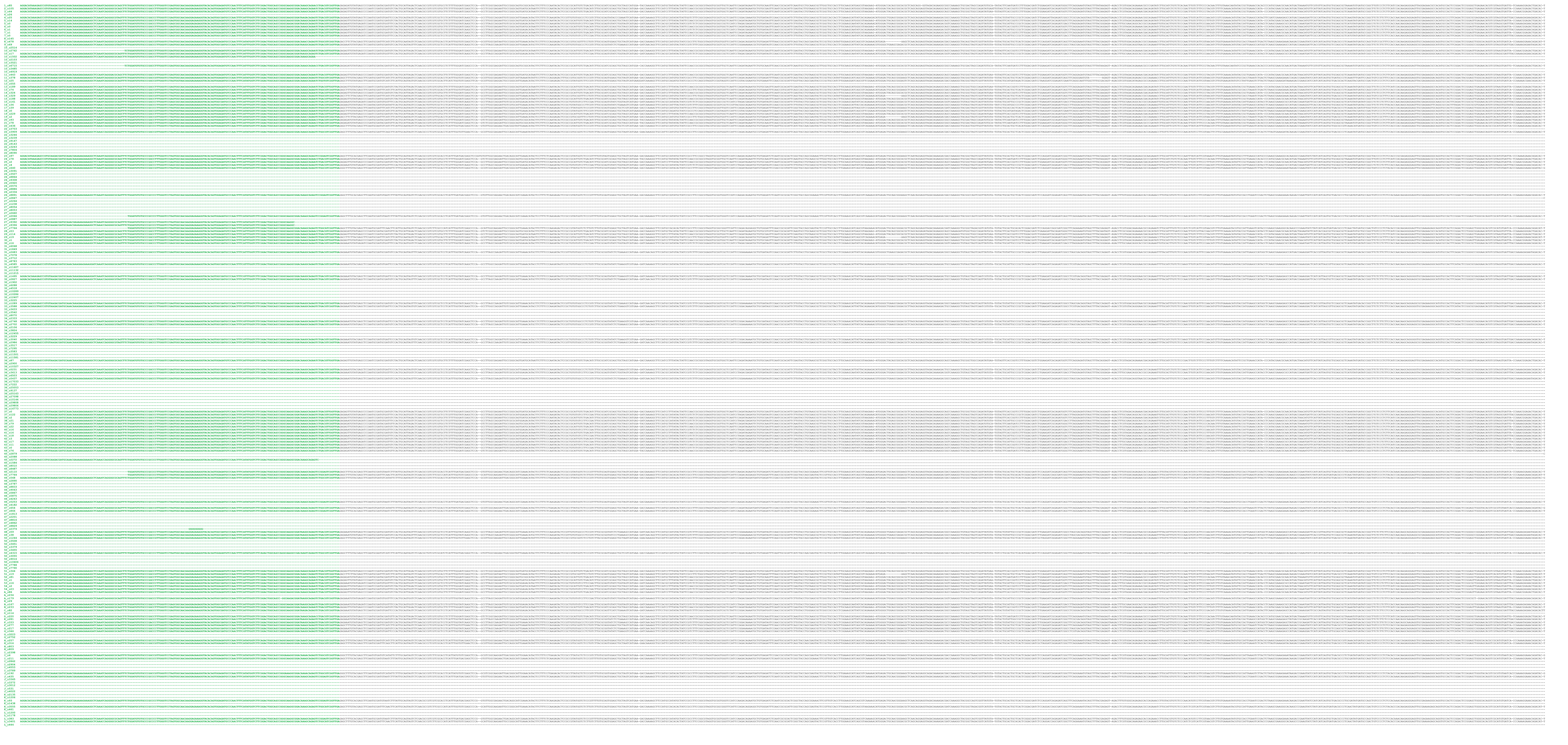

```
1_c85     TT-GGCACAGCGGAAGATGG---------------------------------------------------TTTTGCAAATA-TGCTTGTGTACAGGGGA-----------------CTAACTACTTGCTA-TGTTTCCGCCAGA [illegible]
2_c105    TT-GGCACAGTGGAAGATGG---------------------------------------------------TTTTGCAATCA-TGCTTGTGTACAGGGGA-----------------CTAACTACTTGCTA-TGTTTCCGCCAGA [illegible]
3_c64     TT-GGCACAGTGGAAGATGG---------------------------------------------------TTTTGCAATCA-TGCTTGTGTACAGGGGA-----------------CTAACTACTTGCTA-TGTTTCCGCCAGA [illegible]
4_c26     -------------AAGATGG---------------------------------------------------TTTTGCAATCA-TGCTTGTGTACAGGGGA-----------------CTAACTACTTGCTA-TGTTTCCGCCAGA [illegible]
4_c25     TT-GACAGAGCGGAAGATAGT-------------------------------------------------TTTTTTGCAATCA-TGCTTGTGTACAGAGTA-----------------CTAACTACTTGCTA-TGTTTCCGCCAGA [illegible]
5_c14     TT-GGCACAGTGGAAGATGG---------------------------------------------------TTTTGCAATCA-TGCTTGTGTACAGGGGA-----------------CTAACTACTTGCTA-TGTTTCCGCCAGA [illegible]
5_c0      TT-GACAGAGCGGAAGATAGTT------------------------------------------------TTTTTTGCAATCA-TGCTTGTGTACAGAGTA-----------------CTTACTACTTGCTA-TGTTTCCGCCAGA [illegible]
6_c0      TT-GGCACAGTGGAAGATGG---------------------------------------------------TTTTGCAATCA-TGCTTGTGTACAGGGGA-----------------CTAACTACTTGCTA-TGTTTCCGCCAGA [illegible]
6_c1      TT-GACAGAGCGGAAGATAGTT------------------------------------------------TTTTTTGCAATCA-TGCTTGTGTACAGAGTA-----------------CTAACTACTTGCTA-TGTTTCCGCCAGA [illegible]
7_c1      TT-GGCACAGTGGAAGATGG---------------------------------------------------TTTTGCAATCA-TGCTTGTGTACAGGGGA-----------------CTAACTACTTGCTA-TGTTTCCGCCAGA [illegible]
7_c0      TT-GACAGAGCGGAAGATAGTT------------------------------------------------TTTTTTGCAATCA-TGCTTGTGTACAGAGTA-----------------CTTACTACTTGCTA-TGTTTCCGCCAGA [illegible]
8_c145    --------------------------------------------------------------------------------------------------------------------------------------------- [illegible]
8_c478    TTAGGCCGAGAGGGTGATGG---------------------------------------------------TTATGTCATCATAGCTTGAATACA-GGGGGT----------TTTG--CTAACTACATGCTGCTGCTTCTGCCAGG [illegible]
9_c69     TT-GACAGAGTGCGAGATAG--------------------------------------------------TTTTTGCAATCA-TGCTTGTGTACAGGGTA-----------------CTAACTACTTGCAG-TGTTTCCGCCAGA [illegible]
10_c2514  --------------------------------------------------------------------------------------------------------------------------------------------- [illegible]
10_c2762  TT-GGCACAGTGGAAGATGG---------------------------------------------------TTTTGCAATCA-TGCTTGTGTACAGGGGA-----------------CTAACTACTTGCTA-TGTTTCCGCCAGA [illegible]
10_c17    TT-GACAGAGCGGAAGATAGTT------------------------------------------------TTTTTTGCAATCA-TGCTTGTGTACAGAGTA-----------------CTTACTACTTGCTA-TGTTTCCGCCAGA [illegible]
10_c1502  --------------------------------------------------------------------------------------------------------------------------------------------- [illegible]
10_c2103  --------------------------------------------------------------------------------------------------------------------------------------------- [illegible]
10_c3521  --------------------------------------------------------------------------------------------------------------------------------------------- [illegible]
10_c6715  TT-GGCACAGCGGAAGATGG---------------------------------------------------TTTTGCAACTA-TGCTTATGTACAGGGGA-----------------CTAACTACTTGCTA-TGTTTCCGCCAGA [illegible]
10_c3485  --------------------------------------------------------------------------------------------------------------------------------------------- [illegible]
10_c4919  --------------------------------------------------------------------------------------------------------------------------------------------- [illegible]
11_c443   TT-GGCACAGTGGAAGATGG---------------------------------------------------TTTTGCAATCA-TGCTTGTGTACAGGGGA-----------------CTAACTACTTGCTA-TGTTTCCGCCAGA [illegible]
11_c378   TTAGGCCGAGAGGGTGATGGTTATGTCATCATAGCTTGAATACAGGGGGTTTTCTTTTAGGCCGAGAGGGTGATGGTTATGTCATCATAGCTTGAATACA-GGGGGT----------TTTG--CTAACTACATGCTGTTGCTTCTGCCAGA [illegible]
12_c25    TT-GGCAAAGCAGAAGATGG---------------------------------------------------TTTTGCAATCATTGCTTGAATACA-GGGGGT----------TTTG--CTAACTACATGCTGTTGCTTCTGCCAGA [illegible]
13_c555   --------------------------------------------------------------------------------------------------------------------------------------------- [illegible]
13_c189   TT-GGCACAGTGGAAGATGG---------------------------------------------------TTTTGCAATCA-TGCTTGTGTACAGGGGA-----------------CTAACTACTTGCTA-TGTTTCCGCCAGA [illegible]
13_c74    TGAAACCGAGAAGGAGATAG---------------------------------------------------TTTTGCAATCATTGCTTGAATACAGGGGGGG----------TTTG--CTAACTACATGCTGTTGCTTCTGCCAGA [illegible]
14_c324   TT-GGCACAGTGGAAGATGG---------------------------------------------------TTTTGCAATCA-TGCTTGTGTACAGGGGA-----------------CTAACTACTTGCTA-TGTTTCCGCCAGA [illegible]
14_c329   TTAGGCCGAGAGGGTGATGG---------------------------------------------------TTATGTCATCATAGCTTGAATACA-GGGGGT----------TTTG--CTAACTACATGCTGCTGCTTCTGCCAGG [illegible]
15_c188   TT-GGCACAGTGGAAGATGG---------------------------------------------------TTTTGCAATCA-TGCTTGTGTACAGGGGA-----------------CTAACTACTTGCTA-TGTTTCCGCCAGA [illegible]
15_c161   TT-GACAGAGCGGAAGATAGTT------------------------------------------------TTTTTTGCAATCA-TGCTTGTGTACAGAGTA-----------------CTTACTACTTGCTA-TGTTTCCGCCAGA [illegible]
16_c36    TT-GACACGGCGGAAGATAG--------------------------------------------------TTTTTCGCAATCA-TGCTTGTGTACAGGGGGTG---------------CTAACTACTTGCTA-CGTTTCCGCCAGA [illegible]
17_c39    TT-GGCAGAGCAGAAGATAG--------------------------------------------------TTTTGCAATCATTGCTTGAGTACAGGGGGG----------TTTG--CTAACTACATGCTGTTGCTTCTGCCAGA [illegible]
18_c0     TT-GGCAAAGCAGAAGATGG---------------------------------------------------TTTTGCAATCATTGCTTGAATACA-GGGGGT----------TTTG--CTAACTACATGCTGTTGCTTCTGCCAGA [illegible]
19_c229   TT-GGCACAGTGGAAGATGG---------------------------------------------------TTTTGCAATCA-TGCTTGTGTACAGGGGA-----------------CTAACTACTTGCTA-TGTTTCCGCCAGA [illegible]
19_c2     TTAGGCCGAGAGGGTGATGG---------------------------------------------------TTATGTCATCATAGCTTGAATACA-GGGGGT----------TTTG--CTAACTACATGCTGCTGCTTCTGCCAGG [illegible]
20_c93    TT-GGCACAGCGGAAGATGG---------------------------------------------------TTTTGCAACTA-TGCTTGTGTACAGGGGA-----------------CTAACTACTTGCTA-TGTTTCCGCCAGA [illegible]
21_c78    TT-GGCACAGCGGAAGATGG---------------------------------------------------TTTTGCAACTA-TGCTTGTGTACAGGGGA-----------------CTAACTACTTGCTA-TGTTTCCGCCAGA [illegible]
22_c436   TT-GGCAGAGCGGAAGATAG--------------------------------------------------TTTTGCAATCATTGCTTGAATACAGGGGGGT----------TTTG--CTAACTACATGCTGTTGCTTCTGCCAGA [illegible]
22_c3703  --------------------------------------------------------------------------------------------------------------------------------------------- [illegible]
22_c3909  TGAAACCGAGAAGGAGATAG---------------------------------------------------TTTTGCAATCATTGCTTGAATACAGGGGGGG----------TTTG--CTAACTACATGCTGTTGCTTCTGCCAGA [illegible]
22_c3246  --------------------------------------------------------------------------------------------------------------------------------------------- [illegible]
22_c3239  --------------------------------------------------------------------------------------------------------------------------------------------- [illegible]
22_c9147  --------------------------------------------------------------------------------------------------------------------------------------------- [illegible]
22_c9143  --------------------------------------------------------------------------------------------------------------------------------------------- [illegible]
22_c3243  --------------------------------------------------------------------------------------------------------------------------------------------- [illegible]
22_c7804  --------------------------------------------------------------------------------------------------------------------------------------------- [illegible]
22_c8090  --------------------------------------------------------------------------------------------------------------------------------------------- [illegible]
23_c97    TT-GGCAGAGCGGAAGATAG--------------------------------------------------TTTTGCAATCATTGCTTGAATACAGGGGGGT----------TTTG--CTAACTACATGCTGTTGCTTCTGCCAGA [illegible]
24_c78    TT-GGCACAGCGGAAGATGG---------------------------------------------------TTTTGCAACTA-TGCTTGTGTACAGGGGA-----------------CTAACTACTTGCTA-TGTTTCCGCCAGA [illegible]
25_c2     TT-GGCACAGTGGAAGATGG---------------------------------------------------TTTTGCAATCA-TGCTTGTGTACAGGGGA-----------------CTAACTACTTGCTA-TGTTTCCGCCAGA [illegible]
```

[illegible]

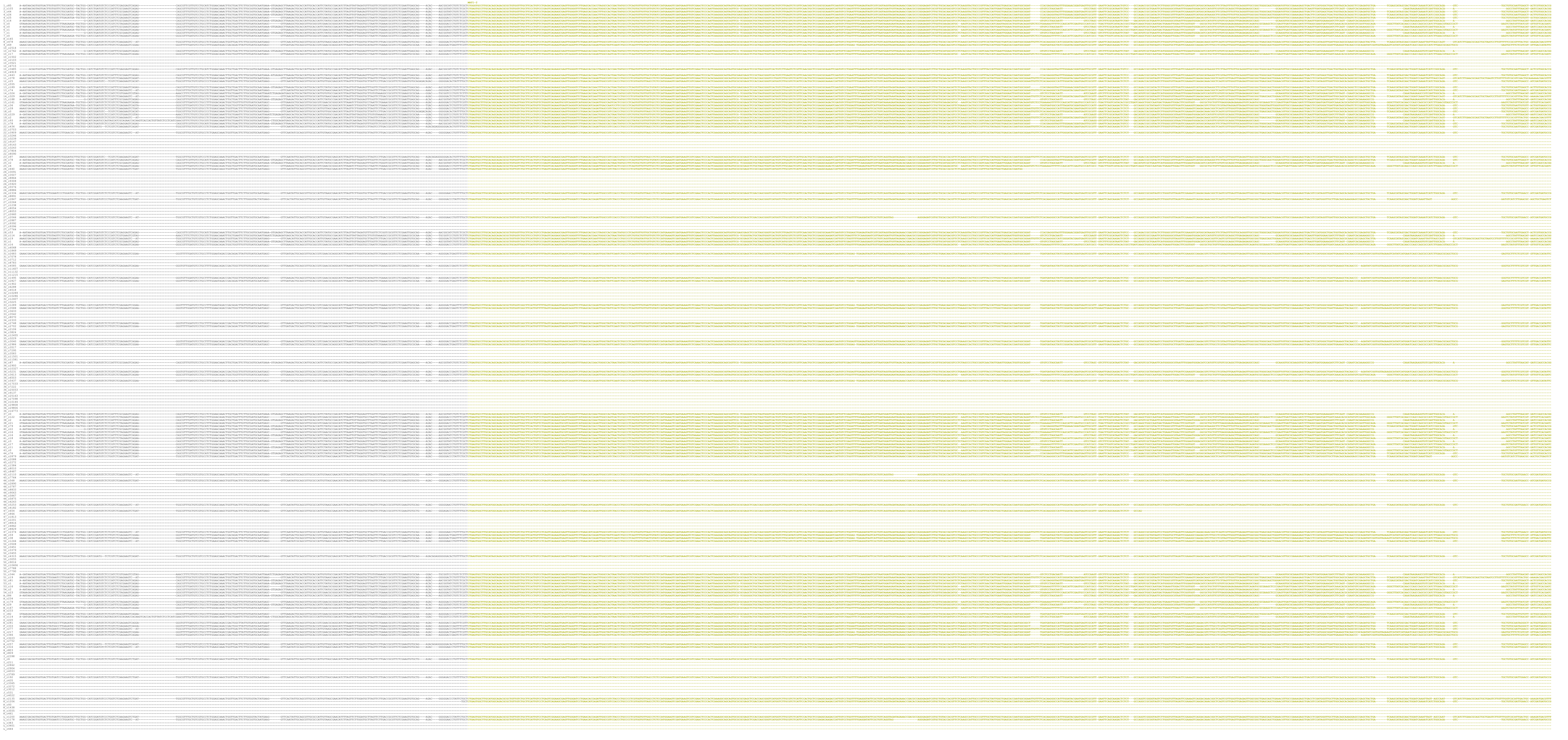
MAT1-2

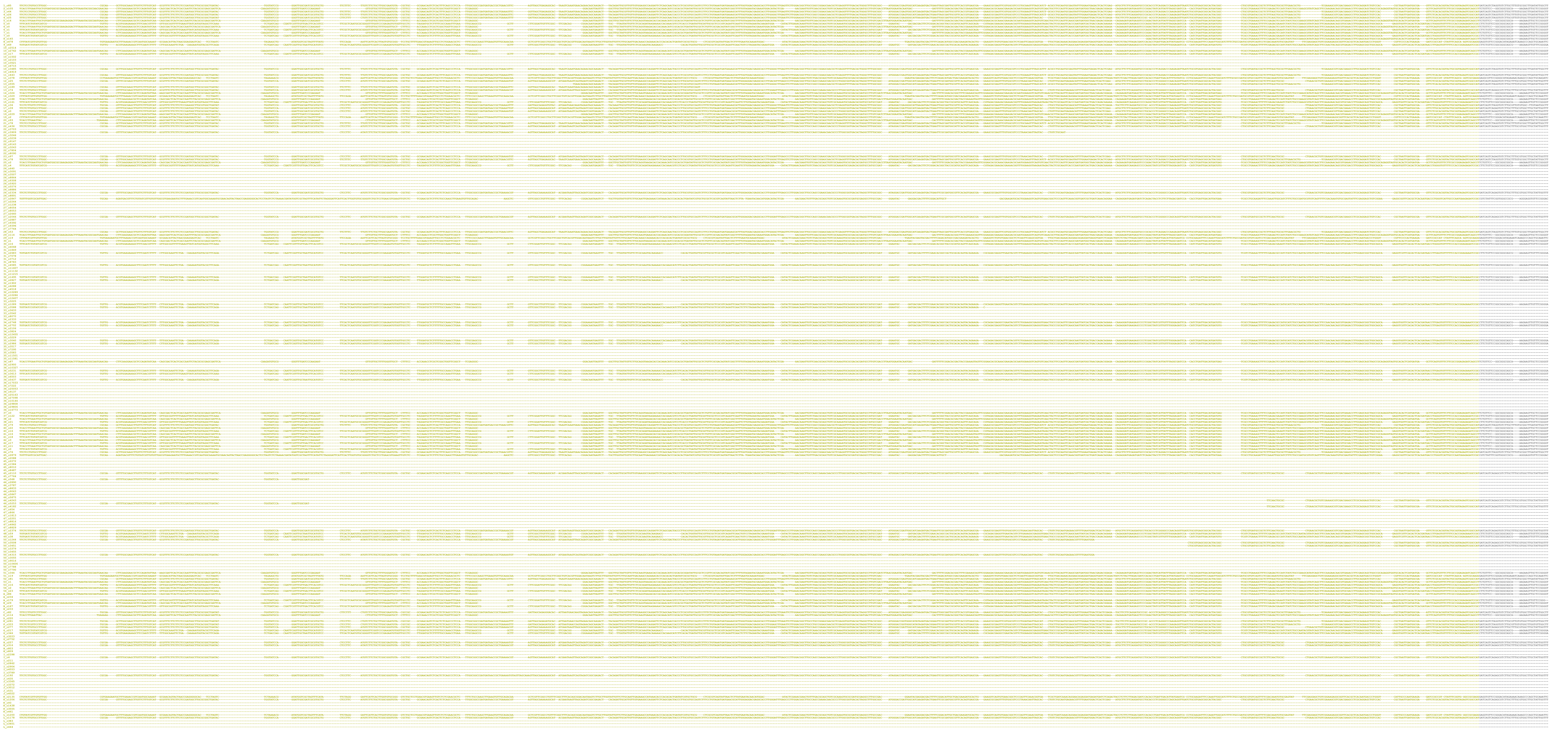

```
1_c85      GAAAAATACAGTTCGAAAGTATAGGTGTGAAATGTGACGGTC---------------------GTACGGAATGAAAGGGCTCTCGCCGTCAACTTGAAAGTGCACC-AAAA [illegible]
2_c105     ATTGAAAACAGAGCCGAGCTCCAAGTTCAGAGGTTCCTGATCTTGAAAAATTTTGAGCAGGCAGAGGAGGCAATGAGATATCCCTCCCAATGTTCCGGGTCACTCAAT--TAT [illegible]
3_c64      G-AAAATACAGTTCGAAAGTATAGGTGTGAAATGTGACAGTC---------------------GTACGGAATGAAAGGGCTCTCGCCGTCAACTTGAAAGTGCACC--AAA [illegible]
4_c26      G-AAAATACAGTTCGAAAGTATAGGTGTGAAATGTGACAGTC---------------------GTACGGAATGAAAGGGCTCTCGCCGTCAACTTGAAAGTGCACC--AAA [illegible]
4_c25      GGAAAATACAATTCGAAAGTACAGGTGCGAAATGTGACGGTC-----------------GTACGGAGTGAAAGGACTTTCGCCGTTCAACTGTATAATGCGCCAAAA----- [illegible]
5_c14      ATTGAAAACAGAGCCGAGCTCCAAGTTCAGAGGTTCCTGATCTTGAAAAATTTTGAGCAGGCAGAGGAGGCAATGAGATATCCCTCCCAATGTTCCGGGTCACTCAAT--TAT [illegible]
5_c0       ATTGAAAACAGGGCCAAGCTCCAAGTCCAGAGATTGCTTATCTTGAAGCATTTTGAGCAGGTAGAAGAGGCGTCGAGATATCGCTTCCAATGCTCCGGAGCACTCAAT--TAT [illegible]
6_c0       ATTGAAAACAGAGCCGAGCTCCAAGTTCAGAGGTTCCTGATCTTGAAAAATTTTGAGCAGGCAGAGGAGGCAATGAGATATCCCTCCCAATGTTCCGGGTCACTCAAT--TAT [illegible]
6_c1       ATTGAAAACAGGGCCAAGCTCCAAGTCCAGAGATTGCTTATCTTGAAGCATTTTGAGCAGGTAGAAGAGGCGTCGAGATATCGCTTCCAATGCTCCGGAGCACTCAAT--TAT [illegible]
7_c1       ATTGAAAACAGAGCCGAGCTCCAAGTTCAGAGGTTCCTGATCTTGAAAAATTTTGAGCAGGCAGAGGAGGCAATGAGATATCCCTCCCAATGTTCCGGGTCACTCAAT--TAT [illegible]
7_c0       ATTGAAAACAGGGCCAAGCTCCAAGTCCAGAGATTGCTTATCTTGAAGCATTTTGAGCAGGTAGAAGAGGCGTCGAGATATCGCTTCCAATGCTCCGGAGCACTCAAT--TAT [illegible]
8_c145     ATTGAAAACAGAGCCGAGCTCCAAGTTCAGAGGTTCCTGATCTTGAAAAATTTTGAGCAGGCAGAGGAGGCTATGAGATATCCCTCCCAATGTTCCGGGTCACTCAAT--TAT [illegible]
8_c478     AGAG-----ATTTGACAGCTTGAAGCGTTTTGAGCAGGGAGA---------------------GGAGGCAATGAGATATTCCTCGCAATGCCACGGAGCACTTGAT--TAC [illegible]
9_c69      AGAGAGAACAGGGCTAAGCTCCAAGTCCAGAGATTCCTCATCTTGAAGCATTTTGAGCAAGTAGAGGAGGCAATGAGATATCGCATCCAATGTTCCGGGGCACTCAGT--TAT [illegible]
10_c2514   ------------------------------------------------------------------------------------------------------------ [illegible]
10_c2762   ATTGAAAACAGAGCCGAGCTCCAAGTTCAGAGGTTCCTGATCTTGAAAAATTTTGAGCAGGCAGAGGAGGCAATGAGATATCCCTCCCAATGTTCCGGGTCACTCAAT--TAT [illegible]
10_c17     ATTGAAAACAGGGCCAAGCTCCAAGTCCAGAGATTGCTTATCTTGAAGCATTTTGAGCAGGTAGAAGAGGCGTCGAGATATCGCTTCCAATGCTCCGGAGCACTCAAT--TAT [illegible]
10_c1502   ------------------------------------------------------------------------------------------------------------ [illegible]
10_c2103   ------------------------------------------------------------------------------------------------------------ [illegible]
10_c3521   ------------------------------------------------------------------------------------------------------------ [illegible]
10_c6715   ------------------------------------------------------------------------------------------------------------ [illegible]
10_c3485   GAAAAATACAGTTCGAAAGTATAGGTGTGAAATGTGACGGTC---------------------GTACGGAATGAAAGGGCTCTCGCCGTCAACTTGAAAGTGCACC-AAAA [illegible]
10_c4919   ------------------------------------ [illegible]
11_c443    G-AAAATACAGTTCGAAAGTATAGGTGTGAAATGTG [illegible]
11_c378    AGAG-----ATTTGACATGTTTAAGCATTTTGAGCA [illegible]
12_c25     GAAAGATACGGTTCGAAAGTATAGGTGTGAAATGTG [illegible]
13_c555    G-AAAATACAGTTCGAAAGTATAGGTGTGAAATGTG [illegible]
13_c189    ------------------------------------ [illegible]
13_c74     GAAAGAAACGATTGAAAAGTATAGGTGTGAAATTTG [illegible]
14_c324    ATTGAAAACAGAGCCGAGCTCCAAGTTCAGAGGTTC [illegible]
14_c329    AGAG-----ATTTGACAGCTTGAAGCGTTTTGAGCA [illegible]
15_c188    ATTGAAAACAGAGCCGAGCTCCAAGTTCAGAGGTTC [illegible]
15_c161    ATTGAAAACAGGGCCAAGCTCCAAGTCCAGAGATTG [illegible]
16_c36     GAAAAATACAATTCGAAAGTATAGGTGTGAAATGTG [illegible]
17_c39     GAAAGATACGATTCGAAAGTATAGGTGTGAGAAGTG [illegible]
18_c0      GAAAGATACGGTTCGAAAGTATAGGTGTGAAATGTG [illegible]
19_c229    ATTGAAAACAGAGCCGAGCTCCAAGTTCAGAGGTTC [illegible]
19_c2      AGAG-----ATTTGACAGCTTGAAGCGTTTTGAGCA [illegible]
20_c93     ATTGAAGACAGAGCCGAGCTCCAAGTTCAGAGGTTC [illegible]
21_c78     GAAAAATACAGTTCGAAAGTATAGGTGTGAAATGTG [illegible]
22_c436    GAAAGAAACGATTGAAAAGTATAGGTGTGAAATGTG [illegible]
22_c3703   ------------------------------------ [illegible]
22_c3909   ------------------------------------ [illegible]
22_c3246   ------------------------------------ [illegible]
22_c3239   ------------------------------------ [illegible]
22_c9147   ------------------------------------ [illegible]
22_c9143   ------------------------------------ [illegible]
22_c3243   ------------------------------------ [illegible]
22_c7804   ------------------------------------ [illegible]
22_c8090   ------------------------------------ [illegible]
23_c97     GAAAGATACGGTTCGAAAGTATAGGTGTGAAATGTG [illegible]
24_c78     GAAAAATACAGTTCGAAAGTATAGGTGTGAAATGTG [illegible]
25_c2      ATTGAAAACAGAGCCGAGCTCCAAGTTCAGAGGTTC [illegible]
25_c4      ATTGAAAACAGGGCCAAGCTCCAAGTCCAGAGATTG [illegible]
26_c640    ------------------------------------ [illegible]
26_c3091   ------------------------------------ [illegible]
26_c3507   ------------------------------------ [illegible]
26_c8864   ------------------------------------ [illegible]
26_c9308   ------------------------------------ [illegible]
26_c5920   ------------------------------------ [illegible]
26_c5576   ------------------------------------ [illegible]
26_c8886   ------------------------------------ [illegible]
26_c2334   GAAAGATACGATTCGAAAGTATAGGTGTGAAATTTG [illegible]
26_c8891   ------------------------------------ [illegible]
27_c2847   ATAGAAAACAAAGCCCAGCTGCAAATTCAGAGATTC [illegible]
27_c2264   ------------------------------------ [illegible]
27_c1238   ------------------------------------ [illegible]
27_c8054   ------------------------------------ [illegible]
27_c8053   ------------------------------------ [illegible]
27_c2660   ------------------------------------ [illegible]
27_c2066   GAAAGAAACGATTGAAAAGTATAGGTGTGAAATGTG [illegible]
27_c2887   ------------------------------------ [illegible]
27_c9392   ------------------------------------ [illegible]
27_c9396   ------------------------------------ [illegible]
27_c7764   ------------------------------------ [illegible]
28_c53     GAAAAATACAGTTCGAAAGTATAGGTGTGAAATGTG [illegible]
29_c114    ATTGAAAACAGAGCCGAGCTCCAAGTTCAGAGGTTC [illegible]
29_c19     AGAG-----ATTTGACAGCTTGAAGCGTTTTGAGCA [illegible]
30_c1      ATTGAAAACAGAGCCGAGCTCCAAGTTCAGAGGTTC [illegible]
30_c16     ATTGAAAACAGGGCCAAGCTCCAAGTCCAGAGATTG [illegible]
31_c6068   ------------------------------------ [illegible]
31_c1849   ------------------------------------ [illegible]
31_c2683   ATAGAGAACAGGGCTAAGCTCCAAGTCCAGAGATTC [illegible]
31_c7078   ------------------------------------ [illegible]
31_c3712   ------------------------------------ [illegible]
31_c8783   ------------------------------------ [illegible]
31_c4585   ATAGAAAACAGAGCTAAGCTCCAAGTCCAGAGATTC [illegible]
31_c11607  ------------------------------------ [illegible]
31_c11132  ------------------------------------ [illegible]
31_c11215  ------------------------------------ [illegible]
32_c1495   ATAGAAAACAGAGCTAAGCTCCAAGTCCAGAGATTC [illegible]
32_c2827   ATAGAGAACAGGGCT--------------------- [illegible]
32_c1902   ------------------------------------ [illegible]
32_c6288   ------------------------------------ [illegible]
32_c4518   ------------------------------------ [illegible]
32_c10289  ------------------------------------ [illegible]
32_c10286  ------------------------------------ [illegible]
32_c12407  ------------------------------------ [illegible]
33_c3225   ------------------------------------ [illegible]
33_c1289   ATAGAAAACAGAGCTAAGCTCCAAGTCCAGAGATTC [illegible]
33_c3588   ------------------------------------ [illegible]
33_c3633   ------------------------------------ [illegible]
33_c3542   ------------------------------------ [illegible]
33_c8070   ------------------------------------ [illegible]
34_c2333   ------------------------------------ [illegible]
34_c2748   ATAGAAAACAGAGCTAAGCTCCAAGTCCAGAGATTC [illegible]
34_c2702   ATAGAGAACAGGGCTAAGCTCCAAGTCCAGAGATTC [illegible]
34_c2106   ------------------------------------ [illegible]
34_c3824   ------------------------------------ [illegible]
34_c12455  ------------------------------------ [illegible]
35_c3229   ------------------------------------ [illegible]
35_c3040   ATAGAAAACAGAGCTAAGCTCCAAGTCCAGAGATTC [illegible]
35_c2986   ATAGAGAACAGGGCTAAGCTCCAAGTCCAGAGATTC [illegible]
35_c3517   ------------------------------------ [illegible]
35_c7295   ------------------------------------ [illegible]
35_c3583   ------------------------------------ [illegible]
35_c11501  ------------------------------------ [illegible]
35_c11581  ------------------------------------ [illegible]
36_c87     ATTGAAAACAGAGCCGAGCTCCAAGTTCAGAGGTTC [illegible]
36_c2900   ------------------------------------ [illegible]
36_c10327  ------------------------------------ [illegible]
36_c5231   ATAGAAAACAGAGCTAAGCTCCAAGTCCAGAGATTC [illegible]
36_c3613   ATTGAAAACAGGGCCAAGCTCCAAGTCCAGAGATTG [illegible]
36_c6503   ------------------------------------ [illegible]
36_c5437   ATAGAGAACAGGGCTAAGCTCCAAGTCCAGAGATTC [illegible]
36_c17033  ------------------------------------ [illegible]
36_c7222   ------------------------------------ [illegible]
36_c25053  ------------------------------------ [illegible]
36_c9137   ------------------------------------ [illegible]
36_c25163  ------------------------------------ [illegible]
36_c27098  ------------------------------------ [illegible]
36_c10144  ------------------------------------ [illegible]
36_c29808  ------------------------------------ [illegible]
36_c29806  ------------------------------------ [illegible]
36_c16773  ------------------------------------ [illegible]
37_c0      ATTGAAAACAGAGCCGAGCTCCAAGTTCAGAGGTTC [illegible]
37_c122    ATTGAAAACAGGGCCAAGCTCCAAGTCCAGAGATTG [illegible]
38_c2      ATTGAAAACAGAGCCGAGCTCCAAGTTCAGAGGTTC [illegible]
38_c21     ATTGAAAACAGGGCCAAGCTCCAAGTCCAGAGATTG [illegible]
39_c79     GAAAAATACAGTTCGAAAGTATAGGTGTGAAATGTG [illegible]
40_c22     ATTGAAAACAGAGCCGAGCTCCAAGTTCAGAGGTTC [illegible]
40_c16     ATTGAAAACAGGGCCAAGCTCCAAGTCCAGAGATTG [illegible]
41_c16     ATTGAAAACAGAGCCGAGCTCCAAGTTCAGAGGTTC [illegible]
41_c18     ATTGAAAACAGGGCCAAGCTCCAAGTCCAGAGATTG [illegible]
42_c3      ATTGAAAACAGAGCCGAGCTCCAAGTTCAGAGGTTC [illegible]
42_c17     ATTGAAAACAGGGCCAAGCTCCAAGTCCAGAGATTG [illegible]
43_c11     ATTGAAAACAGAGCCGAGCTCCAAGTTCAGAGGTTC [illegible]
43_c0      ATTGAAAACAGGGCCAAGCTCCAAGTCCAGAGATTG [illegible]
44_c76     GAAAAATACAGTTCGAAAGTATAGGTGTGAAATGTG [illegible]
45_c2879   ATAGAAAACAAAGCCCAGCTGCAAATTCAGAGATTC [illegible]
45_c2088   ------------------------------------ [illegible]
45_c2272   ------------------------------------ [illegible]
45_c1584   ------------------------------------ [illegible]
45_c8010   ------------------------------------ [illegible]
45_c8487   ------------------------------------ [illegible]
45_c2110   GAAAGAAACGATTGAAAAGTATAGGTGTGAAATATG [illegible]
45_c7764   ------------------------------------ [illegible]
46_c568    ------------------------------------ [illegible]
46_c2890   ------------------------------------ [illegible]
46_c3787   ------------------------------------ [illegible]
46_c8833   ------------------------------------ [illegible]
46_c9063   ------------------------------------ [illegible]
46_c5867   ------------------------------------ [illegible]
46_c5875   ------------------------------------ [illegible]
46_c4243   GAAAGAAACGATTGAAAAGTATAGGTGTGAAATGTG [illegible]
46_c5253   ------------------------------------ [illegible]
46_c4182   GAAAGATACGATTCGAAAGTATAGGTGTGAAATTTG [illegible]
47_c654    ------------------------------------ [illegible]
47_c866    ------------------------------------ [illegible]
47_c1813   ------------------------------------ [illegible]
47_c2201   ------------------------------------ [illegible]
47_c8816   ------------------------------------ [illegible]
47_c4862   ------------------------------------ [illegible]
47_c8829   ------------------------------------ [illegible]
47_c2374   GAAAGATACGATTCGAAAGTATAGGTGTGAAATTTG [illegible]
48_c59     ATAGAGAACAGGGCTAAGCTCCAAGTCCAGAGATTC [illegible]
49_c38     ATAGAGAACAGGGCTAAGCTCCAAGTCCAGAGATTC [illegible]
50_c1244   ------------------------------------ [illegible]
50_c3508   GAAAGAAACGATTGAAAAGTATAGGTGTGAAATGTG [illegible]
50_c3451   GAAAGATACGGTTCGAAAGTATAGGTGTGAAATGTG [illegible]
50_c3379   ------------------------------------ [illegible]
50_c3455   ------------------------------------ [illegible]
50_c4315   ------------------------------------ [illegible]
50_c3055   ------------------------------------ [illegible]
50_c9016   ------------------------------------ [illegible]
50_c10606  ------------------------------------ [illegible]
50_c7788   ------------------------------------ [illegible]
50_c7790   ------------------------------------ [illegible]
51_c344    ATTGAAAACAGAGCCGAGCTCCAAGTTCAGAGGTTC [illegible]
51_c19     AGAG-----ATTTGACAGCTTGAAGCGTTTTGAGCA [illegible]
52_c81     GAAAAATACAGTTCGAAAGTATAGGTGTGAAATGTG [illegible]
53_c1      ATTGAAAACAGAGCCGAGCTCCAAGTTCAGAGGTTC [illegible]
53_c16     ATTGAAAACAGGGCCAAGCTCCAAGTCCAGAGATTG [illegible]
54_c2      ATTGAAAACAGAGCCGAGCTCCAAGTTCAGAGGTTC [illegible]
54_c23     ATTGAAAACAGGGCCAAGCTCCAAGTCCAGAGATTG [illegible]
A_c86      ATTGAAAACAGAGCCGAGCTCCAAGTTCAGAGGTTC [illegible]
A_c234     ------------------------------------ [illegible]
A_c179     ------------------------------------ [illegible]
B_c29      ATTGAAAACAGAGCCGAGCTCCAAGTTCAGAGGTTC [illegible]
B_c167     ATTGAAAACAGGGCCAAGCTCCAAGTCCAGAGATTG [illegible]
B_c233     ------------------------------------ [illegible]
C_c82      GGAAAATACAATTCGAAAGTACAGGTGCGAAATGTG [illegible]
D_c114     ATTGAAGACAGAGCCGAGCTCCAAGTTCAGAGGTTC [illegible]
D_c220     ------------------------------------ [illegible]
E_c281     GAAAAATACAATTCGAAAGTATAGGTGTGAAATGTG [illegible]
F_c333     GAAAAATACAATTCGAAAGTATAGGTGTGAAATGTG [illegible]
F_c277     GAAAGAAACGATTGAAAAGTATAGGTGTGAAATGTG [illegible]
G_c217     ATAGAGAACAGGGCTAAGCTCCAAGTCCAGAGATTC [illegible]
G_c346     ATAGAAAACAGAGCTAAGCTCCAAGTCCAGAGATTC [illegible]
G_c3223    ------------------------------------ [illegible]
G_c2752    ------------------------------------ [illegible]
H_c257     GAAAGATACGGTTCGAAAGTATAGGTGTGAAATGTG [illegible]
H_c316     GAAAGAAACGATTGAAAAGTATAGGTGTGAAATGTG [illegible]
H_c803     ------------------------------------ [illegible]
H_c809     ------------------------------------ [illegible]
I_c2398    ------------------------------------ [illegible]
I_c6       GAAAGAAACGATTGAAAAGTATAGGTGTGAAATGTG [illegible]
I_c211     ------------------------------------ [illegible]
I_c2862    ------------------------------------ [illegible]
I_c2904    ------------------------------------ [illegible]
I_c4553    ------------------------------------ [illegible]
I_c3789    ------------------------------------ [illegible]
J_c192     GAAAGAAACGATTGAAAAGTATAGGTGTGAAATGTG [illegible]
J_c435     ------------------------------------ [illegible]
J_c3365    ------------------------------------ [illegible]
J_c3372    ------------------------------------ [illegible]
J_c3012    ------------------------------------ [illegible]
J_c531     ------------------------------------ [illegible]
J_c4022    ------------------------------------ [illegible]
K_c2135    AGAG-----ATTCGACTTTTTGAAGGATTTTGAGCA [illegible]
K_c1206    GAAAGAAACGATTGAAAAGTATAGGTGTGAAATGTG [illegible]
K_c93      ------------------------------------ [illegible]
K_c1438    ------------------------------------ [illegible]
K_c3555    ------------------------------------ [illegible]
K_c461     ------------------------------------ [illegible]
L_c1200    AGAG-----ATTCGACTTTTTGAAGGATTTTGAGCA [illegible]
L_c1178    GAAAGAAACGATTGAAAAGTATAGGTGTGAAATATG [illegible]
L_c383     ------------------------------------ [illegible]
L_c3401    ------------------------------------ [illegible]
L_c444     ------------------------------------ [illegible]
```

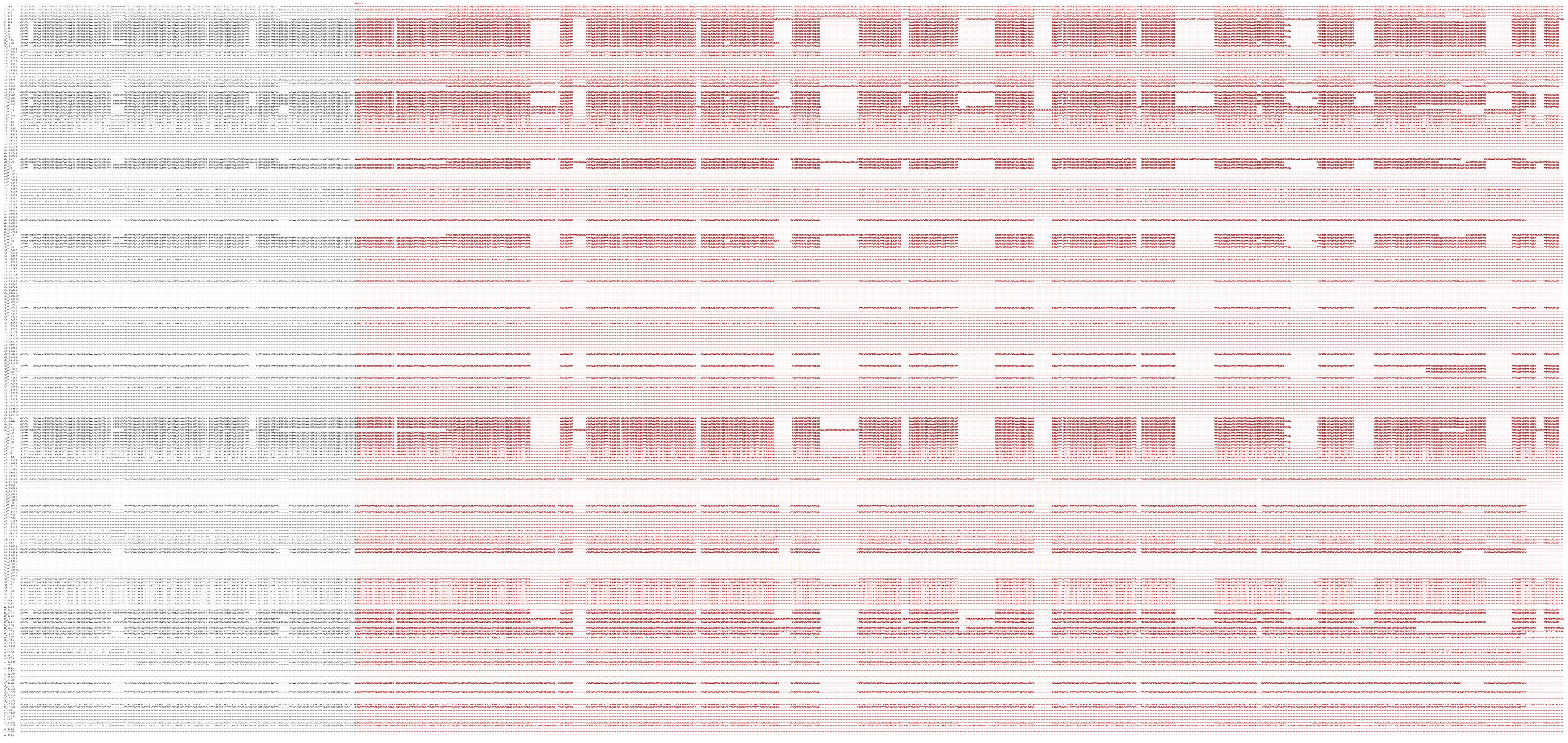
MAT1-1

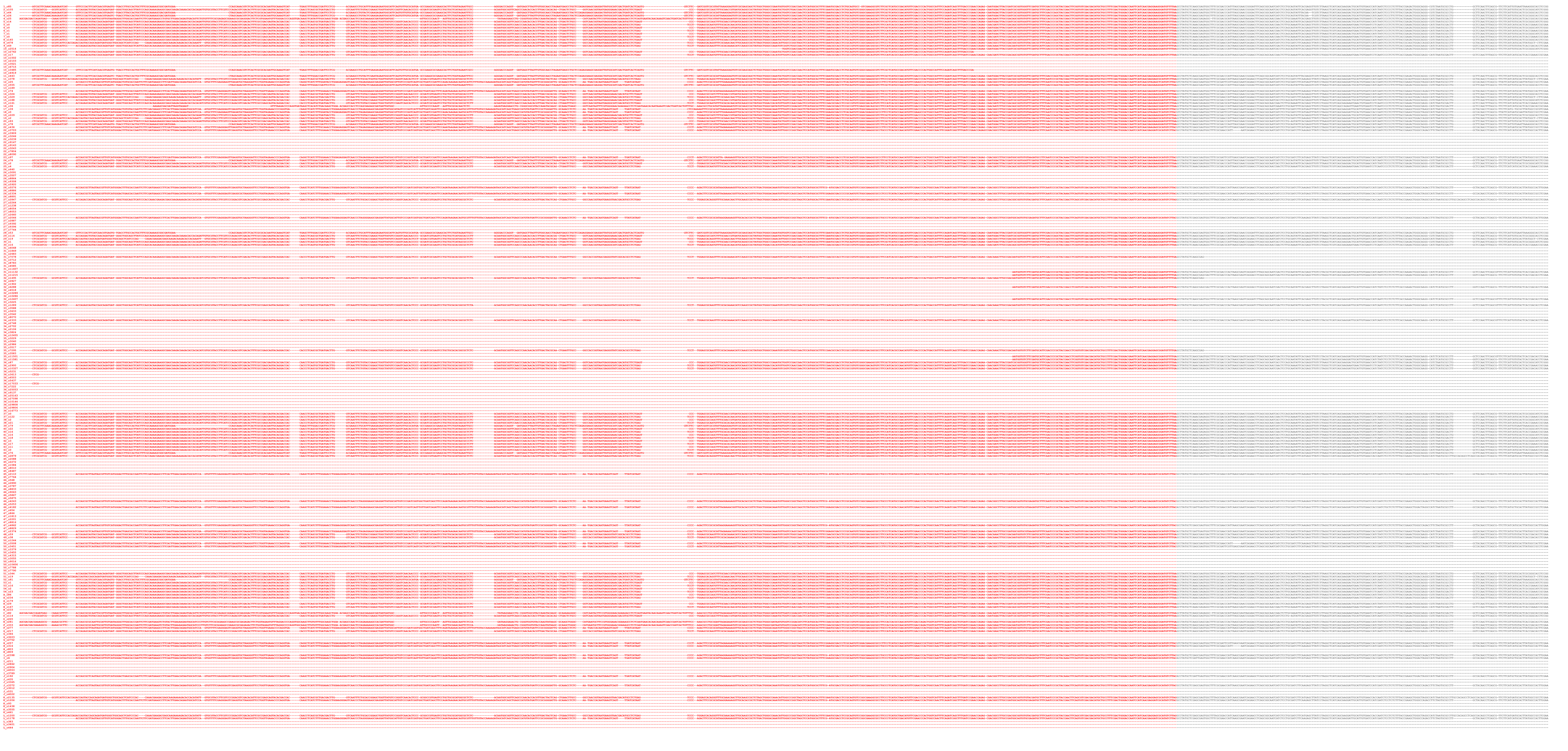

```
1_c85     CGTGCACCTTTCCCTTCCATC [illegible]
2_c105    CGTGCACCTTTCTCTTCCATC [illegible]
3_c64     CGTGTACCTTTCCCTTCCATC [illegible]
4_c26     CGTGTACCTTTCCCTTCCATC [illegible]
4_c25     TATCCAACTTCGCTTTCCACC [illegible]
5_c14     CGTGCACCTTTCTCTTCCATC [illegible]
5_c0      TATCCACCTTCCCTTTCCACC [illegible]
6_c0      CGTGCACCTTTCTCTTCCATC [illegible]
6_c1      TATCCACCTTCCCTTTCCACC [illegible]
7_c1      CGTGCACCTTTCTCTTCCATC [illegible]
7_c0      TATCCACCTTCCCTTTCCACC [illegible]
8_c145    CGTGTACCTTTCCCTTCCATC [illegible]
8_c478    TATGCCCCTTCTTTCTCCACC [illegible]
9_c69     CACCCACCTTCCCTTTCCACC [illegible]
10_c2514  CGTGCACCTTTCTCTTCCATC [illegible]
10_c2762  --------------------- [illegible]
10_c17    TATCCACCTTCCCTTTCCACC [illegible]
10_c1502  --------------------- [illegible]
10_c2103  --------------------- [illegible]
10_c3521  --------------------- [illegible]
10_c6715  --------------------- [illegible]
10_c3485  --------------------- [illegible]
10_c4919  --------------------- [illegible]
11_c443   CGTGTACCTTTCCCTTCCATC [illegible]
11_c378   TATGCCCCTTCTTTCTCCACC [illegible]
12_c25    TATACCCCTTCTTTCTCCACC [illegible]
13_c555   CGTGTACCTTTCCCTTCCATC [illegible]
13_c189   --------------------- [illegible]
13_c74    TATGCCCCTTCTTTCTCCACC [illegible]
14_c324   CGTGTACCTTTCCCTTCCATC [illegible]
14_c329   TATGCCCCTTCTTTCTCCACC [illegible]
15_c188   CGTGCACCTTTCTCTTCCATC [illegible]
15_c161   TATCCACCTTCCCTTTCCACC [illegible]
16_c36    TATCCACCTTCCCTTTCCACC [illegible]
17_c39    TATACCCCTTCTTTCTCCAAC [illegible]
18_c0     TATACCCCTTCTTTCTCCACC [illegible]
19_c229   CGTGTACCTTTCCCTTCCATC [illegible]
19_c2     TATGCCCCTTCTTTCTCCACC [illegible]
20_c93    CGTGCACCTTTCCCTTCCATC [illegible]
21_c78    CGTGCACCTTTCCCTTCCATC [illegible]
22_c436   TATACCTCTTCTTTCTCCACC [illegible]
22_c3703  TATGCCCCTTCTTTCTCCACC [illegible]
22_c3909  --------------------- [illegible]
22_c3246  --------------------- [illegible]
22_c3239  --------------------- [illegible]
22_c9147  --------------------- [illegible]
22_c9143  --------------------- [illegible]
22_c3243  --------------------- [illegible]
22_c7804  --------------------- [illegible]
22_c8090  --------------------- [illegible]
23_c97    TATACCTCTTCTTTCTCCACC [illegible]
24_c78    CGTGCACCTTTCCCTTCCATC [illegible]
25_c2     CGTGCACCTTTCTCTTCCATC [illegible]
25_c4     TATCCACCTTCCCTTTCCACC [illegible]
26_c640   --------------------- [illegible]
26_c3091  --------------------- [illegible]
26_c3507  --------------------- [illegible]
26_c8864  --------------------- [illegible]
26_c9308  --------------------- [illegible]
26_c5920  --------------------- [illegible]
26_c5576  TATGCCCCTTCTTTCTCCACC [illegible]
26_c8886  --------------------- [illegible]
26_c2334  AATGCCCCTTCTTTCTCCACC [illegible]
26_c8891  --------------------- [illegible]
27_c2847  TATACCCCTTCTTTCTCCACC [illegible]
27_c2264  --------------------- [illegible]
27_c1238  --------------------- [illegible]
27_c8054  --------------------- [illegible]
27_c8053  --------------------- [illegible]
27_c2660  --------------------- [illegible]
27_c2066  TATGCCCCTTCTTTCTCCACC [illegible]
27_c2887  --------------------- [illegible]
27_c9392  --------------------- [illegible]
27_c9396  --------------------- [illegible]
27_c7764  --------------------- [illegible]
28_c53    CGTGCACCTTTCCCTTCCATC [illegible]
29_c114   CGTGTACCTTTCCCTTCCATC [illegible]
29_c19    TATGCCCCTTCTTTCTCCACC [illegible]
30_c1     CGTGCACCTTTCTCTTCCATC [illegible]
30_c16    TATCCACCTTCCCTTTCCACC [illegible]
31_c6068  --------------------- [illegible]
31_c1849  --------------------- [illegible]
31_c2683  --------------------- [illegible]
31_c7078  --------------------- [illegible]
31_c3712  --------------------- [illegible]
31_c8783  --------------------- [illegible]
31_c4585  --------------------- [illegible]
31_c11607 --------------------- [illegible]
31_c11132 TACCCACCTTCCCTTTCCACC [illegible]
31_c11215 CACCCACCTTCCCTTTCCACC [illegible]
32_c1495  --------------------- [illegible]
32_c2827  --------------------- [illegible]
32_c1902  --------------------- [illegible]
32_c6288  CACCCACCTTCCCTTTCCACC [illegible]
32_c4518  --------------------- [illegible]
32_c10289 --------------------- [illegible]
32_c10286 --------------------- [illegible]
32_c12407 TACCCACCTTCCCTTTCCACC [illegible]
33_c3225  --------------------- [illegible]
33_c1289  TACCCACCTTCCCTTTCCACC [illegible]
33_c3588  --------------------- [illegible]
33_c3633  --------------------- [illegible]
33_c3542  --------------------- [illegible]
33_c8070  --------------------- [illegible]
34_c2333  TACCCACCTTCCCTTTCCACC [illegible]
34_c2748  --------------------- [illegible]
34_c2702  --------------------- [illegible]
34_c2106  --------------------- [illegible]
34_c3824  --------------------- [illegible]
34_c12455 --------------------- [illegible]
35_c3229  --------------------- [illegible]
35_c3040  --------------------- [illegible]
35_c2986  --------------------- [illegible]
35_c3517  --------------------- [illegible]
35_c7295  --------------------- [illegible]
35_c3583  --------------------- [illegible]
35_c11501 TACCCACCTTCCCTTTCCACC [illegible]
35_c11581 CACCCACCTTCCCTTTCCACC [illegible]
36_c87    CGTGCACCTTTCTCTTCCATC [illegible]
36_c2900  TATCCACCTTCCCTTTCCACC [illegible]
36_c10327 CACCCACCTTCCCTTTCCACC [illegible]
36_c5231  --------------------- [illegible]
36_c3613  --------------------- [illegible]
36_c6503  --------------------- [illegible]
36_c5437  --------------------- [illegible]
36_c17033 --------------------- [illegible]
36_c7222  --------------------- [illegible]
36_c25053 --------------------- [illegible]
36_c9137  --------------------- [illegible]
36_c25163 --------------------- [illegible]
36_c27098 --------------------- [illegible]
36_c10144 --------------------- [illegible]
36_c29808 --------------------- [illegible]
36_c29806 --------------------- [illegible]
36_c16773 --------------------- [illegible]
37_c0     CGTGCACCTTTCTCTTCCATC [illegible]
37_c122   TATCCACCTTCCCTTTCCACC [illegible]
38_c2     CGTGCACCTTTCTCTTCCATC [illegible]
38_c21    TATCCACCTTCCCTTTCCACC [illegible]
39_c79    CGTGCACCTTTCCCTTCCATC [illegible]
40_c22    CGTGCACCTTTCTCTTCCATC [illegible]
40_c16    TATCCACCTTCCCTTTCCACC [illegible]
41_c16    CGTGCACCTTTCTCTTCCATC [illegible]
41_c18    TATCCACCTTCCCTTTCCACC [illegible]
42_c3     CGTGCACCTTTCTCTTCCATC [illegible]
42_c17    TATCCACCTTCCCTTTCCACC [illegible]
43_c11    CGTGCACCTTTCTCTTCCATC [illegible]
43_c0     TATCCACCTTCCCTTTCCACC [illegible]
44_c76    CGTGCACCTTTCCCTTCCATC [illegible]
45_c2879  TATACCCCTTCTTTCTCCACC [illegible]
45_c2088  --------------------- [illegible]
45_c2272  --------------------- [illegible]
45_c1584  --------------------- [illegible]
45_c8010  --------------------- [illegible]
45_c8487  --------------------- [illegible]
45_c2110  TATGCCCCTTCTTTCTCCACC [illegible]
45_c7764  --------------------- [illegible]
46_c568   --------------------- [illegible]
46_c2890  --------------------- [illegible]
46_c3787  --------------------- [illegible]
46_c8833  --------------------- [illegible]
46_c9063  --------------------- [illegible]
46_c5867  --------------------- [illegible]
46_c5875  --------------------- [illegible]
46_c4243  TATGCCCCTTCTTTCTCCACC [illegible]
46_c5253  --------------------- [illegible]
46_c4182  AATGCCCCTTCTTTCTCCACC [illegible]
47_c654   --------------------- [illegible]
47_c866   --------------------- [illegible]
47_c1813  --------------------- [illegible]
47_c2201  --------------------- [illegible]
47_c8816  --------------------- [illegible]
47_c4862  TATGCCCCTTCTTTCTCCACC [illegible]
47_c8829  --------------------- [illegible]
47_c2374  AATGCCCCTTCTTTCTCCACC [illegible]
48_c59    CACCCACCTTCCCTTTCCACC [illegible]
49_c38    CACCCACCTTCCCTTTCCACC [illegible]
50_c1244  --------------------- [illegible]
50_c3508  TATGCCCCTTCTTTCTCCACC [illegible]
50_c3451  TATACCTCTTCTTTCTCCACC [illegible]
50_c3379  --------------------- [illegible]
50_c3455  --------------------- [illegible]
50_c4315  --------------------- [illegible]
50_c3055  --------------------- [illegible]
50_c9016  --------------------- [illegible]
50_c10606 --------------------- [illegible]
50_c7788  --------------------- [illegible]
50_c7790  --------------------- [illegible]
51_c344   CGTGTACCTTTCCCTTCCATC [illegible]
51_c19    TATGCCCCTTCTTTCTCCACC [illegible]
52_c81    CGTGCACCTTTCCCTTCCATC [illegible]
53_c1     CGTGCACCTTTCTCTTCCATC [illegible]
53_c16    TATCCACCTTCCCTTTCCACC [illegible]
54_c2     CGTGCACCTTTCTCTTCCATC [illegible]
54_c23    TATCCACCTTCCCTTTCCACC [illegible]
A_c86     CGTGCACCTTTCTCTTCCATC [illegible]
A_c234    TATCCACCTTCCCTTTCCACC [illegible]
A_c179    --------------------- [illegible]
B_c29     CGTGCACCTTTCTCTTCCATC [illegible]
B_c167    TATCCACCTTCCCTTTCCACC [illegible]
B_c233    --------------------- [illegible]
C_c82     TATCCAACTTCGCTTTCCACC [illegible]
D_c114    CGTGCACCTTTCCCTTCCATC [illegible]
D_c220    --------------------- [illegible]
E_c281    CACCCACCTTCCCTTTCCACC [illegible]
F_c333    CACCCACCTTCCCTTTCCACC [illegible]
F_c277    TATGCCCCTTCTTTCTCCACC [illegible]
G_c217    CACCCACCTTCCCTTTCCACC [illegible]
G_c346    --------------------- [illegible]
G_c3223   --------------------- [illegible]
G_c2752   --------------------- [illegible]
H_c257    TATACCTCTTCTTTCTCCACC [illegible]
H_c316    TATGCCCCTTCTTTCTCCACC [illegible]
H_c803    --------------------- [illegible]
H_c809    --------------------- [illegible]
I_c2398   TATGCCCCTTCTTTCTCCACC [illegible]
I_c6      AATGCCCCTTCTTTCTCCACC [illegible]
I_c211    --------------------- [illegible]
I_c2862   --------------------- [illegible]
I_c2904   --------------------- [illegible]
I_c4553   --------------------- [illegible]
I_c3789   --------------------- [illegible]
J_c192    AATGCCCCTTCTTTCTCCACC [illegible]
J_c435    --------------------- [illegible]
J_c3365   --------------------- [illegible]
J_c3372   TATGCCCCTTCTTTCTCCACC [illegible]
J_c3012   --------------------- [illegible]
J_c531    --------------------- [illegible]
J_c4022   --------------------- [illegible]
K_c2135   TATACCCCTTCTTTCTCCACC [illegible]
K_c1206   TATGCCCCTTCTTTCTCCACC [illegible]
K_c93     --------------------- [illegible]
K_c1438   --------------------- [illegible]
K_c3555   --------------------- [illegible]
K_c461    --------------------- [illegible]
L_c1200   TATACCCCTTCTTTCTCCACC [illegible]
L_c1178   TATGCCCCTTCTTTCTCCACC [illegible]
L_c383    --------------------- [illegible]
L_c3401   --------------------- [illegible]
L_c444    --------------------- [illegible]
```

pleckstrin homology domain protein

```
1_c85      CACCGCAAAATGCGTGCCCATGACCGCCATGCCGGATAATGCC-ATTCACTACGTTCGCCTTGACGTACTTGCTCGGCGGTTTAACCTTCCTGCCATTGTTGTTGCTCGCGCTATTGACTCCAGCTTGGTTACTACTTCCTGAAAGAAGAGG[illegible]
2_c105     CACCACAAAATGCGTGCCCATGACCGCCATGCCGAATAATGCC-ATTCACTACGTTCGCCTTGACGTATTTGCTCGGCGGTTTAACCTTCCTGCCATTGTTGTTGCTCACGCTATTGACTCCAGCTTGGTTACTACTTCCTGAAAGAAGAGG[illegible]
3_c64      CACCGCAAAATGCGTGCCCATGACCGCCATGCCGGATAATGCC-ATTCACTACGTTCGCCTTGACGTATTTGCTCGGCGGTTTAACCTTCCTGCCATTGTTGTTGCTCGCGCTATTGACTCCAGCTTGGTTGCTACTTCCTGAAAGAAGAG[illegible]
4_c26      CACCGCAAAATGCGTGCCCATGACCGCCATGCCGGATAATGCC-ATTCACTACGTTCGCCTTGACGTATTTGCTCGGCGGTTTAACCTTCCTGCCATTGTTGTTGCTCGCGCTATTGACTCCAGCTTGGTTACTACTTCCTGAAAGAAGAG[illegible]
4_c25      CACCACAATATGCGTGCCCATGACCGCCATGCCGGATAATGCC-ATTCACTACGCTCGCCTTCACGTATTTGCTCGGTGGTTTAACTTTCCTGCCACTGTTGTTGCTCACGTTATTGACGCCAGCTTGGTTACTACTTCCTCAAAAAACGGG[illegible]
5_c14      CACCACAAAATGCGTGCCCATGACCGCCATGCCGAATAATGCC-ATTCACTACGTTCGCCTTGACGTATTTGCTCGGCGGTTTAACCTTCCTGCCATTGTTGTTGCTCACGCTATTGACTCCAGCTTGGTTACTACTTCCTGAAAGAAGAGG[illegible]
5_c0       CACCACAATATGCGTGCCCATGACCGCCATGCCGGATAATGCC-ATTCACTACGCTCGCCTTCACGTATTTGCTCGGCGGTTTAACTTTCCTGCCACTGTTGTTGCTCACGTTATTGACGCCAGCTTGGTTACTACTTCCTCAAAAACCGGG[illegible]
6_c0       CACCACAAAATGCGTGCCCATGACCGCCATGCCGAATAATGCC-ATTCACTACGTTCGCCTTGACGTATTTGCTCGGCGGTTTAACCTTCCTGCCATTGTTGTTGCTCACGCTATTGACTCCAGCTTGGTTACTACTTCCTGAAAGAAGAGG[illegible]
6_c1       CACCACAATATGCGTGCCCATGACCGCCATGCCGGATAATGCC-ATTCACTACGCTCGCCTTCACGTATTTGCTCGGCGGTTTAACTTTCCTGCCACTGTTGTTGCTCACGTTATTGACGCCAGCTTGGTTACTACTTCCTCAAAAACCGGG[illegible]
7_c1       CACCACAAAATGCGTGCCCATGACCGCCATGCCGAATAATGCC-ATTCACTACGTTCGCCTTGACGTATTTGCTCGGCGGTTTAACCTTCCTGCCATTGTTGTTGCTCACGCTATTGACTCCAGCTTGGTTACTACTTCCTGAAAGAAGAGG[illegible]
7_c0       CACCACAATATGCGTGCCCATGACCGCCATGCCGGATAATGCC-ATTCACTACGCTCGCCTTCACGTATTTGCTCGGCGGTTTAACTTTCCTGCCACTGTTGTTGCTCACGTTATTGACGCCAGCTTGGTTACTACTTCCTCAAAAACCGGG[illegible]
8_c145     CACCGCAAAATGCGTGCCCATGACCGCCATGCCGGATAATGCC-ATTCACTACGTTCGCCTTGACGTATTTGCTCGGCGGTTTAACCTTCCTGCCATTGTTGTTGCTCGCGCTATTGACTCCAGCTTGGTTACTACTTCCTGAAAGAAGAGG[illegible]
8_c478     TACCACAAAATGCGCGCCCATGACCGCCATGCCGGATAATGCCAATTCATTACGTTCGCCGTGACGTATTTGCTCGGCGGTTTAACCTTCCTGCCCCTGTTGTTGCTCACGCTATTAACGCCCGCTTGGTTTCTACTTCCTGAAGGGAAACG[illegible]
9_c69      CACCACAAAATGCGCGCCCACGACCGCCATGCCGGATAATGCC-ATTCACTACGTTCGCCTTCACGTATTTGCTCGGCGGTTTAACTTTCCTGCCACTGTTGTTGCTTACGATATTGACGCCAGCTTGGTTACTACTTCCTGAAAGAACAGG[illegible]
10_c2514   CACCACAAAATGCGTGCCCATGACCGCCATGCCGAATAATGCC-ATTCACTACGTTCGCCTTGACGTATTTGCTCGGCGGTTTAACCTTCCTGCCATTGTTGTTGCTCACGCTACTGACTCCAGCTTGGTTACTACTTCCTGAAAGAAGAGG[illegible]
10_c2762   [illegible]
10_c17     CACCACAATATGCGTGCCCATGACCGCCATGCCGGATAATGCC-ATTCACTACGCTCGCCTTCACGTATTTGCTCGGCGGTTTAACTTTCCTGCCACTGTTGTTGCTCACGTTATTGACGCCAGCTTGGTTACTACTTCCTCAAAAACCGGG[illegible]
10_c1502   [illegible]
10_c2103   [illegible]
10_c3521   [illegible]
10_c6715   [illegible]
10_c3485   [illegible]
10_c4919   [illegible]
11_c443    CACCGCAAAATGCGTGCCCATGACCGCCATGCCGGATAATGCC-ATTCACTACGTTCGCCTTGACGTATTTGCTCGGCGGTTTAACCTTCCTGCCATTGTTGTTGCTCGCGCTATTGACTCCAGCTTGGTTACTACTTCCTGAAAGAAGTTC[illegible]
11_c378    TACCACAAAATGCGCGCCCATGACCGCCATGCCGGATAATGTC-ATTCATTACGTTCGCCGTGACGTATTTGCTCGGCGGTTTAACCTTCCTGCCCCTGTTGTTGCTCACGCTATTGACGCCTGCTTGGTTTCTCCTTCCTGAAGGAAAACG[illegible]
12_c25     CACCACAAAATGCGCGCCCATGACCGCCATGCCGGATAATGCC-ATTCATTACGTTCGCCGTGACGTATTTGCTCGGCGGTTTAACCTTCCTGCCCCTGTTGTTGCTCACGCTATTAACGCCCGCTTGGTTTCTACTTCCTGAAGGGCAACG[illegible]
13_c555    CACCGCAAAATGCGTGCCCATGACCGCCATGCCGGATAATGCC-ATTCACTACGTTCGCCTTGACGTATTTGCTCGGCGGTTTAACCTTCCTGCCATTGTTGTTGCTCGCGCTATTGACTCCAGCTTGGTTACTACTTCCTGAAAGAAGAG[illegible]
13_c189    [illegible]
13_c74     TACCACAAAATGCGCGCCCATGACTGCCATGCCGGATAATGCC-ATTCATTACGTTCGCCGTGACGTATTTGCTCGGCGGATTAACCTTCCTGCCCCTGTTGTTGCTCACGCTATTAACGCCCGCTTGGTTTCTACTTCCTGAACGAAAACG[illegible]
14_c324    CACCGCAAAATGCGTGCCCATGACCGCCATGCCGGATAATGCC-ATTCACTACGTTCGCCTTGACGTATTTGCTCGGCGGTTTAACCTTCCTGCCATTGTTGTTGCTCGCGCTATTGACTCCAGCTTGGTTACTACTTCCTGAAAGAAGAGG[illegible]
14_c329    TACCACAAAATGCGCGCCC[illegible]
15_c188    CACCGCAAAATGCGTGCCC[illegible]
15_c161    CACCACAATATGCGTGCCC[illegible]
16_c36     CACCACAATATGCGCGCCC[illegible]
17_c39     CACCATAAAATGCGCGCCC[illegible]
18_c0      CACCACAAAATGCGCGCCC[illegible]
19_c229    CACCGCAAAATGCGTGCCC[illegible]
19_c2      TACCACAAAATGCGCGCCC[illegible]
20_c93     CACCGCAAAATGCGTGCCC[illegible]
21_c78     CACCGCAAAATGCGTGCCC[illegible]
22_c436    CACCACAAAATGCGCGCCC[illegible]
22_c3703   TACCACAAAATGCGCGCCC[illegible]
22_c3909   [illegible]
22_c3246   [illegible]
22_c3239   [illegible]
22_c9147   [illegible]
22_c9143   [illegible]
22_c3243   [illegible]
22_c7804   [illegible]
22_c8090   [illegible]
23_c97     CACCACAAAATGCGCGCCC[illegible]
24_c78     CACCGCAAAATGCGTGCCC[illegible]
25_c2      CACCACAAAATGCGTGCCC[illegible]
25_c4      CACCACAATATGCGTGCCC[illegible]
26_c640    [illegible]
26_c3091   [illegible]
26_c3507   [illegible]
26_c8864   [illegible]
26_c9308   [illegible]
26_c5920   [illegible]
26_c5576   TACCACAAAATGCGCGCCC[illegible]
26_c8886   [illegible]
26_c2334   CACCACAAAATGCGCGCCC[illegible]
26_c8891   [illegible]
27_c2847   CACCACAAAATGCGCGCCC[illegible]
27_c2264   [illegible]
27_c1238   [illegible]
27_c8054   [illegible]
27_c8053   [illegible]
27_c2660   [illegible]
27_c2066   TACCACAAAATGCGCGCCC[illegible]
27_c2887   [illegible]
27_c9392   [illegible]
27_c9396   [illegible]
27_c7764   [illegible]
28_c53     CACCGCAAAATGCGTGCCC[illegible]
29_c114    CACCGCAAAATGCGTGCCC[illegible]
29_c19     TACCACAAAATGCGCGCCC[illegible]
30_c1      CACCACAAAATGCGTGCCC[illegible]
30_c16     CACCACAATATGCGTGCCC[illegible]
31_c6068   CACCACAAAATGCGCGCCC[illegible]
31_c1849   [illegible]
31_c2683   [illegible]
31_c7078   [illegible]
31_c3712   [illegible]
31_c8783   [illegible]
31_c4585   [illegible]
31_c11607  [illegible]
31_c11132  [illegible]
31_c11215  [illegible]
32_c1495   [illegible]
32_c2827   [illegible]
32_c1902   [illegible]
32_c6288   CACCACAAAATGCGCGCCC[illegible]
32_c4518   [illegible]
32_c10289  [illegible]
32_c10286  [illegible]
32_c12407  [illegible]
33_c3225   CACCACAAAATGCGCGCCC[illegible]
33_c1289   [illegible]
33_c3588   [illegible]
33_c3633   [illegible]
33_c3542   [illegible]
33_c8070   [illegible]
34_c2333   CACCACAAAATGCGCGCCC[illegible]
34_c2748   [illegible]
34_c2702   [illegible]
34_c2106   [illegible]
34_c3824   [illegible]
34_c12455  [illegible]
35_c3229   CACCACAAAATGCGCGCCC[illegible]
35_c3040   [illegible]
35_c2986   [illegible]
35_c3517   [illegible]
35_c7295   [illegible]
35_c3583   [illegible]
35_c11501  [illegible]
35_c11581  [illegible]
36_c87     CACCACAAAATGCGTGCCC[illegible]
36_c2900   CACCACAATATGCGTGCCC[illegible]
36_c10327  CACCACAAAATGCGCGCCC[illegible]
36_c5231   [illegible]
36_c3613   [illegible]
36_c6503   [illegible]
36_c5437   [illegible]
36_c17033  [illegible]
36_c7222   [illegible]
36_c25053  [illegible]
36_c9137   [illegible]
36_c25163  [illegible]
36_c27098  [illegible]
36_c10144  [illegible]
36_c29808  [illegible]
36_c29806  [illegible]
36_c16773  [illegible]
37_c0      CACCACAAAATGCGTGCCC[illegible]
37_c122    CACCACAATATGCGTGCCC[illegible]
38_c2      CACCACAAAATGCGTGCCC[illegible]
38_c21     CACCACAATATGCGTGCCC[illegible]
39_c79     CACCGCAAAATGCGTGCCC[illegible]
40_c22     CACCACAAAATGCGTGCCC[illegible]
40_c16     CACCACAATATGCGTGCCC[illegible]
41_c16     CACCACAAAATGCGTGCCC[illegible]
41_c18     CACCACAATATGCGTGCCC[illegible]
42_c3      CACCACAAAATGCGTGCCC[illegible]
42_c17     CACCACAATATGCGTGCCC[illegible]
43_c11     CACCACAAAATGCGTGCCC[illegible]
43_c0      CACCACAATATGCGTGCCC[illegible]
44_c76     CACCGCAAAATGCGTGCCC[illegible]
45_c2879   CACCACAAAATGCGCGCCC[illegible]
45_c2088   [illegible]
45_c2272   [illegible]
45_c1584   [illegible]
45_c8010   [illegible]
45_c8487   [illegible]
45_c2110   TACCACAAAATGCGCGCCC[illegible]
45_c7764   [illegible]
46_c568    [illegible]
46_c2890   [illegible]
46_c3787   [illegible]
46_c8833   [illegible]
46_c9063   [illegible]
46_c5867   [illegible]
46_c5875   [illegible]
46_c4243   TACCACAAAATGCGCGCCC[illegible]
46_c5253   [illegible]
46_c4182   CACCACAAAATGCGCGCCC[illegible]
47_c654    [illegible]
47_c866    [illegible]
47_c1813   [illegible]
47_c2201   [illegible]
47_c8816   [illegible]
47_c4862   TACCACAAAATGCGCGCCC[illegible]
47_c8829   [illegible]
47_c2374   CACCACAAAATGCGCGCCC[illegible]
48_c59     CACCACAAAATGCGCGCCC[illegible]
49_c38     CACCACAAAATGCGCGCCC[illegible]
50_c1244   [illegible]
50_c3508   TACCACAAAATGCGCGCCC[illegible]
50_c3451   CACCACAAAATGCGCGCCC[illegible]
50_c3379   [illegible]
50_c3455   [illegible]
50_c4315   [illegible]
50_c3055   [illegible]
50_c9016   [illegible]
50_c10606  [illegible]
50_c7788   [illegible]
50_c7790   [illegible]
51_c344    CACCGCAAAATGCGTGCCC[illegible]
51_c19     TACCACAAAATGCGCGCCC[illegible]
52_c81     CACCGCAAAATGCGTGCCC[illegible]
53_c1      CACCACAAAATGCGTGCCC[illegible]
53_c16     CACCACAATATGCGTGCCC[illegible]
54_c2      CACCACAAAATGCGTGCCC[illegible]
54_c23     CACCACAATATGCGTGCCC[illegible]
A_c86      CACCACAAAATGCGTGCCC[illegible]
A_c234     CACCACAATATGCGTGCCC[illegible]
A_c179     [illegible]
B_c29      CACCACAAAATGCGTGCCC[illegible]
B_c167     CACCACAATATGCGTGCCC[illegible]
B_c233     [illegible]
C_c82      CACCACAATATGCGTGCCC[illegible]
D_c114     CACCGCAAAATGCGTGCCC[illegible]
D_c220     [illegible]
E_c281     CACCACAAAATGCGCGCCC[illegible]
F_c333     CACCACAAAATGCGCGCCC[illegible]
F_c277     TACCACAAAATGCGCGCCC[illegible]
G_c217     CACCACAAAATGCGCGCCC[illegible]
G_c346     [illegible]
G_c3223    [illegible]
G_c2752    [illegible]
H_c257     CACCACAAAATGCGCGCCC[illegible]
H_c316     TACCACAAAATGCGCGCCC[illegible]
H_c803     [illegible]
H_c809     [illegible]
I_c2398    TACCACAAAATGCGCGCCC[illegible]
I_c6       CACCACAAAATGCGCGCCC[illegible]
I_c211     [illegible]
I_c2862    [illegible]
I_c2904    [illegible]
I_c4553    [illegible]
I_c3789    [illegible]
J_c192     CACCACAAAATGCGCGCCC[illegible]
J_c435     [illegible]
J_c3365    [illegible]
J_c3372    TACCACAAAATGCGCGCCC[illegible]
J_c3012    [illegible]
J_c531     [illegible]
J_c4022    [illegible]
K_c2135    CACCACAAAATGCGCGCCC[illegible]
K_c1206    TACCACAAAATGCGCGCCC[illegible]
K_c93      [illegible]
K_c1438    [illegible]
K_c3555    [illegible]
K_c461     [illegible]
L_c1200    CACCACAAAATGCGCGCCC[illegible]
L_c1178    TACCACAAAATGCGCGCCC[illegible]
L_c383     [illegible]
L_c3401    [illegible]
L_c444     [illegible]
```

```
1_c85     TTTCAACGCTTTGGTAGGCCGAATCTTCCTCGGTATACACCGTACGACCTTTTTGGAGACTCTTATCCGGACAAAGATTGAACGCAAAATAGCGCGGGTCCAGAAACCGGCATTCATTGCCTCATTGGATGTGAAATCCATAAGTCTTGGCA[illegible]
2_c105    TTTCAACGCTTTGGTAGGCCGAATCTTCCTCGGTATACACCGTACGACCTTTTTGGAGACTCTTATCCGGACAAAGATTGAACGCAAAATAGCGCGGGTCCAGAAACCGGCATTCATTGCCTCATTGGATGTGAAATCCATAAGTCTTGGCA[illegible]
3_c64     TTTCAACGCTTTGGTAGGCCGAATCTTCCTCGGTATACACCGTACGACCTTTTTGGAGACTCTTATCCGGACAAAGATTGAACGCAAAATAGCGCGGGTCCAGAAACCGGCATTCATTGCCTCATTGGATGTGAAATCCATAAGTCTTGGCA[illegible]
4_c26     TTTCAACGCTTTGGTAGGCCGAATCTTCCTCGGTATACACCGTACGACCTTTTTGGAGACTCTTATCCGGACAAAGATTGAACGCAAAATAGCGCGGGTCCAGAAACCGGCATTCATTGCCTCATTGGATGTGAAATCCATAAGTCTTGGCA[illegible]
4_c25     TTTCAACGCTTTGGTAGGCCGGATATTCCTCGGTATACACCGTACGACCTTTTTGGAGACCCTCATCCGGACAAAGATTGAACGTAAAATAGCAAGGGTCCAGAAACCGGCATTCATTGCCTCCTTGGACGTGAAGTCCATAAGTCTTGGCA[illegible]
5_c14     TTTCAACGCTTTGGTAGGCCGAATCTTCCTCGGTATACACCGTACGACCTTTTTGGAGACTCTTATCCGGACAAAGATTGAACGCAAAATAGCGCGGGTCCAGAAACCGGCATTCATTGCCTCATTGGATGTGAAATCCATAAGTCTTGGCA[illegible]
5_c0      TTTCAACGCTTTGGTAGGCCGAATCTTCCTCGGTATACACCGTACGACCTTTCTGGAGACCCTTATCCGGACAAAGATTGAGCGTAAAATAGCGAGGGTCCAGAAACCGGCATTCATTGCCTCCTTGGACGTGAAGTCCATAAGTCTTGGCA[illegible]
6_c0      TTTCAACGCTTTGGTAGGCCGAATCTTCCTCGGTATACACCGTACGACCTTTTTGGAGACTCTTATCCGGACAAAGATTGAACGCAAAATAGCGCGGGTCCAGAAACCGGCATTCATTGCCTCATTGGATGTGAAATCCATAAGTCTTGGCA[illegible]
6_c1      TTTCAACGCTTTGGTAGGCCGAATCTTCCTCGGTATACACCGTACGACCTTTCTGGAGACCCTTATCCGGACAAAGATTGAGCGTAAAATAGCGAGGGTCCAGAAACCGGCATTCATTGCCTCCTTGGACGTGAAGTCCATAAGTCTTGGCA[illegible]
7_c1      TTTCAACGCTTTGGTAGGCCGAATCTTCCTCGGTATACACCGTACGACCTTTTTGGAGACTCTTATCCGGACAAAGATTGAACGCAAAATAGCGCGGGTCCAGAAACCGGCATTCATTGCCTCATTGGATGTGAAATCCATAAGTCTTGGCA[illegible]
7_c0      TTTCAACGCTTTGGTAGGCCGAATCTTCCTCGGTATACACCGTACGACCTTTCTGGAGACCCTTATCCGGACAAAGATTGAGCGTAAAATAGCGAGGGTCCAGAAACCGGCATTCATTGCCTCCTTGGACGTGAAGTCCATAAGTCTTGGCA[illegible]
8_c145    TTTCAACGCTTTGGTAGGCCGAATCTTTCTCGGTATACACCGTACGACCTTTTTGGAGACTCTTATCCGGACAAAGATTGAACGCAAAATAGCGCGGGTCCAGAAACCGGCATTCATTGCCTCATTGGATGTGAAATCCATAAGTCTTGGCA[illegible]
8_c478    CTTCAACGCTTTGATAGGCCGAATCTTCCTTGGAATCCACCGGACAACTTTTCTGGAGACTCTCATCCGGACAAAGATTGAACGTAAAATAGCGCGGGTCCAGAAACCGGCGTTCATTGCCTCCTTGGACGTGAAGTCCATAAGTCTTGGTA[illegible]
9_c69     TTTCAACGCTTTGGTAGGCCGGATCTTCCTCGGTATACACCGTACGACCTTTTTGGAGACCCTCATCCGGACAAAGATTGAACGTAAAATAGCAAGGGTCCAGAAACCGGCATTCATTGCCTCCTTGGACGTGAAGTCCATAAGTCTTGGCA[illegible]
10_c2514  TTTCAACGCTTTGGTAGGCCGAATCTTCCTCGGTATACACCGTACGACCTTTTTGGAGACTCTTATCCGGACAAAGATTGAACGCAAAATAGCGCGGGTCCAGAAACCGGCATTCATTGCCTCATTGGATGTGAAATCCATAAGTCTTGGCA[illegible]
10_c2762  [illegible]
10_c17    TTTCAACGCTTTGGTAGGCCGAATCTTCCTCGGTATACACCGTACGACCTTTCTGGAGACCCTTATCCGGACAAAGATTGAGCGTAAAATAGCGAGGGTCCAGAAACCGGCATTCATTGCCTCCTTGGACGTGAAGTCCATAAGTCTTGGCA[illegible]
10_c1502  [illegible]
10_c2103  [illegible]
10_c3521  [illegible]
10_c6715  [illegible]
10_c3485  [illegible]
10_c4919  [illegible]
11_c443   TTTCAACGCTTTGGTAGGCCGAATCTTCCTCGGTATACACCGTACGACCTTTTTGGAGACTCTTATCCGGACAAAGATTGAACGCAAAATAGCGCGGGTCCAGAAACCGGCATTCATTGCCTCATTGGATGTGAAATCCATAAGTCTTGGCA[illegible]
11_c378   CTTCAACGCTTTGATAGGCCGAATCTTCCTTGGAATCCACCGGACAACTTTTCTGGAGACTCTCATCCGGACAAAGATTGAACGCAAAATAGCGCGGGTCCAGAAACCGGCATTCATTGCCTCCTTGGACGTGAAGTCCATAAGTCTTGGTA[illegible]
12_c25    GTTCAACGCTTTGGTAGGCCGAATCTTCCTTGGCATCCACCGAACAACCTTTCTGGAGACTCTCATCCGGACAAAGATTGAACGCAAAATAGCGCGGGTCCAGAAACCGGCGTTCATTGCCTCCTTGGACGTGAAGTCCATAAGTCTTGGCA[illegible]
13_c555   TTTCAACGCTTTGGTAGGCCGAATCTTCCTCGGTATACACCGTACGACCTTTTTGGAGACTCTTATCCGGACAAAGATTGAACGCAAAATAGCGCGGGTCCAGAAACCGGCATTCATTGCCTCATTGGATGTGAAATCCATAAGTCTTGGCA[illegible]
13_c189   [illegible]
13_c74    CTTCAACGCTTTGATAGGCCGAATCTTCCTTGGAATCCACCGGACAACTTTTCTGGAGACTCTCATCCGGACAAAGATTGAACGCAAAATAGCGCGGGTCCAGAAACCGGCGTTCATTGCCTCCTTGGACGTGAAGTCCATAAGTCTTGGTA[illegible]
14_c324   TTTCAACGCTTTGGTAGGCCGAATCTTTCTCGGTATACACCGTACGACCTTTTTGGAGACTCTTATCCGGACAAAGATTGAACGCAAAATAGCGCGGGTCCAGAAACCGGCATTCATTGCCTCATTGGATGTGAAATCCATAAGTCTTGGCA[illegible]
14_c329   CTTCAACGCTTTGATAGGCCGAATCTTCCTTGGAATCCACCGGACAACTTTTCTGGAGACTCTCATCCGGACAAAGATTGAACGTAAAATAGCGCGGGTCCAGAAACCGGCGTTCATTGCCTCCTTGGACGTGAAGTCCATAAGTCTTGGTA[illegible]
15_c188   TTTCAACGCTTTGGTAGGCCGAATCTTCCTCGGTATACACCGTACGACCTTTTTGGAGACTCTTATCCGGACAAAGATTGAACGCAAAATAGCGCGGGTCCAGAAACCGGCATTCATTGCCTCATTGGATGTGAAATCCATAAGTCTTGGCA[illegible]
15_c161   [illegible]
16_c36    [illegible]
17_c39    [illegible]
18_c0     [illegible]
19_c229   [illegible]
19_c2     [illegible]
20_c93    [illegible]
21_c78    [illegible]
22_c436   [illegible]
22_c3703  [illegible]
22_c3909  [illegible]
22_c3246  [illegible]
22_c3239  [illegible]
22_c9147  [illegible]
22_c9143  [illegible]
22_c3243  [illegible]
22_c7804  [illegible]
22_c8090  [illegible]
23_c97    [illegible]
24_c78    [illegible]
25_c2     [illegible]
25_c4     [illegible]
26_c640   [illegible]
26_c3091  [illegible]
26_c3507  [illegible]
26_c8864  [illegible]
26_c9308  [illegible]
26_c5920  [illegible]
26_c5576  [illegible]
26_c8886  [illegible]
26_c2334  [illegible]
26_c8891  [illegible]
27_c2847  [illegible]
27_c2264  [illegible]
27_c1238  [illegible]
27_c8054  [illegible]
27_c8053  [illegible]
27_c2660  [illegible]
27_c2066  [illegible]
27_c2887  [illegible]
27_c9392  [illegible]
27_c9396  [illegible]
27_c7764  [illegible]
28_c53    [illegible]
29_c114   [illegible]
29_c19    [illegible]
30_c1     [illegible]
30_c16    [illegible]
31_c6068  [illegible]
31_c1849  [illegible]
31_c2683  [illegible]
31_c7078  [illegible]
31_c3712  [illegible]
31_c8783  [illegible]
31_c4585  [illegible]
31_c11607 [illegible]
31_c11132 [illegible]
31_c11215 [illegible]
32_c1495  [illegible]
32_c2827  [illegible]
32_c1902  [illegible]
32_c6288  [illegible]
32_c4518  [illegible]
32_c10289 [illegible]
32_c10286 [illegible]
32_c12407 [illegible]
33_c3225  [illegible]
33_c1289  [illegible]
33_c3588  [illegible]
33_c3633  [illegible]
33_c3542  [illegible]
33_c8070  [illegible]
34_c2333  [illegible]
34_c2748  [illegible]
34_c2702  [illegible]
34_c2106  [illegible]
34_c3824  [illegible]
34_c12455 [illegible]
35_c3229  [illegible]
35_c3040  [illegible]
35_c2986  [illegible]
35_c3517  [illegible]
35_c7295  [illegible]
35_c3583  [illegible]
35_c11501 [illegible]
35_c11581 [illegible]
36_c87    [illegible]
36_c2900  [illegible]
36_c10327 [illegible]
36_c5231  [illegible]
36_c3613  [illegible]
36_c6503  [illegible]
36_c5437  [illegible]
36_c17033 [illegible]
36_c7222  [illegible]
36_c25053 [illegible]
36_c9137  [illegible]
36_c25163 [illegible]
36_c27098 [illegible]
36_c10144 [illegible]
36_c29808 [illegible]
36_c29806 [illegible]
36_c16773 [illegible]
37_c0     [illegible]
37_c122   [illegible]
38_c2     [illegible]
38_c21    [illegible]
39_c79    [illegible]
40_c22    [illegible]
40_c16    [illegible]
41_c16    [illegible]
41_c18    [illegible]
42_c3     [illegible]
42_c17    [illegible]
43_c11    [illegible]
43_c0     [illegible]
44_c76    [illegible]
45_c2879  [illegible]
45_c2088  [illegible]
45_c2272  [illegible]
45_c1584  [illegible]
45_c8010  [illegible]
45_c8487  [illegible]
45_c2110  [illegible]
45_c7764  [illegible]
46_c568   [illegible]
46_c2890  [illegible]
46_c3787  [illegible]
46_c8833  [illegible]
46_c9063  [illegible]
46_c5867  [illegible]
46_c5875  [illegible]
46_c4243  [illegible]
46_c5253  [illegible]
46_c4182  [illegible]
47_c654   [illegible]
47_c866   [illegible]
47_c1813  [illegible]
47_c2201  [illegible]
47_c8816  [illegible]
47_c4862  [illegible]
47_c8829  [illegible]
47_c2374  [illegible]
48_c59    [illegible]
49_c38    [illegible]
50_c1244  [illegible]
50_c3508  [illegible]
50_c3451  [illegible]
50_c3379  [illegible]
50_c3455  [illegible]
50_c4315  [illegible]
50_c3055  [illegible]
50_c9016  [illegible]
50_c10606 [illegible]
50_c7788  [illegible]
50_c7790  [illegible]
51_c344   [illegible]
51_c19    [illegible]
52_c81    [illegible]
53_c1     [illegible]
53_c16    [illegible]
54_c2     [illegible]
54_c23    [illegible]
A_c86     [illegible]
A_c234    [illegible]
A_c179    [illegible]
B_c29     [illegible]
B_c167    [illegible]
B_c233    [illegible]
C_c82     [illegible]
D_c114    [illegible]
D_c220    [illegible]
E_c281    [illegible]
F_c333    [illegible]
F_c277    [illegible]
G_c217    [illegible]
G_c346    [illegible]
G_c3223   [illegible]
G_c2752   [illegible]
H_c257    [illegible]
H_c316    [illegible]
H_c803    [illegible]
H_c809    [illegible]
I_c2398   [illegible]
I_c6      [illegible]
I_c211    [illegible]
I_c2862   [illegible]
I_c2904   [illegible]
I_c4553   [illegible]
I_c3789   [illegible]
J_c192    [illegible]
J_c435    [illegible]
J_c3365   [illegible]
J_c3372   [illegible]
J_c3012   [illegible]
J_c531    [illegible]
J_c4022   [illegible]
K_c2135   [illegible]
K_c1206   [illegible]
K_c93     [illegible]
K_c1438   [illegible]
K_c3555   [illegible]
K_c461    [illegible]
L_c1200   [illegible]
L_c1178   [illegible]
L_c383    [illegible]
L_c3401   [illegible]
L_c444    [illegible]
```

```
1_c85      TGGCACTCGATGGAGCGACAGGCATGCCCGTCCGGACGGATGATGCAA [illegible]
2_c105     TGGCACTCGATGGAGCGACAGGCATGCCCGTCCGGACGGATGATGCAA [illegible]
3_c64      TGGCACTCGATGGAGCGACAGGCATGCCCGTCCGGACGGATGATGCAA [illegible]
4_c26      TGGCACTCGATGGAGCGACAGGCATGCCCGTCCGGACGGATGATGCAA [illegible]
4_c25      TGGCACTTGATGGAGCGACGGGCATGCCCGTACGTACGGATGATGCAA [illegible]
5_c14      TGGCACTCGATGGAGCGACAGGCATGCCCGTCCGGACGGATGATGCAA [illegible]
5_c0       TGGCACTTGATGGAGCGACGGGCATGCCTGTACGTACGGGTGATGCAA [illegible]
6_c0       TGGCACTCGATGGAGCGACAGGCATGCCCGTCCGGACGGATGATGCAA [illegible]
6_c1       TGGCACTTGATGGAGCGACGGGCATGCCTGTACGTACGGGTGATGCAA [illegible]
7_c1       TGGCACTCGATGGAGCGACAGGCATGCCCGTCCGGACGGATGATGCAA [illegible]
7_c0       TGGCACTTGATGGAGCGACGGGCATGCCTGTACGTACGGGTGATGCAA [illegible]
8_c145     TGGCACTCGATGGAGCGACAGGCATGCCCGTCCGGACGGATGATGCAA [illegible]
8_c478     TGGCGGTTGATGGCGCGACAGGCATGCCTGTGCGTACGAATGATGCAA [illegible]
9_c69      TGGCAGTTGATGGAGCGACGGGTATGCCCGTTCGCTCGGGTGATGCAA [illegible]
10_c2514   TGGCACTCGATGGAGCGACAGGCATGCCCGTCCGGACGGATGATGCAA [illegible]
10_c2762   ------------------------------------------------ [illegible]
10_c17     TGGCACTTGATGGAGCGACGGGCATGCCTGTACGTACGGGTGATGCAA [illegible]
10_c1502   ------------------------------------------------ [illegible]
10_c2103   ------------------------------------------------ [illegible]
10_c3521   ------------------------------------------------ [illegible]
10_c6715   ------------------------------------------------ [illegible]
10_c3485   ------------------------------------------------ [illegible]
10_c4919   ------------------------------------------------ [illegible]
11_c443    TGGCACTCGATGGAGCGACAGGCATGCCCGTCCGGACGGATGATGCAA [illegible]
11_c378    TGGCGGTTGATGGCGCGACAGGCATGCCTGTGCGTACGAATGAAGCAA [illegible]
12_c25     TGGCGGTTGATGGCGCGACAGGCATGCCTGTGCGTACCAATGATGCAA [illegible]
13_c555    TGGCACTCGATGGAGCGACAGGCATGCCCGTCCGGACGGATGATGCAA [illegible]
13_c189    ------------------------------------------------ [illegible]
13_c74     TGGCGGTTGATGGCGCGACAGGCATGCCTGTGCGTACGAATGATCCAA [illegible]
14_c324    TGGCACTCGATGGAGCGACAGGCATGCCCGTCCGGACGGATGATGCAA [illegible]
14_c329    TGGCGGTTGATGGCGCGACAGGCATGCCTGTGCGTACGAATGATGCAA [illegible]
15_c188    TGGCACTCGATGGAGCGACAGGCATGCCCGTCCGGACGGATGATGCAA [illegible]
15_c161    TGGCACTTGATGGAGCGACGGGCATGCCTGTACGTACGGGTGATGCAA [illegible]
16_c36     TGGCACTTGATGGAGCGACGGGCATGCCTGTACGTACGGGTGATGCAA [illegible]
17_c39     TGGCGGTTGATGGCGCGACAGGCATGCCTGTGCGTACGAACGATGCAA [illegible]
18_c0      TGGCGGTTGATGGCGCGACAGGCATGCCTGTGCGTACCAATGATGCAA [illegible]
19_c229    TGGCACTCGATGGAGCGACAGGCATGCCCGTCCGGACGGATGATGCAA [illegible]
19_c2      TGGCGGTTGATGGCGCGACAGGCATGCCTGTGCGTACGAATGATGCAA [illegible]
20_c93     TGGCACTCGATGGAGCGACAGGCATGCCCGTCCGGACGAATGATGCAA [illegible]
21_c78     TGGCACTCGATGGAGCGACAGGCATGCCCGTCCGGACGGATGATGCAA [illegible]
22_c436    ------------------------------------------------ [illegible]
22_c3703   ------------------------------------------------ [illegible]
22_c3909   ------------------------------------------------ [illegible]
22_c3246   ------------------------------------------------ [illegible]
22_c3239   ------------------------------------------------ [illegible]
22_c9147   TGGCGGTTGATGGCGCGACAGGCATGCCTGTGCGTACCAATGATGCAT [illegible]
22_c9143   TGGCGGTTGATGGCGCGACAGGCATGCCTGTGCGTACGAATGATCCAA [illegible]
22_c3243   ------------------------------------------------ [illegible]
22_c7804   ------------------------------------------------ [illegible]
22_c8090   ------------------------------------------------ [illegible]
23_c97     TGGCGGTTGATGGCGCGACAGGCATGCCTGTGCGTACCAATGATGCAT [illegible]
24_c78     TGGCACTCGATGGAGCGACAGGCATGCCCGTCCGGACGGATGATGCAA [illegible]
25_c2      TGGCACTCGATGGAGCGACAGGCATGCCCGTCCGGACGGATGATGCAA [illegible]
25_c4      TGGCACTTGATGGAGCGACGGGCATGCCTGTACGTACGGGTGATGCAA [illegible]
26_c640    ------------------------------------------------ [illegible]
26_c3091   ------------------------------------------------ [illegible]
26_c3507   ------------------------------------------------ [illegible]
26_c8864   ------------------------------------------------ [illegible]
26_c9308   ------------------------------------------------ [illegible]
26_c5920   TGGCGGTTGATGGCGCGACAGGCATGCCTGTGCGTACGAATGATGCAA [illegible]
26_c5576   ------------------------------------------------ [illegible]
26_c8886   TGGCGGTTGATGGCGCGACAGGCATGCCTGTGCGTACGAATGATCCAA [illegible]
26_c2334   ------------------------------------------------ [illegible]
26_c8891   ------------------------------------------------ [illegible]
27_c2847   ------------------------------------------------ [illegible]
27_c2264   ------------------------------------------------ [illegible]
27_c1238   TGGCGGTTGATGGCGCGACAGGCATGCCTGTGCGTACGAATGATCCAA [illegible]
27_c8054   ------------------------------------------------ [illegible]
27_c8053   ------------------------------------------------ [illegible]
27_c2660   ------------------------------------------------ [illegible]
27_c2066   ------------------------------------------------ [illegible]
27_c2887   ------------------------------------------------ [illegible]
27_c9392   ------------------------------------------------ [illegible]
27_c9396   ------------------------------------------------ [illegible]
27_c7764   ------------------------------------------------ [illegible]
28_c53     TGGCACTCGATGGAGCGACAGGCATGCCCGTCCGGACGGATGATGCAA [illegible]
29_c114    TGGCACTCGATGGAGCGACAGGCATGCCCGTCCGGACGGATGATGCAA [illegible]
29_c19     TGGCGGTTGATGGCGCGACAGGCATGCCTGTGCGTACGAATGATGCAA [illegible]
30_c1      TGGCACTCGATGGAGCGACAGGCATGCCCGTCCGGACGGATGATGCAA [illegible]
30_c16     TGGCACTTGATGGAGCGACGGGCATGCCTGTACGTACGGGTGATGCAA [illegible]
31_c6068   TGGCAGTTGATGGAGCGACGGGTATGCCCGTACGCTCGGGTGATGCAA [illegible]
31_c1849   ------------------------------------------------ [illegible]
31_c2683   ------------------------------------------------ [illegible]
31_c7078   ------------------------------------------------ [illegible]
31_c3712   ------------------------------------------------ [illegible]
31_c8783   ------------------------------------------------ [illegible]
31_c4585   ------------------------------------------------ [illegible]
31_c11607  TGGCAGTTGATGGAGCGACGGGTATGCCCGTTCGCTCGGGTGATGCAA [illegible]
31_c11132  ------------------------------------------------ [illegible]
31_c11215  ------------------------------------------------ [illegible]
32_c1495   ------------------------------------------------ [illegible]
32_c2827   ------------------------------------------------ [illegible]
32_c1902   ------------------------------------------------ [illegible]
32_c6288   ------------------------------------------------ [illegible]
32_c4518   ------------------------------------------------ [illegible]
32_c10289  TGGCAGTTGATGGAGCGACGGGTATGCCCGTACGCTCGGGTGATGCAA [illegible]
32_c10286  TGGCAGTTGATGGAGCGACGGGTATGCCCGTTCGCTCGGGTGATGCAA [illegible]
32_c12407  ------------------------------------------------ [illegible]
33_c3225   TGGCAGTTGATGGAGCGACGGGTATGCCCGTACGCTCGGGTGATGCAA [illegible]
33_c1289   ------------------------------------------------ [illegible]
33_c3588   ------------------------------------------------ [illegible]
33_c3633   ------------------------------------------------ [illegible]
33_c3542   ------------------------------------------------ [illegible]
33_c8070   ------------------------------------------------ [illegible]
34_c2333   TGGCAGTTGATGGAGCGACGGGTATGCCCGTTCGCTCGGGTGATGCAA [illegible]
34_c2748   ------------------------------------------------ [illegible]
34_c2702   ------------------------------------------------ [illegible]
34_c2106   ------------------------------------------------ [illegible]
34_c3824   ------------------------------------------------ [illegible]
34_c12455  TGGCAGTTGATGGAGCGACGGGTATGCCCGTACGCTCGGGTGATGCAA [illegible]
35_c3229   TGGCAGTTGATGGAGCGACGGGTATGCCCGTTCGCTCGGGTGATGCAA [illegible]
35_c3040   ------------------------------------------------ [illegible]
35_c2986   ------------------------------------------------ [illegible]
35_c3517   ------------------------------------------------ [illegible]
35_c7295   ------------------------------------------------ [illegible]
35_c3583   ------------------------------------------------ [illegible]
35_c11501  ------------------------------------------------ [illegible]
35_c11581  ------------------------------------------------ [illegible]
36_c87     TGGCACTCGATGGAGCGACAGGCATGCCCGTCCGGACGGATGATGCAA [illegible]
36_c2900   TGGCACTTGATGGAGCGACGGGCATGCCTGTACGTACGGGTGATGCAA [illegible]
36_c10327  ------------------------------------------------ [illegible]
36_c5231   ------------------------------------------------ [illegible]
36_c3613   ------------------------------------------------ [illegible]
36_c6503   ------------------------------------------------ [illegible]
36_c5437   ------------------------------------------------ [illegible]
36_c17033  ------------------------------------------------ [illegible]
36_c7222   ------------------------------------------------ [illegible]
36_c25053  ------------------------------------------------ [illegible]
36_c9137   ------------------------------------------------ [illegible]
36_c25163  ------------------------------------------------ [illegible]
36_c27098  ------------------------------------------------ [illegible]
36_c10144  ------------------------------------------------ [illegible]
36_c29808  TGGCAGTTGATGGAGCGACGGGTATGCCCGTACGCTCGGGTGATGCAA [illegible]
36_c29806  TGGCAGTTGATGGAGCGACGGGTATGCCCGTTCGCTCGGGTGATGCAA [illegible]
36_c16773  ------------------------------------------------ [illegible]
37_c0      TGGCACTCGATGGAGCGACAGGCATGCCCGTCCGGACGGATGATGCAA [illegible]
37_c122    TGGCACTTGATGGAGCGACGGGCATGCCTGTACGTACGGGTGATGCAA [illegible]
38_c2      TGGCACTCGATGGAGCGACAGGCATGCCCGTCCGGACGGATGATGCAA [illegible]
38_c21     TGGCACTTGATGGAGCGACGGGCATGCCTGTACGTACGGGTGATGCAA [illegible]
39_c79     TGGCACTCGATGGAGCGACAGGCATGCCCGTCCGGACGGATGATGCAA [illegible]
40_c22     TGGCACTCGATGGAGCGACAGGCATGCCCGTCCGGACGGATGATGCAA [illegible]
40_c16     TGGCACTTGATGGAGCGACGGGCATGCCTGTACGTACGGGTGATGCAA [illegible]
41_c16     TGGCACTCGATGGAGCGACAGGCATGCCCGTCCGGACGGATGATGCAA [illegible]
41_c18     TGGCACTTGATGGAGCGACGGGCATGCCTGTACGTACGGGTGATGCAA [illegible]
42_c3      TGGCACTCGATGGAGCGACAGGCATGCCCGTCCGGACGGATGATGCAA [illegible]
42_c17     TGGCACTTGATGGAGCGACGGGCATGCCTGTACGTACGGGTGATGCAA [illegible]
43_c11     TGGCACTCGATGGAGCGACAGGCATGCCCGTCCGGACGGATGATGCAA [illegible]
43_c0      TGGCACTTGATGGAGCGACGGGCATGCCTGTACGTACGGGTGATGCAA [illegible]
44_c76     TGGCACTCGATGGAGCGACAGGCATGCCCGTCCGGACGGATGATGCAA [illegible]
45_c2879   ------------------------------------------------ [illegible]
45_c2088   TGGCGGTTGATGGCGCGACAGGCATGCCTGTGCGTACCAATGATGCAT [illegible]
45_c2272   ------------------------------------------------ [illegible]
45_c1584   TGGCGGTTGATGGCGCGACAGGCATGCCTGTGCGTACGAATGATCCAA [illegible]
45_c8010   ------------------------------------------------ [illegible]
45_c8487   ------------------------------------------------ [illegible]
45_c2110   ------------------------------------------------ [illegible]
45_c7764   ------------------------------------------------ [illegible]
46_c568    ------------------------------------------------ [illegible]
46_c2890   ------------------------------------------------ [illegible]
46_c3787   ------------------------------------------------ [illegible]
46_c8833   ------------------------------------------------ [illegible]
46_c9063   ------------------------------------------------ [illegible]
46_c5867   TGGCGGTTGATGGCGCGACAGGCATGCCTGTGCGTACGAATGATGCAA [illegible]
46_c5875   TGGCGGTTGATGGCGCGACAGGCATGCCTGTGCGTACGAATGATCCAA [illegible]
46_c4243   ------------------------------------------------ [illegible]
46_c5253   ------------------------------------------------ [illegible]
46_c4182   ------------------------------------------------ [illegible]
47_c654    ------------------------------------------------ [illegible]
47_c866    ------------------------------------------------ [illegible]
47_c1813   TGGCGGTTGATGGCGCGACAGGCATGCCTGTGCGTACGAATGATGCAA [illegible]
47_c2201   ------------------------------------------------ [illegible]
47_c8816   ------------------------------------------------ [illegible]
47_c4862   ------------------------------------------------ [illegible]
47_c8829   TGGCGGTTGATGGCGCGACAGGCATGCCTGTGCGTACGAATGATCCAA [illegible]
47_c2374   ------------------------------------------------ [illegible]
48_c59     TGGCAGTTGATGGAGCGACGGGTATGCCCGTTCGCTCGGGTGATGCAA [illegible]
49_c38     TGGCAGTTGATGGAGCGACGGGTATGCCCGTTCGCTCGGGTGATGCAA [illegible]
50_c1244   ------------------------------------------------ [illegible]
50_c3508   ------------------------------------------------ [illegible]
50_c3451   ------------------------------------------------ [illegible]
50_c3379   ------------------------------------------------ [illegible]
50_c3455   ------------------------------------------------ [illegible]
50_c4315   ------------------------------------------------ [illegible]
50_c3055   ------------------------------------------------ [illegible]
50_c9016   TGGCGGTTGATGGCGCGACAGGCATGCCTGTGCGTACCAATGATGCAT [illegible]
50_c10606  TGGCGGTTGATGGCGCGACAGGCATGCCTGTGCGTACGAATGATCCAA [illegible]
50_c7788   ------------------------------------------------ [illegible]
50_c7790   ------------------------------------------------ [illegible]
51_c344    TGGCACTCGATGGAGCGACAGGCATGCCCGTCCGGACGGATGATGCAA [illegible]
51_c19     TGGCGGTTGATGGCGCGACAGGCATGCCTGTGCGTACGAATGATGCAA [illegible]
52_c81     TGGCACTCGATGGAGCGACAGGCATGCCCGTCCGGACGGATGATGCAA [illegible]
53_c1      TGGCACTCGATGGAGCGACAGGCATGCCCGTCCGGACGGATGATGCAA [illegible]
53_c16     TGGCACTTGATGGAGCGACGGGCATGCCTGTACGTACGGGTGATGCAA [illegible]
54_c2      TGGCACTCGATGGAGCGACAGGCATGCCCGTCCGGACGGATGATGCAA [illegible]
54_c23     TGGCACTTGATGGAGCGACGGGCATGCCTGTACGTACGGGTGATGCAA [illegible]
A_c86      TGGCACTCGATGGAGCGACAGGCATGCCCGTCCGGACGGATGATGCAA [illegible]
A_c234     TGGCACTTGATGGAGCGACGGGCATGCCTGTACGTACGGGTGATGCAA [illegible]
A_c179     ------------------------------------------------ [illegible]
B_c29      TGGCACTCGATGGAGCGACAGGCATGCCCGTCCGGACGGATGATGCAA [illegible]
B_c167     TGGCACTTGATGGAGCGACGGGCATGCCTGTACGTACGGGTGATGCAA [illegible]
B_c233     ------------------------------------------------ [illegible]
C_c82      TGGCACTTGATGGAGCGACGGGCATGCCCGTACGTACGGATGATGCAA [illegible]
D_c114     TGGCACTCGATGGAGCGACAGGCATGCCCGTCCGGACGAATGATGCAA [illegible]
D_c220     ------------------------------------------------ [illegible]
E_c281     TGGCAGTTGATGGAGCGACGGGTATGCCCGTTCGCTCGGGTGATGCAA [illegible]
F_c333     TGGCAGTTGATGGAGCGACGGGTATGCCCGTTCGCTCGGGTGATGCAA [illegible]
F_c277     TGGCGGTTGATGGCGCGACAGGCATGCCTGTGCGTACGAATGATCCAA [illegible]
G_c217     TGGCAGTTGATGGAGCGACGGGTATGCCCGTTCGCTCGGGTGATGCAA [illegible]
G_c346     ------------------------------------------------ [illegible]
G_c3223    ------------------------------------------------ [illegible]
G_c2752    ------------------------------------------------ [illegible]
H_c257     ------------------------------------------------ [illegible]
H_c316     ------------------------------------------------ [illegible]
H_c803     TGGCGGTTGATGGCGCGACAGGCATGCCTGTGCGTACGAATGATCCAA [illegible]
H_c809     TGGCGGTTGATGGCGCGACAGGCATGCCTGTGCGTACCAATGATGCAT [illegible]
I_c2398    TGGCGGTTGATGGCGCGACAGGCATGCCTGTGCGTACGAATGATCCAA [illegible]
I_c6       ------------------------------------------------ [illegible]
I_c211     ------------------------------------------------ [illegible]
I_c2862    ------------------------------------------------ [illegible]
I_c2904    ------------------------------------------------ [illegible]
I_c4553    ------------------------------------------------ [illegible]
I_c3789    TGGCGGTTGATGGCGCGACAGGCATGCCTGTGCGTACGAATGATGCAA [illegible]
J_c192     ------------------------------------------------ [illegible]
J_c435     ------------------------------------------------ [illegible]
J_c3365    TGGCGGTTGATGGCGCGACAGGCAGGCCTGTGCGTACGAATGATGCAA [illegible]
J_c3372    ------------------------------------------------ [illegible]
J_c3012    ------------------------------------------------ [illegible]
J_c531     ------------------------------------------------ [illegible]
J_c4022    TGGCGGTTGATGGCGCGACAGGCATGCCTGTGCGTACGAATGATGCAA [illegible]
K_c2135    ------------------------------------------------ [illegible]
K_c1206    TGGCGGTTGATGGCGCGACAGGCATGCCTGTGCGTACGAATGATCCAA [illegible]
K_c93      ------------------------------------------------ [illegible]
K_c1438    TGGCGGTTGATGGCGCGACAGGCATGCCTGTGCGTACCAATGATGCAT [illegible]
K_c3555    ------------------------------------------------ [illegible]
K_c461     ------------------------------------------------ [illegible]
L_c1200    TGGCGGTTGATGGCGCGACAGGCATGCCTGTGCGTACCAATGATGCAT [illegible]
L_c1178    TGGCGGTTGATGGCGCGACAGGCATGCCTGTGCGTACGAATGATCCAA [illegible]
L_c383     ------------------------------------------------ [illegible]
L_c3401    ------------------------------------------------ [illegible]
L_c444     ------------------------------------------------ [illegible]
```

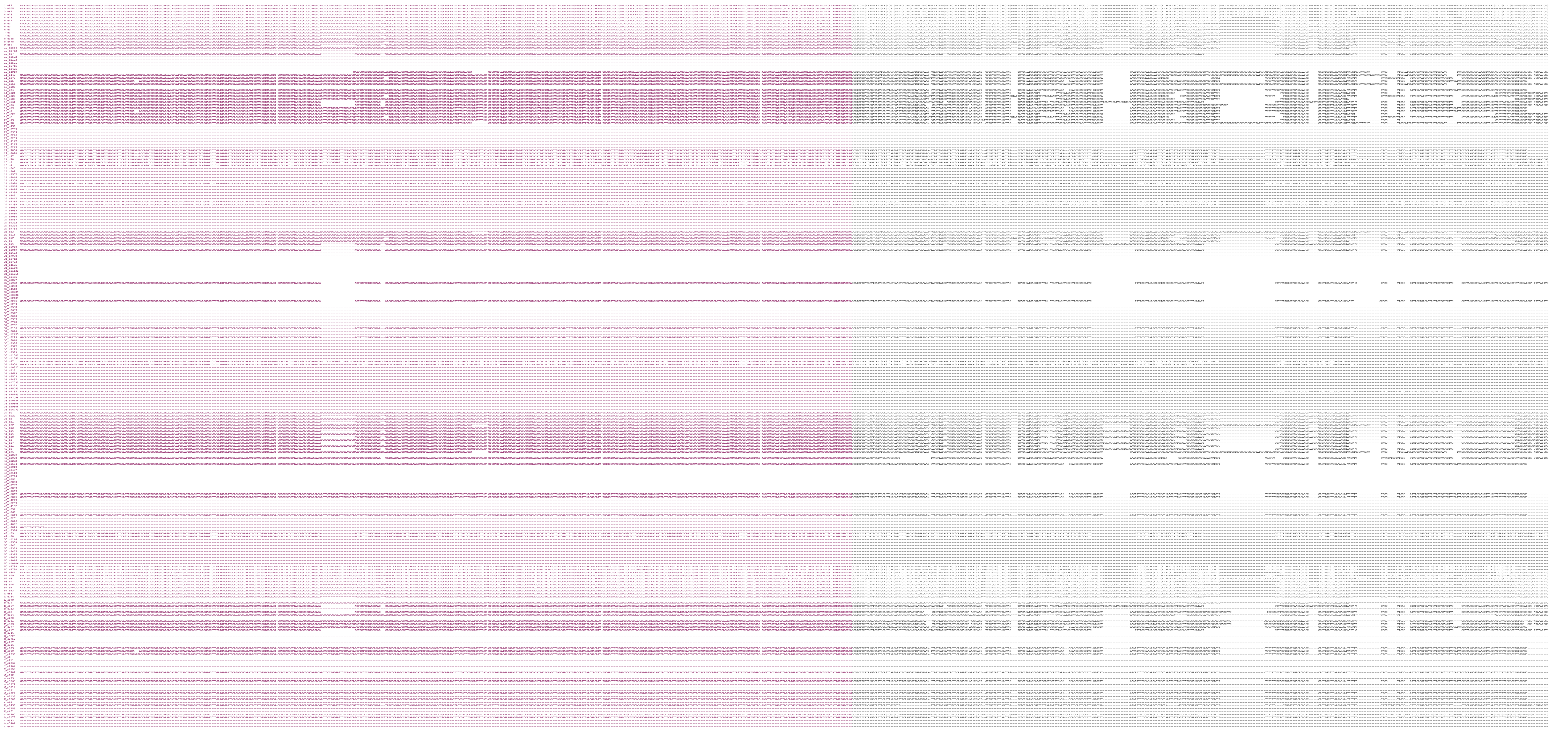

```
1_c85 [illegible]
2_c105 [illegible]
3_c64 [illegible]
4_c26 [illegible]
4_c25 [illegible]
5_c14 [illegible]
5_c0 [illegible]
6_c0 [illegible]
6_c1 [illegible]
7_c1 [illegible]
7_c0 [illegible]
8_c145 [illegible]
8_c478 [illegible]
9_c69 [illegible]
10_c2514 [illegible]
10_c2762 [illegible]
10_c17 [illegible]
10_c1502 [illegible]
10_c2103 [illegible]
10_c3521 [illegible]
10_c6715 [illegible]
10_c3485 [illegible]
10_c4919 [illegible]
11_c443 [illegible]
11_c378 [illegible]
12_c25 [illegible]
13_c555 [illegible]
13_c189 [illegible]
13_c74 [illegible]
14_c324 [illegible]
14_c329 [illegible]
15_c188 [illegible]
15_c161 [illegible]
16_c36 [illegible]
17_c39 [illegible]
18_c0 [illegible]
19_c229 [illegible]
19_c2 [illegible]
20_c93 [illegible]
21_c78 [illegible]
22_c436 [illegible]
22_c3703 [illegible]
22_c3909 [illegible]
22_c3246 [illegible]
22_c3239 [illegible]
22_c9147 [illegible]
22_c9143 [illegible]
22_c3243 [illegible]
22_c7804 [illegible]
22_c8090 [illegible]
23_c97 [illegible]
24_c78 [illegible]
25_c2 [illegible]
25_c4 [illegible]
26_c640 [illegible]
26_c3091 [illegible]
26_c3507 [illegible]
26_c8864 [illegible]
26_c9308 [illegible]
26_c5920 [illegible]
26_c5576 [illegible]
26_c8886 [illegible]
26_c2334 [illegible]
26_c8891 [illegible]
27_c2847 [illegible]
27_c2264 [illegible]
27_c1238 [illegible]
27_c8054 [illegible]
27_c8053 [illegible]
27_c2660 [illegible]
27_c2066 [illegible]
27_c2887 [illegible]
27_c9392 [illegible]
27_c9396 [illegible]
27_c7764 [illegible]
28_c53 [illegible]
29_c114 [illegible]
29_c19 [illegible]
30_c1 [illegible]
30_c16 [illegible]
31_c6068 [illegible]
31_c1849 [illegible]
31_c2683 [illegible]
31_c7078 [illegible]
31_c3712 [illegible]
31_c8783 [illegible]
31_c4585 [illegible]
31_c11607 [illegible]
31_c11132 [illegible]
31_c11215 [illegible]
32_c1495 [illegible]
32_c2827 [illegible]
32_c1902 [illegible]
32_c6288 [illegible]
32_c4518 [illegible]
32_c10289 [illegible]
32_c10286 [illegible]
32_c12407 [illegible]
33_c3225 [illegible]
33_c1289 [illegible]
33_c3588 [illegible]
33_c3633 [illegible]
33_c3542 [illegible]
33_c8070 [illegible]
34_c2333 [illegible]
34_c2748 [illegible]
34_c2702 [illegible]
34_c2106 [illegible]
34_c3824 [illegible]
34_c12455 [illegible]
35_c3229 [illegible]
35_c3040 [illegible]
35_c2986 [illegible]
35_c3517 [illegible]
35_c7295 [illegible]
35_c3583 [illegible]
35_c11501 [illegible]
35_c11581 [illegible]
36_c87 [illegible]
36_c2900 [illegible]
36_c10327 [illegible]
36_c5231 [illegible]
36_c3613 [illegible]
36_c6503 [illegible]
36_c5437 [illegible]
36_c17033 [illegible]
36_c7222 [illegible]
36_c25053 [illegible]
36_c9137 [illegible]
36_c25163 [illegible]
36_c27098 [illegible]
36_c10144 [illegible]
36_c29808 [illegible]
36_c29806 [illegible]
36_c16773 [illegible]
37_c0 [illegible]
37_c122 [illegible]
38_c2 [illegible]
38_c21 [illegible]
39_c79 [illegible]
40_c22 [illegible]
40_c16 [illegible]
41_c16 [illegible]
41_c18 [illegible]
42_c3 [illegible]
42_c17 [illegible]
43_c11 [illegible]
43_c0 [illegible]
44_c76 [illegible]
45_c2879 [illegible]
45_c2088 [illegible]
45_c2272 [illegible]
45_c1584 [illegible]
45_c8010 [illegible]
45_c8487 [illegible]
45_c2110 [illegible]
45_c7764 [illegible]
46_c568 [illegible]
46_c2890 [illegible]
46_c3787 [illegible]
46_c8833 [illegible]
46_c9063 [illegible]
46_c5867 [illegible]
46_c5875 [illegible]
46_c4243 [illegible]
46_c5253 [illegible]
46_c4182 [illegible]
47_c654 [illegible]
47_c866 [illegible]
47_c1813 [illegible]
47_c2201 [illegible]
47_c8816 [illegible]
47_c4862 [illegible]
47_c8829 [illegible]
47_c2374 [illegible]
48_c59 [illegible]
49_c38 [illegible]
50_c1244 [illegible]
50_c3508 [illegible]
50_c3451 [illegible]
50_c3379 [illegible]
50_c3455 [illegible]
50_c4315 [illegible]
50_c3055 [illegible]
50_c9016 [illegible]
50_c10606 [illegible]
50_c7788 [illegible]
50_c7790 [illegible]
51_c344 [illegible]
51_c19 [illegible]
52_c81 [illegible]
53_c1 [illegible]
53_c16 [illegible]
54_c2 [illegible]
54_c23 [illegible]
A_c86 [illegible]
A_c234 [illegible]
A_c179 [illegible]
B_c29 [illegible]
B_c167 [illegible]
B_c233 [illegible]
C_c82 [illegible]
D_c114 [illegible]
D_c220 [illegible]
E_c281 [illegible]
F_c333 [illegible]
F_c277 [illegible]
G_c217 [illegible]
G_c346 [illegible]
G_c3223 [illegible]
G_c2752 [illegible]
H_c257 [illegible]
H_c316 [illegible]
H_c803 [illegible]
H_c809 [illegible]
I_c2398 [illegible]
I_c6 [illegible]
I_c211 [illegible]
I_c2862 [illegible]
I_c2904 [illegible]
I_c4553 [illegible]
I_c3789 [illegible]
J_c192 [illegible]
J_c435 [illegible]
J_c3365 [illegible]
J_c3372 [illegible]
J_c3012 [illegible]
J_c531 [illegible]
J_c4022 [illegible]
K_c2135 [illegible]
K_c1206 [illegible]
K_c93 [illegible]
K_c1438 [illegible]
K_c3555 [illegible]
K_c461 [illegible]
L_c1200 [illegible]
L_c1178 [illegible]
L_c383 [illegible]
L_c3401 [illegible]
L_c444 [illegible]
```

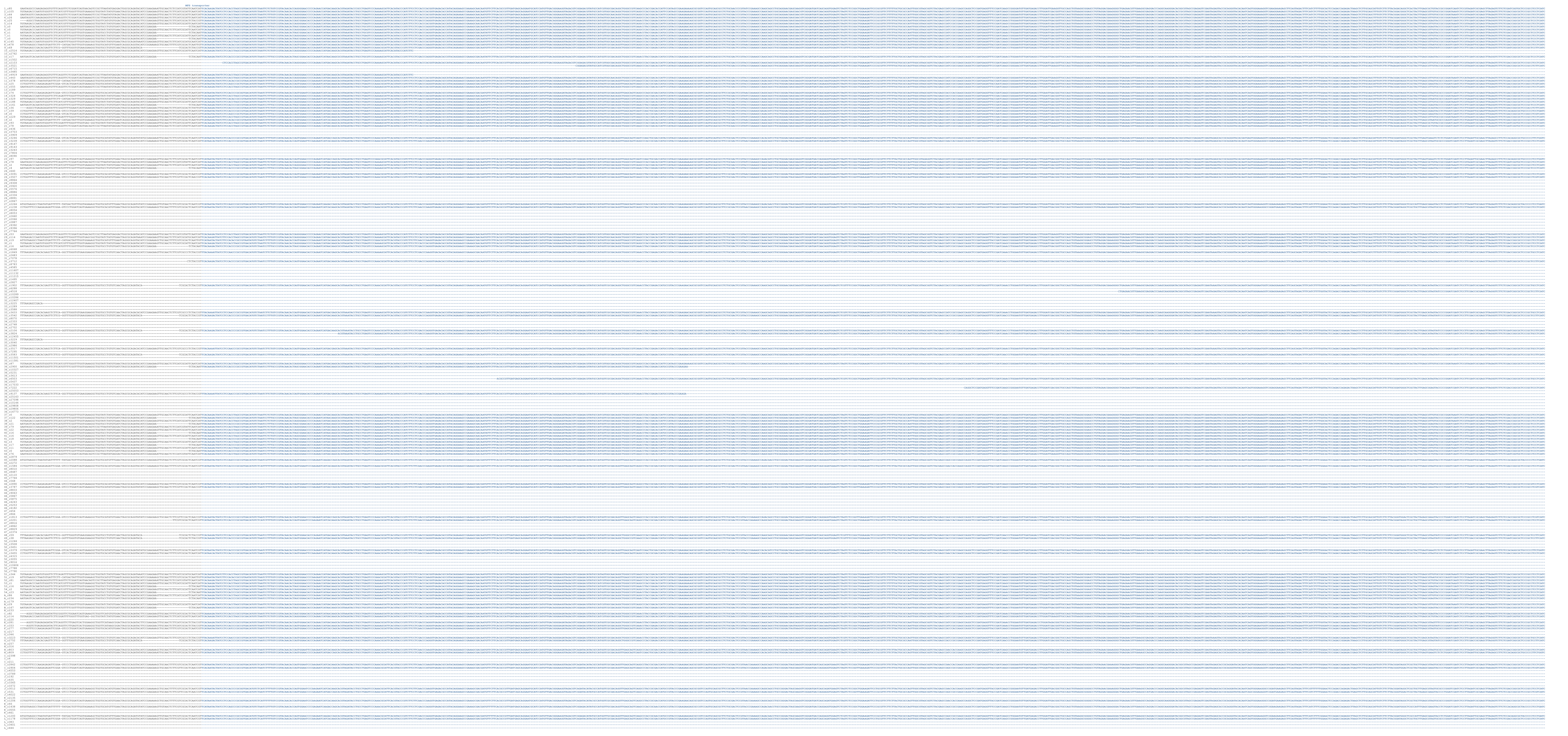
MFS transporter

```
1_c85      ATTAGGCGAGTGCGGCGGAGACGGTGG[illegible]
2_c105     ATTCGGCGAGTGTGGCGGAGACGGTGG[illegible]
3_c64      ATTCGGCGAGTGTGGCGGAGACGGTGG[illegible]
4_c26      ATTCGGCGAGTGTGGCGGAGACGGTGG[illegible]
4_c25      ATTCGACGAGTGTGACGGAGACGGCGG[illegible]
5_c14      ATTCGGCGAGTGTGGCGGAGACGGTGG[illegible]
5_c0       ATTCGACGAGTGTGACGGAGACGGCGG[illegible]
6_c0       ATTCGGCGAGTGTGGCGGAGACGGTGG[illegible]
6_c1       ATTCGACGAGTGTGACGGAGACGGCGG[illegible]
7_c1       ATTCGGCGAGTGTGGCGGAGACGGTGG[illegible]
7_c0       ATTCGACGAGTGTGACGGAGACGGCGG[illegible]
8_c145     ATTCGGCGAGTGTGGCGGAGACGATGG[illegible]
8_c478     ATATGGCGAGTGGGACGTAGACTGTGC[illegible]
9_c69      ATTCGACGAGTGCGACGGAGACGGTGG[illegible]
10_c2514   ---------------------------[illegible]
10_c2762   ---------------------------[illegible]
10_c17     ATTCGACGAGTGTGACGGAGACGGCGG[illegible]
10_c1502   ---------------------------[illegible]
10_c2103   ATTCGGCGAGTGTGGCGGAGACGGTGG[illegible]
10_c3521   ATTCGGCGAGTGCGGCGGAGACGGTGG[illegible]
10_c6715   ---------------------------[illegible]
10_c3485   ---------------------------[illegible]
10_c4919   ---------------------------[illegible]
11_c443    ATTCGGCGAGTGTGGCGGAGACGGTGG[illegible]
11_c378    ATATGGCGAGTGGGACGTAGACTGTGT[illegible]
12_c25     ATTCGGCGAATGTGACGGCGACGGTAC[illegible]
13_c555    ATTCGGCGAGTGTGGCGGAGACGATGG[illegible]
13_c189    ---------------------------[illegible]
13_c74     ACATGGCGAGTGGGACGTAGACTGTGC[illegible]
14_c324    ATTCGGCGAGTGTGGCGGAGACGATGG[illegible]
14_c329    ATATGGCGAGTGGGACGTAGACTGTGC[illegible]
15_c188    ATTCGGCGAGTGTGGCGGAGACGGTGG[illegible]
15_c161    ATTCGACGAGTGTGACGGAGACGGCGG[illegible]
16_c36     ATTCGCCGAGTGTGACGGAGACGGTGG[illegible]
17_c39     ACTCGGCGAGTGTGACGGAGAAACTGG[illegible]
18_c0      ATTCGGCGAATGTGACGGCGACGGTAC[illegible]
19_c229    ATTCGGCGAGTGTGGCGGAGACGATGG[illegible]
19_c2      ATATGGCGAGTGGGACGTAGACTGTGC[illegible]
20_c93     ATTAGGCGAGTGCGGCGGAGACGGTGG[illegible]
21_c78     ATTAGGCGAGTGCGGCGGAGACGGTGG[illegible]
22_c436    ---------------------------[illegible]
22_c3703   ---------------------------[illegible]
22_c3909   ---------------------------[illegible]
22_c3246   ACTCGGCGAGTGTGACGGAGAAACTGG[illegible]
22_c3239   ACATGGCGAGTGGGACGTAGACTGTGC[illegible]
22_c9147   ---------------------------[illegible]
22_c9143   ---------------------------[illegible]
22_c3243   ---------------------------[illegible]
22_c7804   ---------------------------[illegible]
22_c8090   ---------------------------[illegible]
23_c97     ACTCGGCGAGTGTGACGGAGAAACTGG[illegible]
24_c78     ATTAGGCGAGTGCGGCGGAGACGGTGG[illegible]
25_c2      ATTCGGCGAGTGTGGCGGAGACGGTGG[illegible]
25_c4      ATTCGACGAGTGTGACGGAGACGGCGG[illegible]
26_c640    ---------------------------[illegible]
26_c3091   ACATGGCGAGTGGGACGTAGACTGTGC[illegible]
26_c3507   AGTTGGCGAGTGTGACGTAGACTGCGG[illegible]
26_c8864   ---------------------------[illegible]
26_c9308   ---------------------------[illegible]
26_c5920   ---------------------------[illegible]
26_c5576   ---------------------------[illegible]
26_c8886   ---------------------------[illegible]
26_c2334   ---------------------------[illegible]
26_c8891   ---------------------------[illegible]
27_c2847   ---------------------------[illegible]
27_c2264   ACTCGGCGAGTGTGACGGAGAAACTAG[illegible]
27_c1238   ACATGGCGAGTGGGACGTAGACTGTGC[illegible]
27_c8054   ---------------------------[illegible]
27_c8053   ---------------------------[illegible]
27_c2660   ---------------------------[illegible]
27_c2066   ---------------------------[illegible]
27_c2887   ---------------------------[illegible]
27_c9392   ---------------------------[illegible]
27_c9396   ---------------------------[illegible]
27_c7764   ---------------------------[illegible]
28_c53     ATTCGGCGAGTGTGGCGGAGACGGTGG[illegible]
29_c114    ATTCGGCGAGTGTGGCGGAGACGATGG[illegible]
29_c19     ATATGGCGAGTGGGACGTAGACTGTGC[illegible]
30_c1      ATTCGGCGAGTGTGGCGGAGACGGTGG[illegible]
30_c16     ATTCGACGAGTGTGACGGAGACGGCGG[illegible]
31_c6068   ---------------------------[illegible]
31_c1849   ATTCAACGAGTGTGACGGAGACGGTGG[illegible]
31_c2683   ---------------------------[illegible]
31_c7078   ---------------------------[illegible]
31_c3712   ATTCGACGAGTGCGACGGAGACGGTGG[illegible]
31_c8783   ---------------------------[illegible]
31_c4585   ---------------------------[illegible]
31_c11607  ---------------------------[illegible]
31_c11132  ---------------------------[illegible]
31_c11215  ---------------------------[illegible]
32_c1495   ---------------------------[illegible]
32_c2827   ---------------------------[illegible]
32_c1902   ATTCAACGAGTGTGACGGAGACGGTGG[illegible]
32_c6288   ---------------------------[illegible]
32_c4518   ATTCGACGAGTGCGACGGAGACGGTGG[illegible]
32_c10289  ---------------------------[illegible]
32_c10286  ---------------------------[illegible]
32_c12407  ---------------------------[illegible]
33_c3225   ---------------------------[illegible]
33_c1289   ---------------------------[illegible]
33_c3588   ---------------------------[illegible]
33_c3633   ATTCAACGAGTGTGACGGAGACGGTGG[illegible]
33_c3542   ATTCGACGAGTGCGACGGAGACGGTGG[illegible]
33_c8070   ---------------------------[illegible]
34_c2333   ---------------------------[illegible]
34_c2748   ---------------------------[illegible]
34_c2702   ---------------------------[illegible]
34_c2106   ATTCGACGAGTGCGACGGAGACGGTGG[illegible]
34_c3824   ATTCAACGAGTGTGACGGAGACGGTGG[illegible]
34_c12455  ---------------------------[illegible]
35_c3229   ---------------------------[illegible]
35_c3040   ---------------------------[illegible]
35_c2986   ---------------------------[illegible]
35_c3517   ATTCAACGAGTGTGACGGAGACGGTGG[illegible]
35_c7295   ---------------------------[illegible]
35_c3583   ATTCGACGAGTGCGACGGAGACGGTGG[illegible]
35_c11501  ---------------------------[illegible]
35_c11581  ---------------------------[illegible]
36_c87     ATTCGGCGAGTGTGGCGGAGACGGTGG[illegible]
36_c2900   ---------------------------[illegible]
36_c10327  ---------------------------[illegible]
36_c5231   ---------------------------[illegible]
36_c3613   ---------------------------[illegible]
36_c6503   ATTCAACGAGTGTGACGGAGACGGTGG[illegible]
36_c5437   ---------------------------[illegible]
36_c17033  ---------------------------[illegible]
36_c7222   ATTCGACGAGTGTGACGGAGACGGCGG[illegible]
36_c25053  ---------------------------[illegible]
36_c9137   ---------------------------[illegible]
36_c25163  ---------------------------[illegible]
36_c27098  ---------------------------[illegible]
36_c10144  ---------------------------[illegible]
36_c29808  ---------------------------[illegible]
36_c29806  ---------------------------[illegible]
36_c16773  ---------------------------[illegible]
37_c0      ATTCGGCGAGTGTGGCGGAGACGGTGG[illegible]
37_c122    ATTCGACGAGTGTGACGGAGACGGCGG[illegible]
38_c2      ATTCGGCGAGTGTGGCGGAGACGGTGG[illegible]
38_c21     ATTCGACGAGTGTGACGGAGACGGCGG[illegible]
39_c79     ATTAGGCGAGTGCGGCGGAGACGGTGG[illegible]
40_c22     ATTCGGCGAGTGTGGCGGAGACGGTGG[illegible]
40_c16     ATTCGACGAGTGTGACGGAGACGGCGG[illegible]
41_c16     ATTCGGCGAGTGTGGCGGAGACGGTGG[illegible]
41_c18     ATTCGACGAGTGTGACGGAGACGGCGG[illegible]
42_c3      ATTCGGCGAGTGTGGCGGAGACGGTGG[illegible]
42_c17     ATTCGACGAGTGTGACGGAGACGGCGG[illegible]
43_c11     ATTCGGCGAGTGTGGCGGAGACGGTGG[illegible]
43_c0      ATTCGACGAGTGTGACGGAGACGGCGG[illegible]
44_c76     ATTAGGCGAGTGCGGCGGAGACGGTGG[illegible]
45_c2879   ---------------------------[illegible]
45_c2088   ACTCGGCGAGTGTGACGGAGAAACTAG[illegible]
45_c2272   ---------------------------[illegible]
45_c1584   ACATGGCGAGTGGGACGTAGACTGTGC[illegible]
45_c8010   ---------------------------[illegible]
45_c8487   ---------------------------[illegible]
45_c2110   ---------------------------[illegible]
45_c7764   ---------------------------[illegible]
46_c568    ---------------------------[illegible]
46_c2890   ACATGGCGAGTGGGACGTAGACTGTGC[illegible]
46_c3787   AGTTGGCGAGTGTGACGTAGACTGCGG[illegible]
46_c8833   ---------------------------[illegible]
46_c9063   ---------------------------[illegible]
46_c5867   ---------------------------[illegible]
46_c5875   ---------------------------[illegible]
46_c4243   ---------------------------[illegible]
46_c5253   ---------------------------[illegible]
46_c4182   ---------------------------[illegible]
47_c654    ---------------------------[illegible]
47_c866    ---------------------------[illegible]
47_c1813   AGTTGGCGAGTGTGACGTAGACTGCGG[illegible]
47_c2201   ACATGGCGAGTGGGACGTAGACTGTGC[illegible]
47_c8816   ---------------------------[illegible]
47_c4862   ---------------------------[illegible]
47_c8829   ---------------------------[illegible]
47_c2374   ---------------------------[illegible]
48_c59     ATTCGACGAGTGCGACGGAGACGGTGG[illegible]
49_c38     ATTCGACGAGTGCGACGGAGACGGTGG[illegible]
50_c1244   ---------------------------[illegible]
50_c3508   ---------------------------[illegible]
50_c3451   ---------------------------[illegible]
50_c3379   ACTCGGCGAGTGTGACGGAGAAACTGG[illegible]
50_c3455   ACATGGCGAGTGGGACGTAGACTGTGC[illegible]
50_c4315   ---------------------------[illegible]
50_c3055   ---------------------------[illegible]
50_c9016   ---------------------------[illegible]
50_c10606  ---------------------------[illegible]
50_c7788   ---------------------------[illegible]
50_c7790   ---------------------------[illegible]
51_c344    ATTCGGCGAGTGTGGCGGAGACGATGG[illegible]
51_c19     ATATGGCGAGTGGGACGTAGACTGTGC[illegible]
52_c81     ATTAGGCGAGTGCGGCGGAGACGGTGG[illegible]
53_c1      ATTCGGCGAGTGTGGCGGAGACGGTGG[illegible]
53_c16     ATTCGACGAGTGTGACGGAGACGGCGG[illegible]
54_c2      ATTCGGCGAGTGTGGCGGAGACGGTGG[illegible]
54_c23     ATTCGACGAGTGTGACGGAGACGGCGG[illegible]
A_c86      ATTCGGCGAGTGTGGCGGAGACGGTGG[illegible]
A_c234     ATTCGACGAGTGTGACGGAGACGGCGG[illegible]
A_c179     ---------------------------[illegible]
B_c29      ATTCGGCGAGTGTGGCGGAGACGGTGG[illegible]
B_c167     ATTCGACGAGTGTGACGGAGACGGCGG[illegible]
B_c233     ---------------------------[illegible]
C_c82      ATTCGACGAGTGTGACGGAGACGGCGG[illegible]
D_c114     ATTAGGCGAGTGCGGCGGAGACGGTGG[illegible]
D_c220     ---------------------------[illegible]
E_c281     ATTCGACGAGTGCGACGGAGACGGTGG[illegible]
F_c333     ATTCGACGAATGTGATGGAGACGGTGG[illegible]
F_c277     ACATGGCGAGTCAGACGTAGACTGTGC[illegible]
G_c217     ---------------------------[illegible]
G_c346     ---------------------------[illegible]
G_c3223    ATTCAACGAGTGTGACGGAGACGGTGG[illegible]
G_c2752    ATTCGACGAGTGCGACGGAGACGGTGG[illegible]
H_c257     ---------------------------[illegible]
H_c316     ---------------------------[illegible]
H_c803     ACATGGCGAGTGGGACGTAGACTGTGC[illegible]
H_c809     ACTCGGCGAGTGTGACGGAGAAACTGG[illegible]
I_c2398    ---------------------------[illegible]
I_c6       ---------------------------[illegible]
I_c211     ---------------------------[illegible]
I_c2862    ACATGGCGAGTGGGACGTAGACTGTGC[illegible]
I_c2904    AGTTGGCGAGTGTGACGTAGACTGCGG[illegible]
I_c4553    ---------------------------[illegible]
I_c3789    ---------------------------[illegible]
J_c192     ---------------------------[illegible]
J_c435     ---------------------------[illegible]
J_c3365    ---------------------------[illegible]
J_c3372    ---------------------------[illegible]
J_c3012    ACATGGCGAGTGGGACGTAGACTGTGC[illegible]
J_c531     AGTTGGCGAGTGTGACGTAGACTGCGG[illegible]
J_c4022    ---------------------------[illegible]
K_c2135    ---------------------------[illegible]
K_c1206    ACATGGCGAGTGGGACGTAGACTGTGC[illegible]
K_c93      ---------------------------[illegible]
K_c1438    ACTCGGCGAGTGTGACGGAGAAACTAG[illegible]
K_c3555    ---------------------------[illegible]
K_c461     ---------------------------[illegible]
L_c1200    ACTCGGCGAGTGTGACGGAGAAACTAG[illegible]
L_c1178    ACATGGCGAGTGGGACGTAGACTGTGC[illegible]
L_c383     ---------------------------[illegible]
L_c3401    ---------------------------[illegible]
L_c444     ---------------------------[illegible]
```
