# Supplementary material for: Clonality, inbreeding, and hybridization in two extremotolerant black yeasts
Source: Gigascience. 2022 Oct 6;11:giac095. doi: 10.1093/gigascience/giac095 (PMC9535773; doi:10.1093/gigascience/giac095)
Supplement: giac095_Supplemental_Files [file giac095_supplemental_files.zip › FigS5.rev1.pdf]

MAT1-1

Phylogeny of MAT1-1 regions in genomes of *A. melanogenum* (left) and the presence/absence of putative MAT1-1 genes in the sequenced strains (right; hybridisation tree as in the main Fig. 4). MAT genes can be missing due to (i) absence in the genome *or* (ii) incomplete assembly of the MAT locus during genome assembly. Alignment of the entire putative mating loci is below.

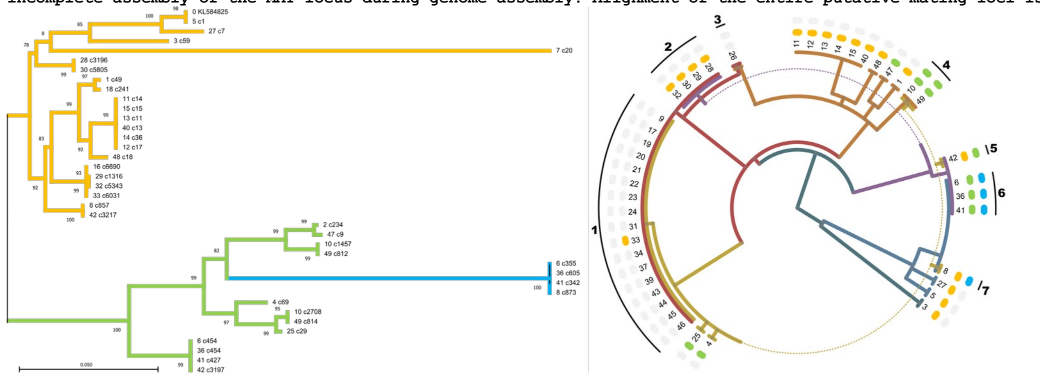

Depth of seuencing of MAT regions. The location of MAT genes are marked with yellow (MAT1-1) and red (MAT1-2). Note that gaps in coverage can be caused by the absence of the region or the high divergence of the region from the reference MAT region (such as in the case of green and blue MAT1-1 clusters shown in the phylogeny on the left).

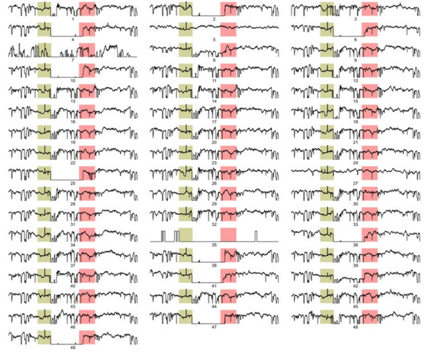

MAT1-2

Phylogeny of MAT1-2 regions in genomes of *A. melanogenum* (left) and the presence/absence of putative MAT1-2 genes in the sequenced strains (right; hybridisation tree as in the main Fig. 4). MAT genes can be missing due to (i) absence in the genome <u>or</u> (ii) incomplete assembly of the MAT locus during genome assembly. Alignment of the entire putative mating loci is below.

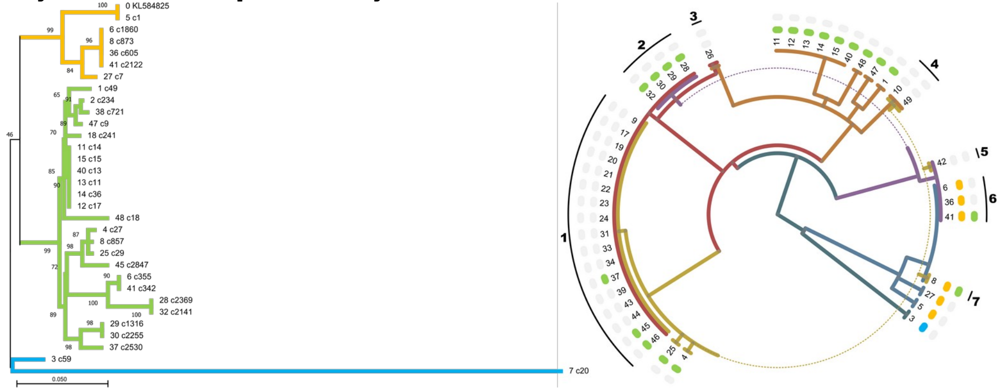

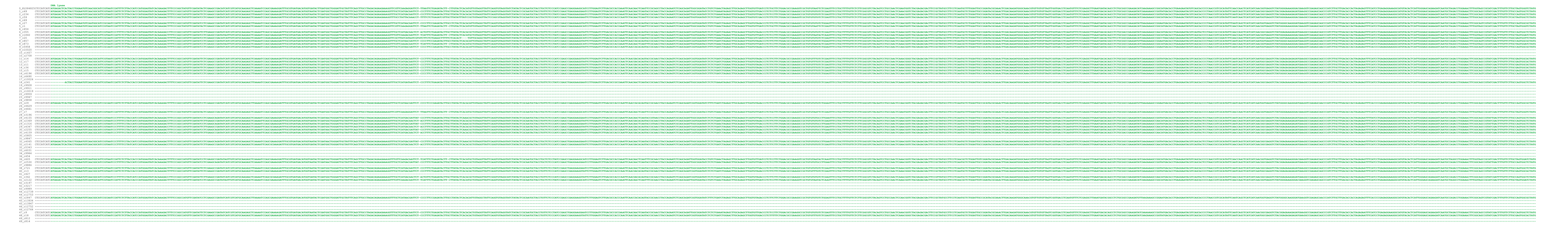

DNA lyase
0_KL584825
1_c49
2_c234
3_c59
4_c69
4_c27
5_c1
6_c454
6_c355
6_c1860
7_c20
8_c857
8_c873
9_c5958
9_c10020
10_c1457
10_c2708
11_c14
12_c17
13_c11
14_c36
15_c15
16_c6196
16_c6690
17_c10024
18_c241
19_c9928
20_c9911
21_c10019
22_c9959
23_c9947
24_c9858
25_c29
26_c4629
26_c4661
27_c7
28_c3196
28_c4155
28_c2369
29_c1316
29_c4347
30_c2255
30_c4159
30_c5805
31_c9847
32_c4065
32_c2141
32_c5343
33_c6031
34_c9964
36_c454
36_c605
37_c2530
37_c9886
38_c721
40_c13
41_c427
41_c342
41_c2122
42_c3197
42_c3217
43_c9989
44_c12739
44_c12753
45_c2847
45_c13934
45_c13947
46_c12752
46_c12764
47_c9
48_c18
49_c812
49_c814

```
0_KL584825CGCGACTGGTTTGTTCATGCCGATATTCAGGAAGGTCTAATGGT[illegible]
1_c49     CGCGATTGGTTTGTTCATGCCGATATCCAAGAAGGTCTCATGGT[illegible]
2_c234    CGCGATTGGTTTGTTCATGCCGATATCCAAGAAGGTCTCATGGT[illegible]
3_c59     CGCGACTGGTTTGTTCATGCCGATATTCAGGAAGGTCTCATGGT[illegible]
4_c69     --------------------------------------------[illegible]
4_c27     CGCGATTGGTTTGTTCATGCCGATATTCAGGAAGGTCTCATGGT[illegible]
5_c1      CGCGACTGGTTTGTTCATGCCGATATTCAGGAAGGTCTAATGGT[illegible]
6_c454    --------------------------------------------[illegible]
6_c355    CGCGATTGGTTTGTTCATGCCGATATTCAAGAAGGTCTCATGGT[illegible]
6_c1860   CGCGACTGGTTTGTTCATGCCGATATTCAGGAAGGTCTAATGGT[illegible]
7_c20     CGCGATTGGTTCGTCCATGCCGATATTCAAGAAGGATTAATGGT[illegible]
8_c857    CGCGATTGGTTTGTTCATGCCGATATTCAAGAAGGTCTCATGGT[illegible]
8_c873    CGCGACTGGTTTGTTCATGCCGATATTCAGGAAGGTCTAATGGT[illegible]
9_c5958   CGCGATTGGTTTGTTCATGCCGATATTCAAGAAGGTCTCATGGT[illegible]
9_c10020  --------------------------------------------[illegible]
10_c1457  --------------------------------------------[illegible]
10_c2708  --------------------------------------------[illegible]
11_c14    CGCGATTGGTTTGTTCATGCCGATATTCAAGAAGGTCTCATGGT[illegible]
12_c17    CGCGATTGGTTTGTTCATGCCGATATTCAAGAAGGTCTCATGGT[illegible]
13_c11    CGCGATTGGTTTGTTCATGCCGATATTCAAGAAGGTCTCATGGT[illegible]
14_c36    CGCGATTGGTTTGTTCATGCCGATATTCAAGAAGGTCTCATGGT[illegible]
15_c15    CGCGATTGGTTTGTTCATGCCGATATTCAAGAAGGTCTCATGGT[illegible]
16_c6196  CGCGATTGGTTTGTTCATGCCGATATTCAAGAAGGTCTCATGGT[illegible]
16_c6690  --------------------------------------------[illegible]
17_c10024 --------------------------------------------[illegible]
18_c241   CACGATTGGTTTGTTCATGCCGATATTCAAGAAGGTCTCATGGT[illegible]
19_c9928  --------------------------------------------[illegible]
20_c9911  --------------------------------------------[illegible]
21_c10019 --------------------------------------------[illegible]
22_c9959  --------------------------------------------[illegible]
23_c9947  --------------------------------------------[illegible]
24_c9858  --------------------------------------------[illegible]
25_c29    CGCGATTGGTTTGTTCATGCCGATATTCAAGAAGGTCTCATGGT[illegible]
26_c4629  --------------------------------------------[illegible]
26_c4661  --------------------------------------------[illegible]
27_c7     CGCGACTGGTTTGTTCATGCCGATATTCAGGAAGGTCTAATGGT[illegible]
28_c3196  --------------------------------------------[illegible]
28_c4155  CGCGATTGGTTTGTTCATGCCGATATTCAAGAAGGTCTCATGGT[illegible]
28_c2369  CGCGATTGGTTTGTTCATGCCGATATTCAAGAAGGTCTCATGGT[illegible]
29_c1316  CGCGATTGGTTTGTTCATGCCGATATTCAAGAAGGTCTCATGGT[illegible]
29_c4347  CGCGATTGGTTTGTTCATGCCGATATTCAAGAAGGTCTCATGGT[illegible]
30_c2255  CGCGATTGGTTTGTTCATGCCGATATTCAAGAAGGTCTCATGGT[illegible]
30_c4159  CGCGATTGGTTTGTTCATGCCGATATTCAAGAAGGTCTCATGGT[illegible]
30_c5805  --------------------------------------------[illegible]
31_c9847  --------------------------------------------[illegible]
32_c4065  CGCGATTGGTTTGTTCATGCCGATATTCAAGAAGGTCTCATGGT[illegible]
32_c2141  CGCGATTGGTTTGTTCATGCCGATATTCAAGAAGGTCTCATGGT[illegible]
32_c5343  --------------------------------------------[illegible]
33_c6031  --------------------------------------------[illegible]
34_c9964  --------------------------------------------[illegible]
36_c454   --------------------------------------------[illegible]
36_c605   CGCGACTGGTTTGTTCATGCCGATATTCAGGAAGGTCTAATGGT[illegible]
37_c2530  CGCGATTGGTTTGTTCATGCCGATATTCAAGAAGGTCTCATGGT[illegible]
37_c9886  --------------------------------------------[illegible]
38_c721   CACGATTGGTTTGTTCATGCCGATATTCAAGAAGGTCTCATGGT[illegible]
40_c13    CGCGATTGGTTTGTTCATGCCGATATTCAAGAAGGTCTCATGGT[illegible]
41_c427   --------------------------------------------[illegible]
41_c342   CGCGATTGGTTTGTTCATGCCGATATTCAAGAAGGTCTCATGGT[illegible]
41_c2122  CGCGACTGGTTTGTTCATGCCGATATTCAGGAAGGTCTAATGGT[illegible]
42_c3197  --------------------------------------------[illegible]
42_c3217  --------------------------------------------[illegible]
43_c9989  --------------------------------------------[illegible]
44_c12739 --------------------------------------------[illegible]
44_c12753 --------------------------------------------[illegible]
45_c2847  CGCGATTGGTTTGTTCATGCCGATATTCAAGAAGGTCTCATGGT[illegible]
45_c13934 --------------------------------------------[illegible]
45_c13947 --------------------------------------------[illegible]
46_c12752 --------------------------------------------[illegible]
46_c12764 --------------------------------------------[illegible]
47_c9     CGCGATTGGTTTGTTCATGCCGATATCCAAGAAGGTCTCATGGT[illegible]
48_c18    CGCGATTGGTTTGTTCATGCCGATATTCAAGAAGGTCTCATGGT[illegible]
49_c812   --------------------------------------------[illegible]
49_c814   --------------------------------------------[illegible]
```

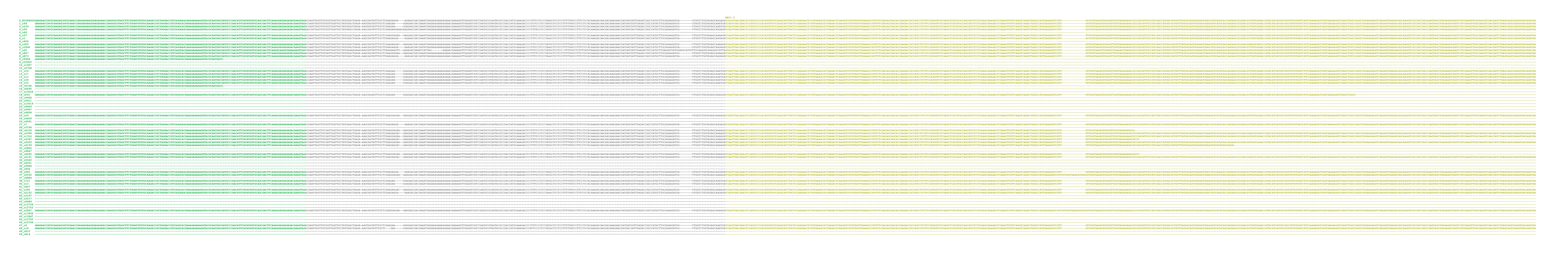
MAT1-2

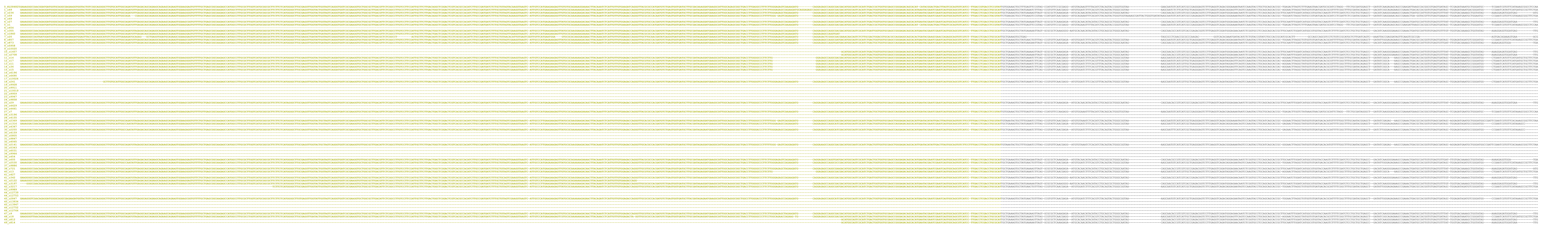

```
0_KL584825 [illegible]
1_c49 [illegible]
2_c234 [illegible]
3_c59 [illegible]
4_c69 [illegible]
4_c27 [illegible]
5_c1 [illegible]
6_c454 [illegible]
6_c355 [illegible]
6_c1860 [illegible]
7_c20 [illegible]
8_c857 [illegible]
8_c873 [illegible]
9_c5958 [illegible]
9_c10020 [illegible]
10_c1457 [illegible]
10_c2708 [illegible]
11_c14 [illegible]
12_c17 [illegible]
13_c11 [illegible]
14_c36 [illegible]
15_c15 [illegible]
16_c6196 [illegible]
16_c6690 [illegible]
17_c10024 [illegible]
18_c241 [illegible]
19_c9928 [illegible]
20_c9911 [illegible]
21_c10019 [illegible]
22_c9959 [illegible]
23_c9947 [illegible]
24_c9858 [illegible]
25_c29 [illegible]
26_c4629 [illegible]
26_c4661 [illegible]
27_c7 [illegible]
28_c3196 [illegible]
28_c4155 [illegible]
28_c2369 [illegible]
29_c1316 [illegible]
29_c4347 [illegible]
30_c2255 [illegible]
30_c4159 [illegible]
30_c5805 [illegible]
31_c9847 [illegible]
32_c4065 [illegible]
32_c2141 [illegible]
32_c5343 [illegible]
33_c6031 [illegible]
34_c9964 [illegible]
36_c454 [illegible]
36_c605 [illegible]
37_c2530 [illegible]
37_c9886 [illegible]
38_c721 [illegible]
40_c13 [illegible]
41_c427 [illegible]
41_c342 [illegible]
41_c2122 [illegible]
42_c3197 [illegible]
42_c3217 [illegible]
43_c9989 [illegible]
44_c12739 [illegible]
44_c12753 [illegible]
45_c2847 [illegible]
45_c13934 [illegible]
45_c13947 [illegible]
46_c12752 [illegible]
46_c12764 [illegible]
47_c9 [illegible]
48_c18 [illegible]
49_c812 [illegible]
49_c814 [illegible]
```

```
0_KL584825 AGCTTTCCAGACTATCGCCGCGTCATTCGCACCTCATACCAGACCCTTCCCCAAGCCGCCGCTGGGATCCTGGCGGTATGGACC--GCTGAGTTTCAGCAAGCCAACGACTACT [illegible]
1_c49      AGCTTTCCAGACTATCGCCGTGTCATTCGCACCTCATACCAAACTCTTCCCCAAGCCGCCTCTAGAATTCTGGCAGTATGGACC--GCTGAGTTTCAACAAGCCAACGACTACT [illegible]
2_c234     ----TTCAAGAATTCTTCCAGCTCAACCGCGAACCTAACTTGGCGGAACTGGAAATGCTTGCTGTAGCTTGTCGTGTACCGATCCAGACGATTGCTGTCTGGTGTAAGTATAC [illegible]
3_c59      AGCTTTCCAGACTATCGCCGTGTCATTCGTACCTCATACCAGACCCTTCCCCAAGCCGCCGCTGGAATCCTGGCAGTATGGACC--GCTGAGTTTCAACAAGCCAACGACTACT [illegible]
4_c69      [illegible]
4_c27      ----TTCAAGAATTCTTCCAGCTCAACCGCGACCCTAACTTGGCAGAACTGGAAATGCTTGCTGTAGCTTGTCGTGTACCGATCCAGACGATTGCTGTCTGGTGTAAGTATAC [illegible]
5_c1       AGCTTTCCAGACTATCGCCGCGTCATTCGCACCTCATACCAGACCCTTCCCCAAGCCGCCGCTGGGATCCTGGCGGTATGGACC--GCTGAGTTTCAGCAAGCCAACGACTACT [illegible]
6_c454     [illegible]
6_c355     ----TTCAAGAATTCTTCCAGCTCAACCGCGAGCCTAACTTGGCAGAACTGGAAATGCTTGCTGTAGCTTGTCGTGTACCGATCCAGACGATTGCTGTCTGGTGTAAGTATAT [illegible]
6_c1860    [illegible]
7_c20      AGCTTCCCAGACTATCGCCGCGTCATTCGCACCTCGTTCCAAACTCTTCCTCAAGCAGCCTTGGGAATCTTAGCAACATGGGGT--TCTGAGTTTCAGCAAGCAAACGACTACT [illegible]
8_c857     AGCTTTCCAGACTATCGCCGTGTCATTCGCACCTCATACCAGACTCTTCCCCAAGCCGCAGCTGGAATTCTGGCAGTATGGACC--GCTGAGTTTCAACAAGCCAGCGATTACT [illegible]
8_c873     ----TTCAAGAATTCTTCCAGCTCAACCGCGAACCTAATTTGGCAGAACTGGAAATGCTTGCTGTAGCTTGTCGTGTACCAGTCCAGACGATTGCTATCTGGTGTAAGTATGC [illegible]
9_c5958    [illegible]
9_c10020   [illegible]
10_c1457   ----TTCAAGAATTCTTCCAGCTCAACCGCGAACCTAACTTGGCGGAACTGGAAATGCTTGCTGTAGCTTGTCGTGTACCGATCCAGACGATTGCTGTCTGGTGTAAGTATAC [illegible]
10_c2708   ----TTCAAGAATTCTTCCAGCTCAACCGCGACCCTAATTTGGCAGAACTGGAAATGCTTGCTGTAGCTTGTCGTGTACCGATCCAGACGATTGCTGTCTGGTGTAAGTATAC [illegible]
11_c14     AGCTTTCCAGACTATCGCCGTGTCATTCGCACCTCATACCAAACTCTTCCCCAAGCCGCCTCTGGAATTCTGGCAGTATGGACT--GCTGAGTTTCAACAAGCCAACGACTACT [illegible]
12_c17     AGCTTTCCAGACTATCGCCGTGTCATTCGCACCTCATACCAAACTCTTCCCCAAGCCGCCTCTGGAATTCTGGCAGTATGGACT--GCTGAGTTTCAACAAGCCAACGACTACT [illegible]
13_c11     AGCTTTCCAGACTATCGCCGTGTCATTCGCACCTCATACCAAACTCTTCCCCAAGCCGCCTCTGGAATTCTGGCAGTATGGACT--GCTGAGTTTCAACAAGCCAACGACTACT [illegible]
14_c36     AGCTTTCCAGACTATCGCCGTGTCATTCGCACCTCATACCAAACTCTTCCCCAAGCCGCCTCTGGAATTCTGGCAGTATGGACT--GCTGAGTTTCAACAAGCCAACGACTACT [illegible]
15_c15     AGCTTTCCAGACTATCGCCGTGTCATTCGCACCTCATACCAAACTCTTCCCCAAGCCGCCTCTGGAATTCTGGCAGTATGGACT--GCTGAGTTTCAACAAGCCAACGACTACT [illegible]
16_c6196   [illegible]
16_c6690   [illegible]
17_c10024  [illegible]
18_c241    AGCTTTCCAGACTATCGCCGTGTCATTCGCACCTCATACCAAACTCTTCCCCAAGCCGCCTCTGGAATTCTGGCAGTATGGACT--GCTGAGTTTCAACAAGCCAACGACTACT [illegible]
19_c9928   [illegible]
20_c9911   [illegible]
21_c10019  [illegible]
22_c9959   [illegible]
23_c9947   [illegible]
24_c9858   [illegible]
25_c29     ----TTCAAGAATTCTTCCAGCTCAACCGCGACCCTAATTTGGCAGAACTGGAAATGCTTGCTGTAGCTTGTCGTGTACCGATCCAGACGATTGCTGTCTGGTGTAAGTATAC [illegible]
26_c4629   [illegible]
26_c4661   [illegible]
27_c7      AGCTTTCCAGACTATCGCCGTGTCATTCGCACCTCATACCAGACCCTTCCCCAAGCCGCCGCTGGGATCCTGGCGGTATGGACC--GCTGAGTTTCAGCAAGCCAACGATTACT [illegible]
28_c3196   [illegible]
28_c4155   [illegible]
28_c2369   AGCTTTCCAGACTATCGCCGTGTCATTCGCACCTCATACCAGACCCTTCCTCAAGCCGCCGCTGGAATTCTGGCAGTATGGACC--GCTGAGTTTCAACAAGCCAACGATTACT [illegible]
29_c1316   AGCTTTCCAGACTATCGCCGTGTCATTCGCACCTCATACCAGACTCTTCCCCAAGCCGCAGCTGGAATTCTGGCAGTATGGACC--GCTGAGTTTCAACAAGCCAACGATTACT [illegible]
29_c4347   [illegible]
30_c2255   AGCTTTCCAGACTATCGCCGTGTCATTCGCACCTCATACCAGACTCTTCCCCAAGCCGCAGCTGGAATTCTGGCAGTATGGACC--GCTGAGTTTCAACAAGCCAACGATTACT [illegible]
30_c4159   [illegible]
30_c5805   [illegible]
31_c9847   [illegible]
32_c4065   [illegible]
32_c2141   AGCTTTCCAGACTATCGCCGTGTCATTCGCACCTCATACCAGACCCTTCCTCAAGCCGCCGCTGGAATTCTGGCAGTATGGACC--GCTGAGTTTCAACAAGCCAACGATTACT [illegible]
32_c5343   [illegible]
33_c6031   [illegible]
34_c9964   [illegible]
36_c454    [illegible]
36_c605    ----TTCAAGAATTCTTCCAGCTCAACCGCGAGCCTAACTTGGCAGAACTGGAAATGCTTGCTGTAGCTTGTCGTGTACCGATCCAGACGATTGCTGTCTGGTGTAAGTATAT [illegible]
37_c2530   AGCTTTCCAGACTATCGCCGTGTCATTCGCACCTCATACCAGACTCTTCCCCAAGCCGCAGCTGGAATTCTGGCAGTATGGACC--GCTGAGTTTCAACAAGCCAACGATTACT [illegible]
37_c9886   [illegible]
38_c721    ----TTCAAGAATTCTTCCAGCTCAACCGCGAACCTAACTTGGCGGAACTGGAAATGCTTGCTGTAGCTTGTCGTGTACCGATCCAGACGATTGCTGTCTGGTGTAAGTATAC [illegible]
40_c13     AGCTTTCCAGACTATCGCCGTGTCATTCGCACCTCATACCAAACTCTTCCCCAAGCCGCCTCTGGAATTCTGGCAGTATGGACT--GCTGAGTTTCAACAAGCCAACGACTACT [illegible]
41_c427    [illegible]
41_c342    ----TTCAAGAATTCTTCCAGCTCAACCGCGAACCTAATTTGGCAGAACTGGAAATGCTTGCTGTAGCTTGTCGTGTACCAGTCCAGACGATTGCTATCTGGTGTAAGTATGC [illegible]
41_c2122   [illegible]
42_c3197   ----TTCAAGAATTCTTCCAGCTCAACCGCGAGCCTAACTTGGCAGAACTGGAAATGCTTGCTGTAGCTTGTCGTGTACCGATCCAGACGATTGCTGTCTGGTGTAAGTATAT [illegible]
42_c3217   AGCTTTCCAGACTATCGCCGTGTCATTCGCACCTCATACCAGACTCTTCCCCAAGCCGCAGCTGGAATTCTGGCAGTATGGACC--GCTGAGTTTCAACAAGCCAACGATTACT [illegible]
43_c9989   [illegible]
44_c12739  [illegible]
44_c12753  [illegible]
45_c2847   AGCTTTCCAGACTATCGCCGTGTCATTCGCACCTCATACCAGACTCTTCCCCAAGCCGCAGCTGGAATTCTGGCAGTATGGACC--GCTGAGTTTCAACAAGCCAACGATTACT [illegible]
45_c13934  [illegible]
45_c13947  [illegible]
46_c12752  [illegible]
46_c12764  [illegible]
47_c9      ----TTCAAGAATTCTTCCAGCTCAACCGCGAACCTAACTTGGCGGAACTGGAAATGCTTGCTGTAGCTTGTCGTGTACCGATCCAGACGATTGCTGTCTGGTGTAAGTATAC [illegible]
48_c18     AGCTTTCCAGACTATCGCCGTGTCATTCGCACCTCATACCAAACTCTTCCCCAAGCCGCCTCTGGAATTCTGGCAGTATGGACC--GCTGAGTTTCAACAAGCCAACGACTACT [illegible]
49_c812    ----TTCAAGAATTCTTCCAGCTCAACCGCGAACCTAACTTGGCGGAACTGGAAATGCTTGCTGTAGCTTGTCGTGTACCGATCCAGACGATTGCTGTCTGGTGTAAGTATAC [illegible]
49_c814    ----TTCAAGAATTCTTCCAGCTCAACCGCGACCCTAATTTGGCAGAACTGGAAATGCTTGCTGTAGCTTGTCGTGTACCGATCCAGACGATTGCTGTCTGGTGTAAGTATAC [illegible]
```

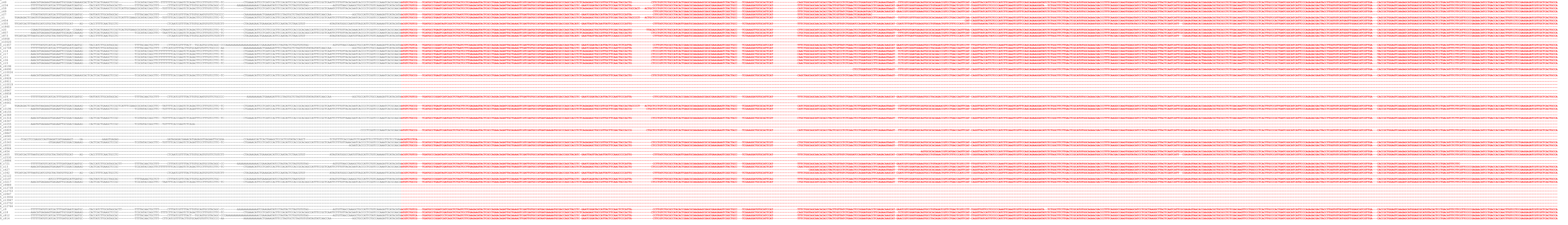

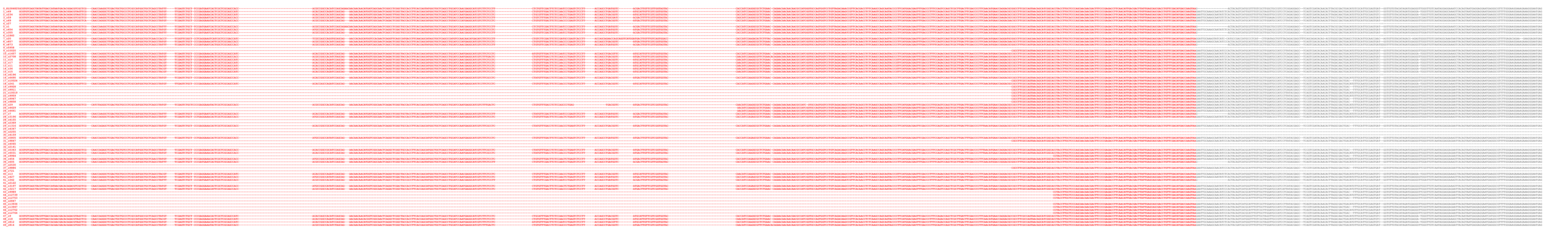

pleckstrin homology domain protein

0_KL584825	[illegible]
1_c49	[illegible]
2_c234	[illegible]
3_c59	[illegible]
4_c69	[illegible]
4_c27	[illegible]
5_c1	[illegible]
6_c454	[illegible]
6_c355	[illegible]
6_c1860	[illegible]
7_c20	[illegible]
8_c857	[illegible]
8_c873	[illegible]
9_c5958	[illegible]
9_c10020	[illegible]
10_c1457	[illegible]
10_c2708	[illegible]
11_c14	[illegible]
12_c17	[illegible]
13_c11	[illegible]
14_c36	[illegible]
15_c15	[illegible]
16_c6196	[illegible]
16_c6690	[illegible]
17_c10024	[illegible]
18_c241	[illegible]
19_c9928	[illegible]
20_c9911	[illegible]
21_c10019	[illegible]
22_c9959	[illegible]
23_c9947	[illegible]
24_c9858	[illegible]
25_c29	[illegible]
26_c4629	[illegible]
26_c4661	[illegible]
27_c7	[illegible]
28_c3196	[illegible]
28_c4155	[illegible]
28_c2369	[illegible]
29_c1316	[illegible]
29_c4347	[illegible]
30_c2255	[illegible]
30_c4159	[illegible]
30_c5805	[illegible]
31_c9847	[illegible]
32_c4065	[illegible]
32_c2141	[illegible]
32_c5343	[illegible]
33_c6031	[illegible]
34_c9964	[illegible]
36_c454	[illegible]
36_c605	[illegible]
37_c2530	[illegible]
37_c9886	[illegible]
38_c721	[illegible]
40_c13	[illegible]
41_c427	[illegible]
41_c342	[illegible]
41_c2122	[illegible]
42_c3197	[illegible]
42_c3217	[illegible]
43_c9989	[illegible]
44_c12739	[illegible]
44_c12753	[illegible]
45_c2847	[illegible]
45_c13934	[illegible]
45_c13947	[illegible]
46_c12752	[illegible]
46_c12764	[illegible]
47_c9	[illegible]
48_c18	[illegible]
49_c812	[illegible]
49_c814	[illegible]

```
0_KL584825CAAACTTGTTCAGAACTTACACTCATCTGACGCTGATGCACATTCGCGCTGGATCAACGCTATAGTCGGCCGTATCTTCCTTGCCATGTACAAGACCAAAGACATCGAGAACTTAATTCGCGCCAAGATTAACAAGAAGATCTCACGCGTAC[illegible]
1_c49     CAAACTTGTACAGAACTTACACTCGTCTGACGCTGATGCACATTCGCGCTGGATTAACGGTATCGTCGGCCGCATTTTTCTTGCCATGTACAAGACCAAAGACATCGAGAACTTCATCCGCGCCAAGATCAACAAGAAGATCTCACGCGTAC[illegible]
2_c234    CAAACTTGTACAGAATTTACACTCGTCTGACGCTGATGCACATTCGCGCTGGATTAACGGTATCGTCGGCCGCATTTTCCTTGCCATGTACAAGACCAAAGACATCGAGAACTTCATCCGCGCCAAGATTAACAAGAAGATCTCACGCGTAC[illegible]
3_c59     CAAACTTGTACAGAACTTACACTCGTCTGATGCCGATGCACATTCTCGCTGGATCAACGCTATCGTCGGCCGTATCTTCCTTGCCATGTACAAGACCAAAGACATTGAAAACTTAATTCGCGCCAAAATTAACAAGAAGATCTCACGCGTAC[illegible]
4_c69     CAAACTTGTACAGAACTTACACTCTTCTGACGCTGATGTACATTCACGCTGGATCAACGGTATCGTCGGTCGTATCTTCCTTGCCATGTACAAGACCAAGGATATCGAAAACTTGATCCGCGCCAAGATTAACAAGAAGATATCACGCGTAC[illegible]
4_c27     ------------------------------------------------------------------------------------------------------------------------------------------------------------[illegible]
5_c1      CAAACTTGTTCAGAACTTACACTCATCTGACGCTGATGCACATTCGCGCTGGATCAACGCTATAGTCGGCCGTATCTTCCTTGCCATGTACAAGACCAAAGACATCGAGAACTTAATTCGCGCCAAGATTAACAAGAAGATCTCACGCGTAC[illegible]
6_c454    CAAACTTGTACAGAACTTACACTCGTCTGACGCTGACGCACATTCGCGCTGGATTAACGGTATCGTCGGCCGCATCTTCCTTGCCATGTACAAGACCAAAGACATCGAAAACTTCATCCGCGCCAAGATTAACAAGAAGATCTCACGCGTAC[illegible]
6_c355    CAAACTTGTACAGAACTTACACTCATCTGACGCTGATGCACATTCGCGCTGGATCAACGCTATAGTCGGCCGTATCTTCCTTGCAATGTACAAGACAAAAGACATCGAGAACTTAATTCGCGCCAAGATTAACAAGAAGATCTCACGCGTAC[illegible]
6_c1860   ------------------------------------------------------------------------------------------------------------------------------------------------------------[illegible]
7_c20     CAAACTTGTACAGAACCTACACTCGTCCGATGCCGACGCTCATTCGCGCTGGATCAACGGCATCGTCGGACGCATCTTTCTTGCCATGTACAAGACCAAAGACATTGAGAACTTCATCCGTGCCAAGATTAACAAGAAGATCTCTCGCGTGC[illegible]
8_c857    CAAACTTGTACAGAACTTACACTCTTCTGACGCTGATGTACATTCACGCTGGATCAACGGTATCGTCGGTCGTATCTTCCTTGCCATGTACAAGACCAAGGATATCGAAAACTTGATCCGCGCCAAGATTAACAAGAAGATATCACGCGTAC[illegible]
8_c873    CAAACTTGTACAGAACTTACACTCATCTGACGCTGATGCACATTCGCGCTGGATCAACGCTATAGTCGGCCGTATCTTCCTTGCAATGTACAAGACAAAAGACATCGAGAACTTAATTCGCGCCAAGATTAACAAGAAGATCTCACGCGTAC[illegible]
9_c5958   ------------------------------------------------------------------------------------------------------------------------------------------------------------[illegible]
9_c10020  CAAACTTGTACA------------------------------------------------------------------------------------------------------------------------------------------------[illegible]
10_c1457  CAAACTTGTACAGAATTTACACTCGTCTGACGCTGATGCACATTCGCGCTGGATTAACGGTATCGTCGGCCGCATTTTCCTTGCCATGTACAAGACCAAAGACATCGAGAACTTCATCCGCGCCAAGATTAACAAGAAGATCTCACGCGTAC[illegible]
10_c2708  CAAACTTGTACAGAACTTACACTCTTCTGACGCTGATGCACATTCACGCTGGATCAACGGTATCGTCGGTCGTATCTTCCTTGCCATGTACAAGACCAAAGACATCGAAAACTTGATCCGCGCCAAGATTAACAAGAAAATATCACGCGTAC[illegible]
11_c14    CAAACTTGTACAGAACTTACACTCGTCTGATGCTGATGCACATTCGCGCTGGATTAACGGTATCGTCGGCCGCATTTTTCTTGCCATGTACAAGACCAAAGACATCGAGAACTTCATCCGCGCCAAGATTAACAAGAAGATCTCACGCGTAC[illegible]
12_c17    CAAACTTGTACAGAACTTACACTCGTCTGATGCTGATGCACATTCGCGCTGGATTAACGGTATCGTCGGCCGCATTTTTCTTGCCATGTACAAGACCAAAGACATCGAGAACTTCATCCGCGCCAAGATTAACAAGAAGATCTCACGCGTAC[illegible]
13_c11    CAAACTTGTACAGAACTTACACTCGTCTGATGCTGATGCACATTCGCGCTGGATTAACGGTATCGTCGGCCGCATTTTTCTTGCCATGTACAAGACCAAAGACATCGAGAACTTCATCCGCGCCAAGATTAACAAGAAGATCTCACGCGTAC[illegible]
14_c36    CAAACTTGTACAGAACTTACACTCGTCTGATGCTGATGCACATTCGCGCTGGATTAACGGTATCGTCGGCCGCATTTTTCTTGCCATGTACAAGACCAAAGACATCGAGAACTTCATCCGCGCCAAGATTAACAAGAAGATCTCACGCGTAC[illegible]
15_c15    CAAACTTGTACAGAACTTACACTCGTCTGATGCTGATGCACATTCGCGCTGGATTAACGGTATCGTCGGCCGCAT[illegible]
16_c6196  ---------------------------------------------------------------------------[illegible]
16_c6690  CAAACTTGTACA---------------------------------------------------------------[illegible]
17_c10024 CAAACTTGTACA---------------------------------------------------------------[illegible]
18_c241   CAAACTTGTACAGAACTTACACTCGTCTGATGCTGATGCACATTCGCGCTGGATTAACGGTATCGTCGGCCGCAT[illegible]
19_c9928  CAAACTTGTACA---------------------------------------------------------------[illegible]
20_c9911  CAAACTTGTACA---------------------------------------------------------------[illegible]
21_c10019 CAAACTTGTACA---------------------------------------------------------------[illegible]
22_c9959  CAAACTTGTACA---------------------------------------------------------------[illegible]
23_c9947  CAAACTTGTACA---------------------------------------------------------------[illegible]
24_c9858  CAAACTTGTACA---------------------------------------------------------------[illegible]
25_c29    CAAACTTGTACAGAACTTACACTCTTCTGACGCTGATGCACATTCACGCTGGATCAACGGTATCGTCGGTCGTAT[illegible]
26_c4629  CAAACTTGTACAGAACTTACACTCGTCTGATGCTGATGCACATTCGCGCTGGATTAACGGTATCGTCGGCCGCAT[illegible]
26_c4661  CAAACTTGTACAAAACTTACACTCTTCTGACGCTGATGCACATTCGCGCTGGATCAACGGTATCGTCGGTCGTAT[illegible]
27_c7     CAAACTTGTACAGAACTTACACTCATCTGACGCTGATGCACATTCGCGCTGGATCAACGCTATAGTCGGCCGTAT[illegible]
28_c3196  CAAACTTGTACAAAACTTACACTCTTCTGACGCTGATGCACATTCGCGCTGGATCAACGGTATCGTCGGTCGTAT[illegible]
28_c4155  ---------------------------------------------------------------------------[illegible]
28_c2369  ---------------------------------------------------------------------------[illegible]
29_c1316  ---------------------------------------------------------------------------[illegible]
29_c4347  ---------------------------------------------------------------------------[illegible]
30_c2255  ---------------------------------------------------------------------------[illegible]
30_c4159  ---------------------------------------------------------------------------[illegible]
30_c5805  ---------------------------------------------------------------------------[illegible]
31_c9847  CAAACTTGTACA---------------------------------------------------------------[illegible]
32_c4065  ---------------------------------------------------------------------------[illegible]
32_c2141  ---------------------------------------------------------------------------[illegible]
32_c5343  ---------------------------------------------------------------------------[illegible]
33_c6031  CAAACTTGTACA---------------------------------------------------------------[illegible]
34_c9964  CAAACTTGTACA---------------------------------------------------------------[illegible]
36_c454   CAAACTTGTACAGAACTTACACTCGTCTGACGCTGACGCACATTCGCGCTGGATTAACGGTATCGTCGGCCGCAT[illegible]
36_c605   CAAACTTGTACAGAACTTACACTCATCTGACGCTGATGCACATTCGCGCTGGATCAACGCTATAGTCGGCCGTAT[illegible]
37_c2530  ---------------------------------------------------------------------------[illegible]
37_c9886  CAAACTTGTACA---------------------------------------------------------------[illegible]
38_c721   ---------------------------------------------------------------------------[illegible]
40_c13    CAAACTTGTACAGAACTTACACTCGTCTGATGCTGATGCACATTCGCGCTGGATTAACGGTATCGTCGGCCGCAT[illegible]
41_c427   CAAACTTGTACAGAACTTACACTCGTCTGACGCTGACGCACATTCGCGCTGGATTAACGGTATCGTCGGCCGCAT[illegible]
41_c342   CAAACTTGTACAGAACTTACACTCATCTGACGCTGATGCACATTCGCGCTGGATCAACGCTATAGTCGGCCGTAT[illegible]
41_c2122  ---------------------------------------------------------------------------[illegible]
42_c3197  ---------------------------------------------------------------------------[illegible]
42_c3217  ---------------------------------------------------------------------------[illegible]
43_c9989  CAAACTTGTACA---------------------------------------------------------------[illegible]
44_c12739 ---------------------------------------------------------------------------[illegible]
44_c12753 ---------------------------------------------------------------------------[illegible]
45_c2847  ---------------------------------------------------------------------------[illegible]
45_c13934 ---------------------------------------------------------------------------[illegible]
45_c13947 ---------------------------------------------------------------------------[illegible]
46_c12752 ---------------------------------------------------------------------------[illegible]
46_c12764 ---------------------------------------------------------------------------[illegible]
47_c9     CAAACTTGTACAGAACTTACACTCGTCTGATGCTGATGCACATTCGCGCTGGATTAACGGTATCGTCGGCCGCAT[illegible]
48_c18    CAAACTTGTACAGAACTTACACTCGTCTGATGCTGATGCACATGCGCGCTGGATTAACGGTATCGTCGGTCGCAT[illegible]
49_c812   CAAACTTGTACAGAATTTACACTCGTCTGACGCTGATGCACATTCGCGCTGGATTAACGGTATCGTCGGCCGCAT[illegible]
49_c814   CAAACTTGTACAGAACTTACACTCTTCTGACGCTGATGCACATTCACGCTGGATCAACGGTATCGTCGGTCGTAT[illegible]
```

```
0_KL584825 [illegible]
1_c49 [illegible]
2_c234 [illegible]
3_c59 [illegible]
4_c69 [illegible]
4_c27 [illegible]
5_c1 [illegible]
6_c454 [illegible]
6_c355 [illegible]
6_c1860 [illegible]
7_c20 [illegible]
8_c857 [illegible]
8_c873 [illegible]
9_c5958 [illegible]
9_c10020 [illegible]
10_c1457 [illegible]
10_c2708 [illegible]
11_c14 [illegible]
12_c17 [illegible]
13_c11 [illegible]
14_c36 [illegible]
15_c15 [illegible]
16_c6196 [illegible]
16_c6690 [illegible]
17_c10024 [illegible]
18_c241 [illegible]
19_c9928 [illegible]
20_c9911 [illegible]
21_c10019 [illegible]
22_c9959 [illegible]
23_c9947 [illegible]
24_c9858 [illegible]
25_c29 [illegible]
26_c4629 [illegible]
26_c4661 [illegible]
27_c7 [illegible]
28_c3196 [illegible]
28_c4155 [illegible]
28_c2369 [illegible]
29_c1316 [illegible]
29_c4347 [illegible]
30_c2255 [illegible]
30_c4159 [illegible]
30_c5805 [illegible]
31_c9847 [illegible]
32_c4065 [illegible]
32_c2141 [illegible]
32_c5343 [illegible]
33_c6031 [illegible]
34_c9964 [illegible]
36_c454 [illegible]
36_c605 [illegible]
37_c2530 [illegible]
37_c9886 [illegible]
38_c721 [illegible]
40_c13 [illegible]
41_c427 [illegible]
41_c342 [illegible]
41_c2122 [illegible]
42_c3197 [illegible]
42_c3217 [illegible]
43_c9989 [illegible]
44_c12739 [illegible]
44_c12753 [illegible]
45_c2847 [illegible]
45_c13934 [illegible]
45_c13947 [illegible]
46_c12752 [illegible]
46_c12764 [illegible]
47_c9 [illegible]
48_c18 [illegible]
49_c812 [illegible]
49_c814 [illegible]
```

```
0_KL584825GGATTGGAG---CAACAAGGAGCTGGTGATCCCGACTTGTTAGTCGTCGCTGCTCCTGTGGAGGACGATAGTATG [illegible]
1_c49     GGCCTGGAG---CAACAAGTAGCTGGTGATCCCGATCTGTTAGTCGTCGCTGCTCCTGTAGAGGACGATAGTATG [illegible]
2_c234    GGCCTGGAG---CAACAAGTAGCTGGTGATCCCGACCTGTTAGTCGTCGCTGCTCCTGTAGAGGACGATAGTATG [illegible]
3_c59     GGACTGGAGCAACAACAAATAGCTCTTGATCCCGACTTGTTAGTCGTCGCTGCTCCTGTAGAGGACGATAGTATG [illegible]
4_c69     GGCCTGGAG---CAACAAGTAGCTGGTGATCCCGACTTGTTGGTCGTCGCTGCTCCTGTAGAGGACGATAGCATG [illegible]
4_c27     ---------------------------------------------------------------------------- [illegible]
5_c1      GGATTGGAG---CAACAAGGAGCTGGTGATCCCGACTTGTTAGTCGTCGCTGCTCCTGTGGAGGACGATAGTATG [illegible]
6_c454    GCATTGGAGCAACAACAAGTAGCTGGTGATCCTGACTTGTTAGTGGTCGCTGCTCCTGTAGAGGACGATAGTATG [illegible]
6_c355    GGATTGGAG---CAACAAGTAGCTGGTGATCCCGACTTGTTAGTCGTCGCTGCTCCTGTGGAGGACGATAGTATG [illegible]
6_c1860   ---------------------------------------------------------------------------- [illegible]
7_c20     AGATCGGAA---CAGCAAGCAATTGGGGACCCCGATGTGTTTGTTGTTGCTGCCCCTGTGGAGGATGATAGTATG [illegible]
8_c857    GGCCTGGAG---CAACAAGTAGCTGGTGATCCCGACTTGTTGGTCGTCGCTGCTCCTGTAGAGGACGATAGCATG [illegible]
8_c873    GGATTGGAG---CAACAAGTAGCTGGTGATCCCGACTTGTTAGTCGTCGCTGCTCCTGTGGAGGACGATAGTATG [illegible]
9_c5958   ---------------------------------------------------------------------------- [illegible]
9_c10020  ---------------------------------------------------------------------------- [illegible]
10_c1457  GGCCTGGAG---CAACAAGTAGCTGGTGATCCCGACCTGTTAGTCGTCGCTGCTCCTGTAGAGGACGATAGTATG [illegible]
10_c2708  ---------------------------------------------------------------------------- [illegible]
11_c14    GGCCTGGAG---CAACAAGTAGCTGGTGATCCCGATCTGTTAGTCGTCGCTGCTCCTGTAGAGGACGATAGTATG [illegible]
12_c17    GGCCTGGAG---CAACAAGTAGCTGGTGATCCCGATCTGTTAGTCGTCGCTGCTCCTGTAGAGGACGATAGTATG [illegible]
13_c11    GGCCTGGAG---CAACAAGTAGCTGGTGATCCCGATCTGTTAGTCGTCGCTGCTCCTGTAGAGGACGATAGTATG [illegible]
14_c36    GGCCTGGAG---CAACAAGTAGCTGGTGATCCCGATCTGTTAGTCGTCGCTGCTCCTGTAGAGGACGATAGTATG [illegible]
15_c15    GGCCTGGAG---CAACAAGTAGCTGGTGATCCCGATCTGTTAGTCGTCGCTGCTCCTGTAGAGGACGATAGTATG [illegible]
16_c6196  ---------------------------------------------------------------------------- [illegible]
16_c6690  ---------------------------------------------------------------------------- [illegible]
17_c10024 ---------------------------------------------------------------------------- [illegible]
18_c241   GGCCTGGAG---CAACAAGTAGCTGGTGATCCCGACCTGTTAGTCGTCGCTGCTCCTGTAGAGGACGATAGTATG [illegible]
19_c9928  ---------------------------------------------------------------------------- [illegible]
20_c9911  ---------------------------------------------------------------------------- [illegible]
21_c10019 ---------------------------------------------------------------------------- [illegible]
22_c9959  ---------------------------------------------------------------------------- [illegible]
23_c9947  ---------------------------------------------------------------------------- [illegible]
24_c9858  ---------------------------------------------------------------------------- [illegible]
25_c29    GGCCTGGAG---CAACAAGCAGCTGGTGATCCCGACTTGTTGGTCGTCGCTGCTCCTGTAGAGGACGATAGCATG [illegible]
26_c4629  ---------------------------------------------------------------------------- [illegible]
26_c4661  ---------------------------------------------------------------------------- [illegible]
27_c7     GGACTGGAGCAACAACAAGTAGCTGTTGATCCCGACTTGTTAGTCGTCGCTGCTCCTGTAGAGGACGATAGTATG [illegible]
28_c3196  ---------------------------------------------------------------------------- [illegible]
28_c4155  ---------------------------------------------------------------------------- [illegible]
28_c2369  ---------------------------------------------------------------------------- [illegible]
29_c1316  ---------------------------------------------------------------------------- [illegible]
29_c4347  ---------------------------------------------------------------------------- [illegible]
30_c2255  ---------------------------------------------------------------------------- [illegible]
30_c4159  ---------------------------------------------------------------------------- [illegible]
30_c5805  ---------------------------------------------------------------------------- [illegible]
31_c9847  ---------------------------------------------------------------------------- [illegible]
32_c4065  ---------------------------------------------------------------------------- [illegible]
32_c2141  ---------------------------------------------------------------------------- [illegible]
32_c5343  ---------------------------------------------------------------------------- [illegible]
33_c6031  ---------------------------------------------------------------------------- [illegible]
34_c9964  ---------------------------------------------------------------------------- [illegible]
36_c454   GCATTGGAGCAACAACAAGTAGCTGGTGATCCTGACTTGTTAGTGGTCGCTGCTCCTGTAGAGGACGATAGTATG [illegible]
36_c605   GGATTGGAG---CAACAAGTAGCTGGTGATCCCGACTTGTTAGTCGTCGCTGCTCCTGTGGAGGACGATAGTATG [illegible]
37_c2530  ---------------------------------------------------------------------------- [illegible]
37_c9886  ---------------------------------------------------------------------------- [illegible]
38_c721   ---------------------------------------------------------------------------- [illegible]
40_c13    GGCCTGGAG---CAACAAGTAGCTGGTGATCCCGATCTGTTAGTCGTCGCTGCTCCTGTAGAGGACGATAGTATG [illegible]
41_c427   GCATTGGAGCAACAACAAGTAGCTGGTGATCCTGACTTGTTAGTGGTCGCTGCTCCTGTAGAGGACGATAGTATG [illegible]
41_c342   GGATTGGAG---CAACAAGTAGCTGGTGATCCCGACTTGTTAGTCGTCGCTGCTCCTGTGGAGGACGATAGTATG [illegible]
41_c2122  ---------------------------------------------------------------------------- [illegible]
42_c3197  ---------------------------------------------------------------------------- [illegible]
42_c3217  ---------------------------------------------------------------------------- [illegible]
43_c9989  ---------------------------------------------------------------------------- [illegible]
44_c12739 ---------------------------------------------------------------------------- [illegible]
44_c12753 ---------------------------------------------------------------------------- [illegible]
45_c2847  ---------------------------------------------------------------------------- [illegible]
45_c13934 ---------------------------------------------------------------------------- [illegible]
45_c13947 ---------------------------------------------------------------------------- [illegible]
46_c12752 ---------------------------------------------------------------------------- [illegible]
46_c12764 ---------------------------------------------------------------------------- [illegible]
47_c9     GGCCTGGAG---CAACAAGTAGCTGGTGATCCCGACCTGTTGGTCGTCGCTGCTCCTGTAGAGGACGATAGTATG [illegible]
48_c18    GGCCTGGAG---CAACAAGTAGCTGGTGATCCCGACCTGTTAGTCGTCGCTGCTCCTGTAGAGGACGATAGTATG [illegible]
49_c812   GGCCTGGAG---CAACAAGTAGCTGGTGATCCCGACCTGTTAGTCGTCGCTGCTCCTGTAGAGGACGATAGTATG [illegible]
49_c814   GGCCTGGAG---CAACAAGCAGCTGGTGATCCCGACTTGTTGGTCGTCGCTGCTCCTGTAGAGGACGATAGCATG [illegible]
```
