# Supplementary figures and images for: Clonality, inbreeding, and hybridization in two extremotolerant black yeasts
Source: Gigascience. 2022 Oct 6;11:giac095. doi: 10.1093/gigascience/giac095 (PMC9535773; doi:10.1093/gigascience/giac095)

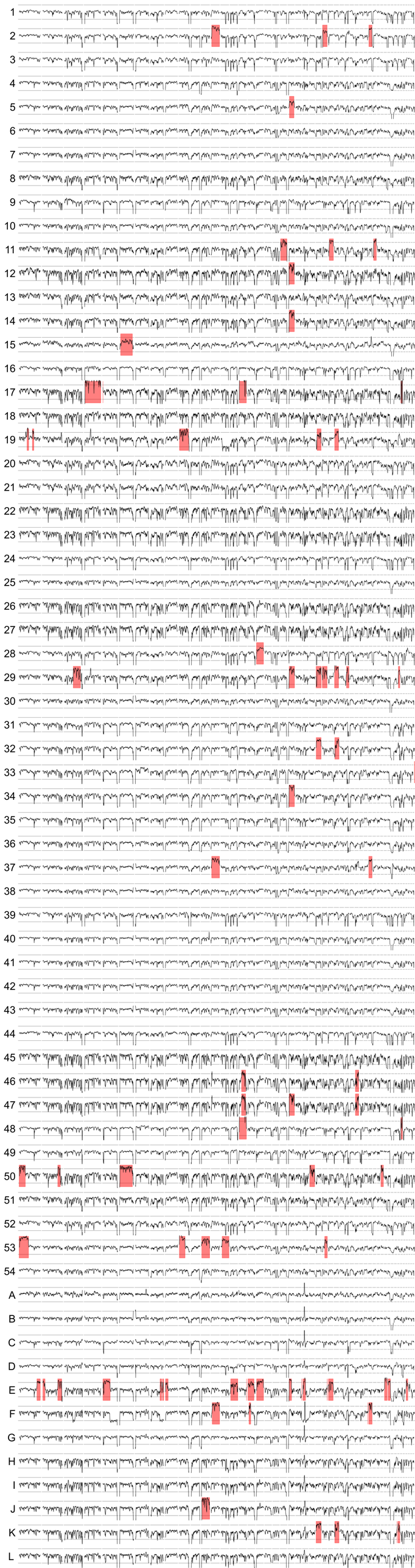

Supplement: giac095_Supplemental_Files [file giac095_supplemental_files.zip › FigS1.png]

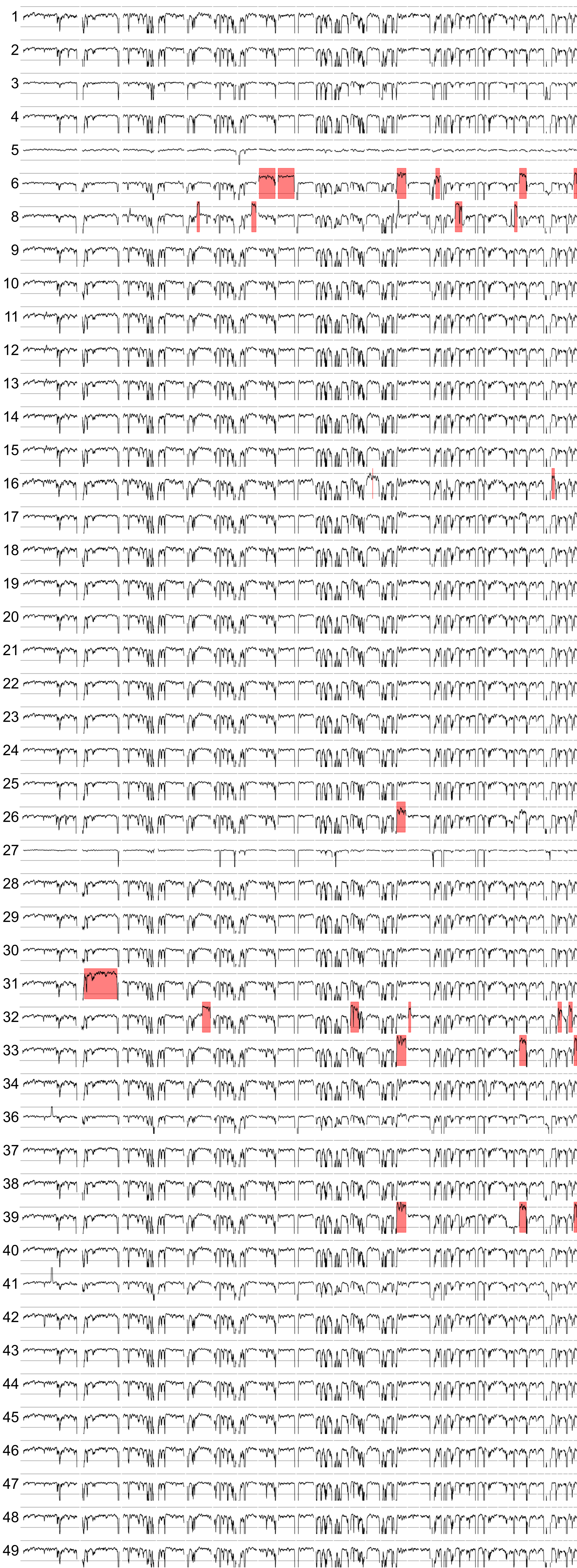

Supplement: giac095_Supplemental_Files [file giac095_supplemental_files.zip › FigS2.png]

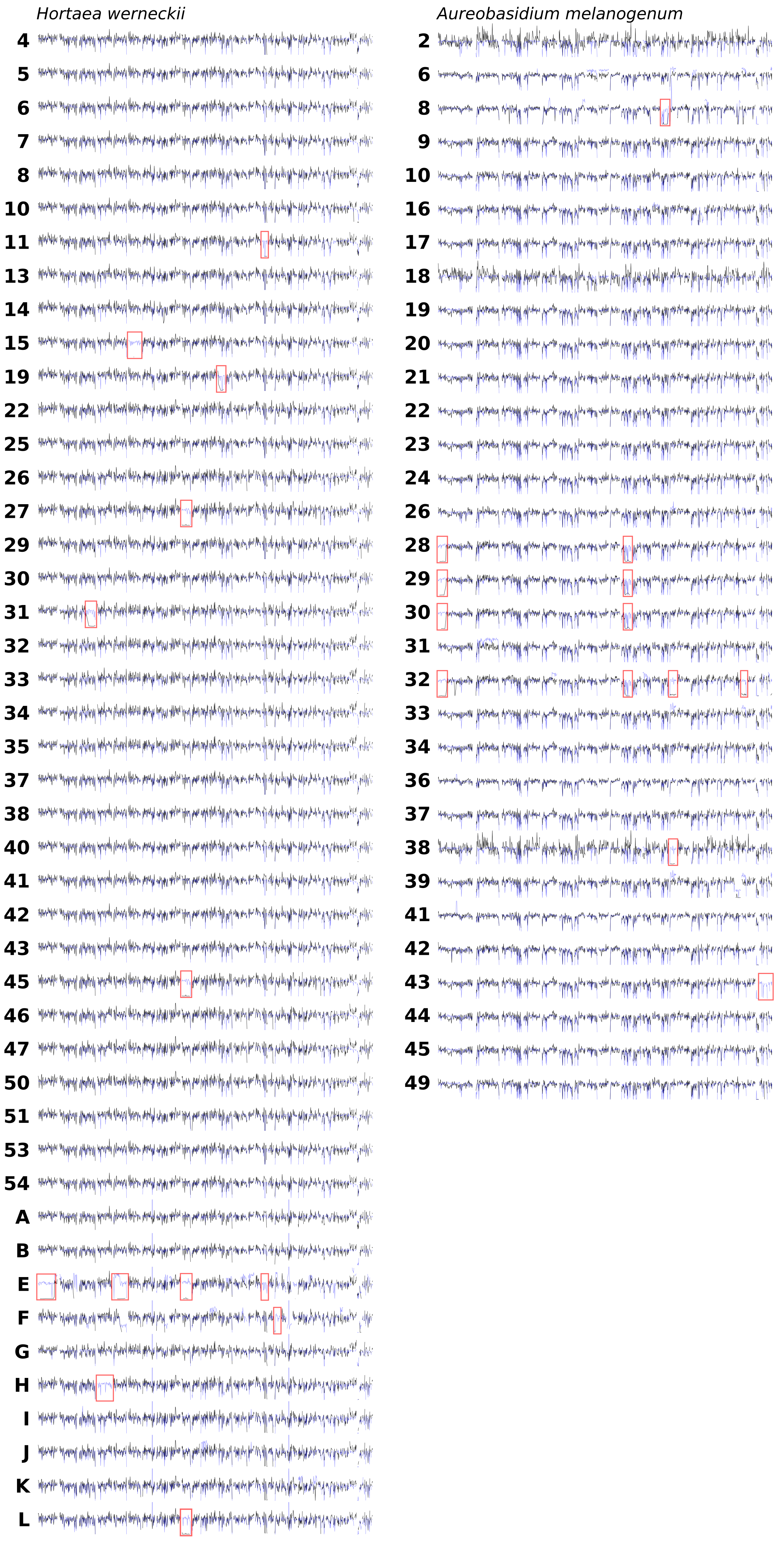

Supplement: giac095_Supplemental_Files [file giac095_supplemental_files.zip › FigS3.png]
