# Supplementary material for: Clonality, inbreeding, and hybridization in two extremotolerant black yeasts
Source: Gigascience. 2022 Oct 6;11:giac095. doi: 10.1093/gigascience/giac095 (PMC9535773; doi:10.1093/gigascience/giac095)
Supplement: giac095_Response_to_Reviewer_Comments_Original_Submission [file giac095_response_to_reviewer_comments_original_submission.pdf]

Author's Response To Reviewer Comments

Close

We would like to thank the reviewers for their detailed and constructive feedback! Both reviewers requested revisions, which have now been made. The detailed responses to the comments of the reviewers are listed below. We believe this strengthens the manuscript and we hope you can consider it for publication.

Reviewer #1

Comment: separate trees were generated for "longest alignments" and I think this approach can hide potential admixture events. It is not reported anywhere what are the average lengths of these alignments, but the point is that if admixture concerns a small part of the chromosome, the alignment of the whole chromosome will not detect those admixture events. I would suggest to split all alignments into portions of equal length or of equal number of informative SNPs, to identify potential admixture events, if any exist.
Answer: The range of lengths of the alignments is now reported in the methodology: “lengths of 1364 bp to 5089 bp for H. werneckii and 3400 bp to 13257 bp for A. melanogenum”. The length of these alignments was not overwhelming, therefore the masking of the admixture events was not explicitly addressed in the initial manuscript – they should be able to reflect also admixture events concerning only small parts of the chromosome. If the alignments were split further, the number of phylogenetically informative sites in them would be limited. The divergence of the genomes and likely also the difficult assembly of diploid genomes precluded the recovery of longer alignments with a 100% representation of all haploid genomes. However, as an alternative to the alignment-splitting approach, phylogenetic trees were also constructed from core BUSCOs that were found in all genomes of each species (and in the number of copies corresponding to the ploidy of the genome). Since the number of genomes was considerable and since an unexpected copy number in even a single genome excluded the gene from the analysis, the final dataset was fairly modest. Alignments longer than 200 nucleotides and with an average of at least 15 nucleotide differences between gene pairs were used for phylogeny reconstruction, producing 22 phylogenetic trees in case of H. werneckii and 44 in case of A. melanogenum. While these phylogenies are generally based on shorter alignments and are perhaps less reliable (which was the reason for reporting Sibelia and not BUSCO alignments in the manuscript), they result in almost identical topology and the proportion of trees supporting the major splits are very similar. Please see the here provided phylogeny with percentages to the right of trees marking the proportion of trees supporting the major clusters. Black percentage points are now reported in the revised Fig. 3 (in response to the comment below; calculated from phylogenies based on genomic alignments produced by Sibelia). Red numbers are the percentages of trees supporting the same clusters in trees produced from alignment of BUSCOs (provided here for review purposes only).
[please refer to the attached file Answers.to.reviewers.REVIEW.ONLY.docx for the image]

Comment: tree discordancies are not quantified in any way and from figure 3 it's hard to judge how much concordance there is. If the species cluster in several groups one could show how many topologies (proportion) of these major clusters are consistent with each other and how many are different
Answer: The proportion of phylogenies supporting the major clusters have now been labelled in panels A and B of Fig. 3 as suggested. The figure legend has been amended accordingly.

Minor comments:
Comment: Table S1 and Table 1: Please explain in the legend what distributions are showing. Also it would be helpful to include a column in the tables with information about ploidy.
Answer: The legend of Table S1 (which shows the distributions) has been amended to explain the plots in more detail. The column reporting the ploidy has been added as suggested to Table 1 and Table S1. Additionally, the ploidy column has been also added to Table 2 to keep the format of tables consistent.
Comment: line 183: I'm not sure what the authors mean by 'consistent' in this sentence. Wasn't the

ploidy decided from genome assembly characteristics? In this case it's expected to be consistent.
Answer: This was an awkwardly written sentence – we did not want to say that the genomic characteristics were consistent with ploidy (which they were, because this is how the ploidy was determined, as correctly pointed out by the reviewer), but that haploid strains were very similar in their characteristics to each other, and the same was true for diploid strains. We rewrote the sentence as follows and hopefully this makes the message clearer: "The distribution of assembly size, number of predicted genes and other genomic characteristics within both haploid and diploid H. werneckii groups was narrow (Table 3).".
Comment: line 195: I would suggest explaining here in once sentence how SNP calling was made, especially how the reference was constructed, because it's quite important for interpreting the results.
Answer: We added a short description, as suggested: "Single-nucleotide polymorphisms (SNPs) were determined with Genome Analysis Toolkit after mapping the sequencing reads to reference genomes (haploidised genome of diploid strain EXF-2000 in case of H. werneckii, whole genome of haploid strain EXF-3378 in case of A. melanogenum)." We hope this is sufficiently detailed for the Results section, since a more detailed description is provided in the Methods.
Comment: line 202: PCA plots in Fig1: It would be useful to add ploidy information in the plot to see where are those samples located relative to haploids. Are these clusters explained by geography or habitat? Perhaps adding this information would be useful as well. It is also not mentioned anywhere how divergent are these clusters. It would be worth reporting nucleotide divergence between (haploid) genomic groups.
Answer: Diploid strains have now been marked with bold labels in all panels of Fig. 1 – PCA plots as suggested, but also in phylogenetic networks. The sizes and placement of labels have been optimized to increase legibility. The clusters can indeed be partially explained by geography/habitat – these information are visualised in Fig. 4 and discussed there as well. This decision was made after we first attempted to show the geography/habitat data in Fig. 1 (PCA plots) and discovered that the number of genomes and their considerable overlapping make an efficient visualisation all but impossible. Divergence between clusters is now also marked in Fig. 1 with dashed lines marking the groups of haploid strains and distance in millions of SNPs between groups. The legend of the figure has been amended to reflect these changes.
Comment: line 221: Please highlight haploids/diploid on the phylogeny.
Answer: In Fig. 3, ploidy has now been marked on the phylogeny – diploid strain names have been written in bold in panels C and D. In panels A and B diploid (and tetraploid) strains were already marked with letters added to strain names – this has now been explicitly explained in the legend as well.
Comment: line 209: Considering LD decay analysis it looks to me that r2 is very low even between close variants. In general, it is not clear from the figure 2 what is the maximum r2 between adjacent pairs of SNPs (start of the line) and what is the distance over which r2 falls by half. I think the authors should give some quantification of this in the results. This could give a better understanding of the LD.
Answer: The maximum value of LD (drawn as the upper red horizontal line on the LD charts) are now also reported in the Results, as suggested: "Plotting r2 as a function of the distance between pairs of loci showed very little decay of linkage disequilibrium in either species from the maximum initial values of 0.17 for H. werneckii and 0.22 for A. melanogenum.". Regarding the low values, the initial r2 value is not only dependent on the disequilibrium, but also on the allele frequency (i.e. alleles in less than 50% frequency that are in total disequilibrium, will have r2 not 1 but less than 1) and is therefore not unusual.
Comment: line 229: This is a really interesting way to show relationships between hybrids!
Answer: Thank you!
Comment: line 250: Fig 5, What are colours in the legend signifying?
Answer: The sentence explaining the colours was lost in revision and was now again added: "Colours of strain names in the legend mark haploid (blue) and diploid (red) genomes." Thank you for alerting us to this!
Comment: line 282: MAT loci: One option to make sure if MAT loci is truly absent would be to look for reads matching the sequence of MAT. This could eliminate the possibility that the quality of an assembly is a source of missing loci.
Answer: The original manuscript already acknowledged the possibility that the apparently missing loci are the consequence of the assembly problems and not of their true absence. We now performed an additional analysis, as suggested. In many cases this was not helpful because the divergence of some loci is so large, that it resulted in gaps in the sequencing coverage even in cases where the putative mating-type loci were found in the assembly. In other cases, especially in A. melanogenum, the coverage did indeed suggest, as the reviewer thought it might, that the loci are actually present in the genome, but not present in the genome assembly. The results of the sequencing coverage analysis have been added to Supplementary Figs. 4 and 5 and the corresponding figure legends amended as appropriate. The corresponding Results section of the manuscript has been changed as follows: "While

mapping of sequencing reads of some strains to the reference mating-type locus of each species contained gaps due to high divergence of the locus, in some other strains the sequencing coverage indicated the presence of the locus despite its absence in the whole-genome assembly (Supplemental Figs. S4, S5). The poor assembly of the locus was particularly problematic in diploid genomes and even more so in the tetraploid genome of H. werneckii, precluding a conclusive analysis in all strains. The absence of the locus in some strains should at this point in time not be seen as a conclusive result."
Our opinion on this is that the sequencing coverage analysis comes with its own set of problems, but its inclusion does indeed provide a more complete picture of a complex situation. While a more conclusive resolution of this question can only be provided by using a sequencing technology producing longer reads, the results presented in this manuscript, while partial, do provide relevant information tightly linked to the main topic of the manuscript as well as important indications for future research, at the same time acknowledging the partiality of the results.
Comment: line 263: In the figures S4 and S5 one information that is missing is whether the same MAT type is present always on the same genomic background, assuming that these different types are in the same locus. Examples of MAT introgression are common in fungi so it would be nice to check if something like this occurs in these species.
Answer: We agree that this is an interesting question and we did some preliminary investigation into it before preparing the manuscript. However, due to the above discussed suboptimal assembly in the mating-type loci, this was only possible for some strains. Additionally, the precise borders of the mating locus in H. werneckii are not known. Based on all of this we decided not to report highly uncertain data and rather leave this question to be solved by a more targeted experiment, e.g. by using a sequencing technology producing long reads.
Comment: line 619: In the description of processing sequence alignments, please specify what do you mean by "long gaps"? Was there any threshold?
Answer: This was indeed not as precise as it should be. We replaced "long gaps" with "with more than 15% gaps over the whole alignment length in any of the sequences of H. werneckii or 25% in case of A. melanogenum".

Reviewer #2

Major:
Comment: The methods are missing a description of how ploidy was estimated, the results of which are integral to the conclusions.
Answer: Thank you for pointing this out. To correct this omission, the methods have been amended with the following paragraph: "The ploidy of the genomes was determined based on the following criteria for both species: haploids had a genome size smaller than 31 Mbp, number of predicted genes smaller than 13 000 and the average copy number of core BUSCOs (those present in all strains of the species) lower than 1.1. Diploid strains had a genome size larger than 46 Mbp, number of predicted genes greater than 18 000 and the average copy number of core BUSCOs greater than 1.5. The ploidy of genomes with any of the criteria between the above thresholds was labelled as "unclear" (Table 2).".

Comment: The authors should be clearer about how they use and what they mean by clonality and asexuality particularly with respect to recombination.
Answer: We carefully re-read the manuscript to make the use of terminology unambiguous. We use the term "asexuality" sparsely and only in the introduction, when referencing to the works of other authors, which wrote about asexuality in the sense of the absence of conventional sexual reproduction in fungi. In other parts of the manuscript we use the term "clonality" – reproduction, which produces clonal offspring. While the absence of sexual reproduction still allows for recombination (e.g. through parasexuality), the term clonality implies the absence of recombination altogether – something that is supported by genomic data for both H. werneckii and A. melanogenum (with the exception of intraspecific hybrids). We also added the short definition of clonality in the beginning of the introduction to indicate how the term is used in the manuscript: "Among the most diverse are fungi, which exhibit a wide range of strategies, from strictly clonal species, which do not recombine at all [1], to species with thousands of mating types [2]." We hope this provides the requested clarity.

Minor:
Comment: The use of the term 'hybrid' should be preceded by the descriptor 'intraspecific' for clarity's sake
Answer: We have added the descriptor "interspecific" to the manuscript, as suggested.

Comment: A clearer description of the question/hypotheses being addressed earlier in the introduction would go a long way to improve readability.
Answer: As suggested, the hypothesis has now been briefly introduced at the end of the second paragraph of the Introduction: “Yet some species appear to be strictly clonal, even by highly sensitive measures of recombination used by population genomics, such as linkage disequilibrium [1,8]. This study focuses on two species of such strictly clonal fungi. The analysis of 115 genomes of haploid and diploid wild strains is used to test the hypothesis that even clonal phylogenetic lineages can generate diversity through hybridization that produces highly heterozygous and stable diploids.”.

Close
