# Supplementary material for: Clonality, inbreeding, and hybridization in two extremotolerant black yeasts
Source: Gigascience. 2022 Oct 6;11:giac095. doi: 10.1093/gigascience/giac095 (PMC9535773; doi:10.1093/gigascience/giac095)
Supplement: giac095_Reviewer_1_Report_Original_Submission — Anna Fijarczyk -- 5/12/2022 Reviewed [file giac095_reviewer_1_report_original_submission.pdf]

Reviewer Report

Title: Clonality, inbreeding, and hybridization in two extremotolerant black yeasts

Version: Original Submission **Date:** 5/12/2022

Reviewer name: Anna Fijarczyk

Reviewer Comments to Author:

In this manuscript authors analyse whole genome sequences of two extremotolerant black yeast species (66 and 49 respectively). Authors demonstrate that several diploid samples are derived from hybridization events and argue that genomes remain clonal. This is definitely a very intriguing study system, and I like that different aspects of genetic variation are investigated and discussed. I have a few suggestions about the analyses in support of clonality and presentation of the results.

Considering the analysis of tree concordance I have two comments. First, separate trees were generated for "longest alignments" and I think this approach can hide potential admixture events. It is not reported anywhere what are the average lengths of these alignments, but the point is that if admixture concerns a small part of the chromosome, the alignment of the whole chromosome will not detect those admixture events. I would suggest to split all alignments into portions of equal length or of equal number of informative SNPs, to identify potential admixture events, if any exist. My second comment is that tree discordancies are not quantified in any way and from figure 3 it's hard to judge how much concordance there is. If the species cluster in several groups one could show how many topologies (proportion) of these major clusters are consistent with each other and how many are different.

Minor comments:

Table S1 and Table 1: Please explain in the legend what distributions are showing. Also it would be helpful to include a column in the tables with information about ploidy.

line 183: I'm not sure what the authors mean by 'consistent' in this sentence. Wasn't the ploidy decided from genome assembly characteristics? In this case it's expected to be consistent.

line 195: I would suggest explaining here in once sentence how SNP calling was made, especially how the reference was constructed, because it's quite important for interpreting the results.

line 202: PCA plots in Fig1: It would be useful to add ploidy information in the plot to see where are those samples located relative to haploids. Are these clusters explained by geography or habitat? Perhaps adding this information would be useful as well. It is also not mentioned anywhere how divergent are these clusters. It would be worth reporting nucleotide divergence between (haploid) genomic groups.

line 221: Please highlight haploids/diploid on the phylogeny.

line 209: Considering LD decay analysis it looks to me that r2 is very low even between close variants. In general, it is not clear from the figure 2 what is the maximum r2 between adjacent pairs of SNPs (start of the line) and what is the distance over which r2 falls by half. I think the authors should give some quantification of this in the results. This could give a better understanding of the LD.

line 229: This is a really interesting way to show relationships between hybrids!

line 250: Fig 5, What are colours in the legend signifying?

line 282: MAT loci: One option to make sure if MAT loci is truly absent would be to look for reads matching the sequence of MAT. This could eliminate the possibility that the quality of an assembly is a source of missing loci.
line 263: In the figures S4 and S5 one information that is missing is whether the same MAT type is present always on the same genomic background, assuming that these different types are in the same locus. Examples of MAT introgression are common in fungi so it would be nice to check if something like this occurs in these species.
line 619: In the description of processing sequence alignments, please specify what do you mean by "long gaps"? Was there any threshold?

Methods

Are the methods appropriate to the aims of the study, are they well described, and are necessary controls included? Choose an item.

Conclusions

Are the conclusions adequately supported by the data shown? Choose an item.

Reporting Standards

Does the manuscript adhere to the journal's guidelines on minimum standards of reporting? Choose an item.

Choose an item.

Statistics

Are you able to assess all statistics in the manuscript, including the appropriateness of statistical tests used? Choose an item.

Quality of Written English

Please indicate the quality of language in the manuscript: Choose an item.

Declaration of Competing Interests

Please complete a declaration of competing interests, considering the following questions:

- Have you in the past five years received reimbursements, fees, funding, or salary from an organisation that may in any way gain or lose financially from the publication of this manuscript, either now or in the future?
- Do you hold any stocks or shares in an organisation that may in any way gain or lose financially from the publication of this manuscript, either now or in the future?

- Do you hold or are you currently applying for any patents relating to the content of the manuscript?
- Have you received reimbursements, fees, funding, or salary from an organization that holds or has applied for patents relating to the content of the manuscript?
- Do you have any other financial competing interests?
- Do you have any non-financial competing interests in relation to this paper?

If you can answer no to all of the above, write 'I declare that I have no competing interests' below. If your reply is yes to any, please give details below.

I declare that I have no competing interests

I agree to the open peer review policy of the journal. I understand that my name will be included on my report to the authors and, if the manuscript is accepted for publication, my named report including any attachments I upload will be posted on the website along with the authors' responses. I agree for my report to be made available under an Open Access Creative Commons CC-BY license (http://creativecommons.org/licenses/by/4.0/). I understand that any comments which I do not wish to be included in my named report can be included as confidential comments to the editors, which will not be published.

Choose an item.

To further support our reviewers, we have joined with Publons, where you can gain additional credit to further highlight your hard work (see: https://publons.com/journal/530/gigascience). On publication of this paper, your review will be automatically added to Publons, you can then choose whether or not to claim your Publons credit. I understand this statement.

Yes Choose an item.
