# Supplementary material for: Clonality, inbreeding, and hybridization in two extremotolerant black yeasts
Source: Gigascience. 2022 Oct 6;11:giac095. doi: 10.1093/gigascience/giac095 (PMC9535773; doi:10.1093/gigascience/giac095)
Supplement: giac095_Reviewer_2_Report_Original_Submission — Noah Gettle -- 5/18/2022 Reviewed [file giac095_reviewer_2_report_original_submission.pdf]

Reviewer Report

Title: Clonality, inbreeding, and hybridization in two extremotolerant black yeasts

Version: Original Submission **Date:** 5/18/2022

Reviewer name: Noah Gettle

Reviewer Comments to Author:

GostinÄ�ar et al. use short read sequencing to assemble the genomes of >100 strains of two disparate species of black yeast previously shown to be mainly clonal but which contain haploid as well as diploid strains. The authors use the greater number of sequenced strains to better characterize intraspecific differences in ploidy. They estimated low levels of recombination within strains but identify a number of intraspecific hybridization events which led increases in ploidy. Based on the lack of recombined or mixed haplotypes in haploid strains and a relative lack of aneuploids representing a loss of genomic regions, the authors conclude that intraspecific diploid and tetraploid hybrids do not undergo meiosis or chromosomal loss to return to a haploid state but are rather stable.
The results the authors present of stable largely non-recombinant intraspecific hybrids in two different yeast species represent an interesting and novel result that could spark important research into evolutionary losses of sex, speciation, and recombination rates. That said, I do have a couple major and minor concerns.
Major:
1. The methods are missing a description of how ploidy was estimated, the results of which are integral to the conclusions.
2. The authors should be clearer about how they use and what they mean by clonality and asexuality particularly with respect to recombination.
Minor:
1. The use of the term 'hybrid' should be preceded by the descriptor 'intraspecific' for clarity's sake
2. A clearer description of the question/hypotheses being addressed earlier in the introduction would go a long way to improve readability.

Methods

Are the methods appropriate to the aims of the study, are they well described, and are necessary controls included? Choose an item.

Conclusions

Are the conclusions adequately supported by the data shown? Choose an item.

Reporting Standards

Does the manuscript adhere to the journal's guidelines on [minimum standards of reporting?]() Choose an item.

Choose an item.

Statistics

Are you able to assess all statistics in the manuscript, including the appropriateness of statistical tests used? Choose an item.

Quality of Written English

Please indicate the quality of language in the manuscript: Choose an item.

Declaration of Competing Interests

Please complete a declaration of competing interests, considering the following questions:

- Have you in the past five years received reimbursements, fees, funding, or salary from an organisation that may in any way gain or lose financially from the publication of this manuscript, either now or in the future?
- Do you hold any stocks or shares in an organisation that may in any way gain or lose financially from the publication of this manuscript, either now or in the future?
- Do you hold or are you currently applying for any patents relating to the content of the manuscript?
- Have you received reimbursements, fees, funding, or salary from an organization that holds or has applied for patents relating to the content of the manuscript?
- Do you have any other financial competing interests?
- Do you have any non-financial competing interests in relation to this paper?

If you can answer no to all of the above, write 'I declare that I have no competing interests' below. If your reply is yes to any, please give details below.

I declare that I have no competing interests

I agree to the open peer review policy of the journal. I understand that my name will be included on my report to the authors and, if the manuscript is accepted for publication, my named report including any attachments I upload will be posted on the website along with the authors' responses. I agree for my report to be made available under an Open Access Creative Commons CC-BY license (http://creativecommons.org/licenses/by/4.0/). I understand that any comments which I do not wish to be included in my named report can be included as confidential comments to the editors, which will not be published.

Choose an item.

To further support our reviewers, we have joined with Publons, where you can gain additional credit to further highlight your hard work (see: https://publons.com/journal/530/gigascience). On publication of this paper, your review will be automatically added to Publons, you can then choose whether or not to claim your Publons credit. I understand this statement.

Yes Choose an item.
